# Hsp90*β*‐Selective Inhibitors: Probing the Solvent‐Accessible Frontier

**DOI:** 10.1002/cmdc.202500657

**Published:** 2025-11-18

**Authors:** Terin D’Amico, Michael A. Serwetnyk, Xiaozheng Dou, Ian Mersich, Deborah Barlow, Karen L. Houseknecht, John M. Streicher, Aktar Ali, Brian S. J. Blagg

**Affiliations:** ^1^ Department of Chemistry and Biochemistry University of Notre Dame Notre Dame Indiana 46556 USA; ^2^ Warren Center for Drug Discovery University of Notre Dame Notre Dame Indiana 46556 USA; ^3^ Department of Biomedical Sciences Portland Laboratory for Biotechnology and Health Sciences University of New England Portland Maine 04103 USA; ^4^ Department of Pharmacology College of Medicine and the Comprehensive Center for Pain and Addiction University of Arizona Tucson Arizona 85724 USA

**Keywords:** anticancer, hsp90b, inhibitor design, optimization, ATP‐binding pocket

## Abstract

Inhibitors of the 90‐ kDa heat shock protein (Hsp90) family, especially Hsp90*β*, have been a sought‐after therapeutic strategy for the treatment of cancer, neurological disorders, and other diseases. Furthermore, recent studies suggest that their coadministration with other therapies can enhance efficacy. *pan‐*Inhibition of the cytosolic Hsp90*α* and Hsp90*β* isoforms has proven to be problematic, since the on‐target toxicities have resulted in the failure of most Hsp90 inhibitors that entered clinical trials. Consequently, such outcomes highlight the demand for isoform‐selective inhibitors that overcome these detriments. Previously, we reported that subtle modifications to the solvent‐exposed region of Hsp90*β*‐selective inhibitors can significantly impact affinity and selectivity. Consequently, nineteen additional analogs were synthesized and evaluated for their ability to bind the cytosolic Hsp90 isoforms, as well as elucidate further structure–activity relationships (SAR) at this region of the molecule. The work herein reveals the extent to which appendages with steric bulk are tolerated, as well as the importance of heteroatoms to maintain high Hsp90*β* affinity and selectivity. Biological evaluation of these compounds supports the selective inhibition of Hsp90*β* in cellulo, which is encouraging for the continued exploration of Hsp90 isoform‐selective inhibitors for therapeutic applications.

## Graphical abstract

The piperidinol ring of the **KUNB106** scaffold (boxed in red) occupies the solvent‐exposed region of the Hsp90*β* ATP‐binding pocket, which provides a unique opportunity to optimize this region of the molecule. In the study disclosed herein, modifications such as C—C bonds, heteroatoms, and three‐dimensionality result in improved inhibitors that further support the therapeutic advantages of Hsp90*β*‐selective inhibition.

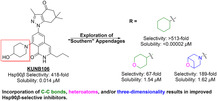

## 1 Introduction

Heat shock proteins (Hsps) are a crucial group of molecular chaperones that are responsible for maintaining cellular homeostasis by folding nascent and denatured proteins into biologically active conformations. Among the various Hsps, Hsp90 stands out as the most prevalent because it accounts for ≈1 to 2% of the total cytosolic protein mass in normal cells. Under conditions of cellular insults or stress, the levels of Hsp90 can more than double to refold proteins that have become denatured. Hsp90 comprises four isoforms: inducible Hsp90*α* and constitutively expressed Hsp90*β* in the cytosol, 94‐ kDa glucose‐regulated protein (Grp94) associated within the endoplasmic reticulum, and TNF receptor‐associated protein 1 (TRAP1) localized to the mitochondria. These chaperones collectively regulate the conformational maturity of over 400 protein substrates, many of which are regulators of oncogenic transformation, growth, metastasis, and are closely linked to the ten hallmarks of cancer.^[^ ^1^ ^,^ ^2^ ^]^ As a result, inhibition of Hsp90 leads to the degradation of Hsp90‐dependent client proteins *via* the ubiquitin‐proteasome pathway and the disruption of multiple oncogenic pathways.

All four isoforms of Hsp90 share common structural elements, including a highly conserved N‐terminal domain that exhibits ATPase activity, a middle domain responsible for protein–protein interactions, and a C‐terminal domain critical for homodimerization. Additionally, the Hsp90 homodimer interacts with various ancillary proteins that results in the formation of a heteroprotein complex that plays a vital role in the folding of client proteins.^[^ ^3^ ^]^

The Hsp90 family regulates the maturation of various cellular and extracellular proteins, including kinases, growth factors, receptors, and transcription factors.^[^ ^4^ ^,^ ^5^ ^]^ It plays a crucial role in stabilizing mutated proteins, making it a key target for disease mitigation.^[^ ^6^ ^]^ However, *pan*‐inhibition of all four isoforms (Hsp90*α*, Hsp90*β*, Grp94, and TRAP1) has been an unsuccessful therapeutic approach due to dose‐limiting toxicities and induction of the pro‐survival heat shock response.^[^ ^7^, ^8^, ^9^, ^10^, ^11^, ^12^ ^–^ ^13^ ^]^ To address these issues, isoform‐selective inhibitors have been explored, with Hsp90*β* emerging as a promising target due to its distinct substrates and lack of cardio‐ and ocular‐toxicity that is associated with *pan*‐inhibition.^[^ ^14^, ^15^, ^16^ ^–^ ^17^ ^]^

Hsp90*β* is essential for early cell survival and development, including placental formation in mammals,^[^ ^18^ ^]^ and plays a role in cancer progression, with aberrant activity observed in transformed cells.^[^ ^19^ ^]^ Studies suggest that Hsp90*β* is preferentially involved in leukemic cell survival and interacts with tumor suppressor p53 differently than Hsp90*α*.^[^ ^20^, ^21^ ^–^ ^22^ ^]^ Additionally, Hsp90*β* levels correlate with lung cancer severity and is implicated in endothelial cell growth, gastric cancer, multiple myeloma, and colon cancer metastasis.^[^ ^23^, ^24^, ^25^, ^26^, ^27^ ^–^ ^28^ ^]^ It also modulates matrix metalloproteinases (ex: MMP3) in mammary epithelial cells, influencing tumor invasion.^[^ ^29^ ^]^

**Figure 1:**
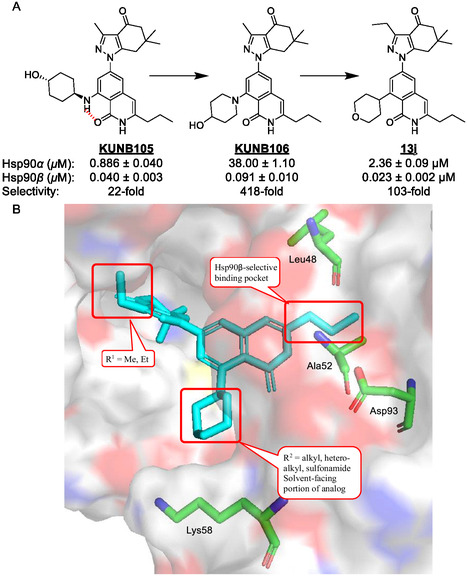
A) Structures and apparent K_D_ values of **KUNB105**, **KUNB106,** and **13j,** as determined via a fluorescence polarization (FP) assay. B) Proposed binding mode of **13j** within the Hsp90*β*‐binding site (PDB code: 1UYM^[^ ^39^ ^]^).

While Hsp90*β* has been shown to be important for viral and metabolic disorders, it is also known to play a key role in CNS disorders. For example, Hsp90 is also implicated in neurodegenerative diseases such as Alzheimer's, Parkinson's, and Huntington's diseases, where its role in degrading misfolded proteins provides neuroprotection.^[^ ^30^, ^31^ ^–^ ^32^ ^]^ Furthermore, coadministration of Hsp90*β*‐selective inhibitors, such as **KUNB106** (**Figure** **1**A), have demonstrated enhanced antinociceptive effects of morphine in mice by activating the ERK‐RSK pathway, specifically within the spinal cord independent of the brain and periphery.^[^ ^33^, ^34^ ^–^ ^35^ ^]^ The underlying activity was subsequently understood to result from the suppression of an AMPK‐mediated negative feedback loop.^[^ ^36^ ^]^ Whereas activation of ERK enhanced antinociceptive activity, activation of AMPK suppresses ERK. It is noteworthy that the enhanced efficacy of morphine manifested by spinal cord Hsp90 inhibition comes without an increase in tolerance nor constipation. In addition, it was recently reported that selective inhibitors of Hsp90*β* decreased lipopolysaccharide (LPS)‐induced production of inflammatory mediators (NO, IL‐1*β*, and TNF‐*α*) in murine microglial BV‐2 cells by attenuating NF‐*κ*B and ERK‐MAPK activation.^[^ ^37^ ^]^ In contrast, inhibitors that target Hsp90*α*, Grp94, and TRAP1 had minimal impact on these mediators, highlighting Hsp90*β* as central to LPS‐induced neuroinflammation and therefore represents a promising drug target for the development of pain medications with potentially fewer adverse effects.

While the N‐terminal, ATP‐binding pockets are >85% identical across all four isoforms, those of cytosolic Hsp90*α* and Hsp90*β* differ by only two amino acids (Hsp90*β* contains Ala‐52 and Leu‐91 in lieu of Ser‐52 and Ile‐91 for Hsp90*α*). Despite their high identity, the differences between these residues are sufficient to provide a sub‐pocket in Hsp90*β* that does not exist in Hsp90*α*. Therefore, exploitation of this pocket was accomplished *via* the development of a series of Hsp90*β*‐selective inhibitors that manifest apparent K_D_ values in the low nM range and >300‐fold selectivity over Hsp90*α*.^[^ ^38^ ^]^

One compound that exhibited such remarkable affinity is KUNB106**,** which projects an *n*‐propyl moiety into the Hsp90*β*‐exclusive sub‐pocket formed by the enhanced flexibility of Leu‐91 over the Ile‐91 of Hsp90*α* (Figure 1A). Studies have shown this alkyl tail length to be optimal, as larger chains diminish affinity, while smaller ones confer less selectivity towards Hsp90*β*. Meanwhile, the piperidinol moiety of KUNB106 occupies a solvent‐exposed region within the Hsp90*β* binding pocket that enables interactions with water molecules and ‘gateway’ residues such as Lys‐58.^[^ ^38^ ^]^ Together, these observations present an opportunity to optimize the solvent‐exposed region of the **KUNB106** scaffold *via* the introduction of moieties with greater flexibility (i.e., C(*sp* ^*3*^) centers, rotatable bonds, etc.) and/or polarity to promote more favorable interactions, elucidate further SAR, and improve other properties such as solubility.

## 2 Experimental Section

### 2.1 Chemistry

#### 2.1.1 General Information

All reagents and solvents were acquired from commercial sources. All reactions were performed in oven‐ or flame‐dried glassware under an argon atmosphere, unless otherwise stated. By consulting each reagent's material safety data sheet (MSDS), there were no new or unexpected safety hazards or risks associated with the reported syntheses, methods/conditions, or biological evaluation of compounds contained herein. Flash column chromatography was performed using silica gel (40–63 µM particle size). ^1^H‐ and ^13^C‐NMR spectra were recorded at 400 and 101 MHz, respectively, on a Bruker AVANCE III HD 400 Nanobay instrument. Coupling constants (*J*) are reported in Hertz, chemical shifts are referenced to the residual deuterated solvent peak, and peak *δ* values are reported in parts per million ( ppm). High‐resolution mass spectral data were obtained on a Bruker MicrOTOF II time‐of‐flight mass spectrometer, and analysis was performed using electrospray ionization. Thin‐layer chromatography was performed with glass‐backed, TLC silica gel 60F254 plates purchased from Millipore Sigma and visualized by UV light, at 254 nm. Reactions that required microwave irradiation were performed with a CEM Discover/Explorer 12 microwave reactor.

#### 2.1.2 Synthesis of Intermediates and Compounds

##### 2.1.2.1 Synthesis of Compounds 8a–j: General Synthesis of Acylated Dimedones 2a and 2b

Acetic or propionic anhydride (3.92 mmol, 1.1 equiv.) was added dropwise to a solution of commercially available dimedone 1 (3.57 mmol, 1 equiv), DMAP (1.07 mmol, 0.3 equiv.), and DIPEA (3.92 mmol, 1.1 equiv.) in DCM (4.0 mL), and the reaction was stirred for 20 h at 40 °C. Upon cooling to room temperature, the reaction was quenched with aqueous, 1 N HCl (4.0 mL). The aqueous layer was extracted with DCM (3 x 2.0 mL), and the combined organic fractions were washed with water (5.0 mL), then with brine (5.0 mL), dried over sodium sulfate, filtered, and concentrated in vacuo. The crude residue, an orange‐colored oil, was utilized in subsequent reactions without further purification or characterization.

##### 2.1.2.2 Synthesis of Compounds 8a–j: Synthesis of 2,6‐Difluoro‐4‐Hydrazineylbenzonitrile 4

Hydrazine monohydrate (≈55% solution) (6.37 g, 127 mmol, ≈2 equiv.) was added dropwise to a pressure vessel filled with a solution of commercially available 2,4,6‐trifluorobenzonitrile 3 (10.0 g, 63.7 mmol, 1 equiv.) in ethanol (100 mL). The reaction was stirred at 60 °C, until the complete consumption of starting material was observed, as determined via TLC (≈4 h). Upon cooling to room temperature, the solvent was removed in vacuo*,* and the remaining solid was resuspended in water (100 mL). The aqueous layer was extracted with ethyl acetate (3 x 75 mL), and the combined organic fractions were washed with water (100 mL), then with brine (100 mL), dried over sodium sulfate, filtered, and concentrated in vacuo. The residue was purified via column chromatography (2% MeOH in DCM) to give the product as a colorless solid.

##### 2.1.2.3 Synthesis of Compounds 8a–j: Synthesis of Pyrazole 5

Functionalized dimedone 2a (538 mg, 2.96 mmol, 1 equiv.) was added to a pressure vessel charged with a solution of 2,6‐difluoro‐4‐hydrazineylbenzonitrile 4 (500 mg, 2.96 μmol, 1 equiv.) in EtOH (7.5 mL). The tube was sealed, and the reaction was stirred at 100 °C for 6 h. Upon cooling to room temperature, the solvent was removed in vacuo, and the remaining matter was resuspended in ethyl acetate (25.0 mL). The organic layer was washed with water (25.0 mL), then with brine (25.0 mL), dried over sodium sulfate, filtered, and concentrated in vacuo. The residue was purified via column chromatography (5–40% ethyl acetate in hexanes) to give the product as a light‐yellow solid.

##### 2.1.2.4 Synthesis of Compounds 8a–j: Synthesis of Isoquinolinone 7

Methyl‐3‐oxohexanoate (164 mg, 1.14 mmol, 1.2 equiv) was added to a solution of pyrazole 5 (300 mg, 951 µmol, 1 equiv.) and potassium carbonate (158 mg, 1.14 mmol, 1.2 equiv.) in DMF (4.25 mL). The reaction was stirred at 70 °C until the complete consumption of starting material was observed, as determined via TLC (≈16 h). Upon cooling to room temperature, the solution was acidified to pH 5 with aqueous, 1N HCl, which was followed by the addition of saturated, aqueous ammonium chloride (12 mL). The aqueous layer was extracted with ethyl acetate (3 x 12 mL). The combined organic fractions were washed with water (3 x 30 mL), then with brine (30 mL), dried over sodium sulfate, filtered, and concentrated in vacuo. The residue (crude intermediate 6), an orange‐colored oil, was utilized in the next step without further purification or characterization.

The crude material from the previous step (≈400 mg) was added to a 25 mL pressure vessel and dissolved in 2.8 mL of sulfuric acid (36 N), 1.4 mL of water, and 9.8 mL of glacial acetic acid (H_2_SO4:H_2_O:AcOH = 2:1:7). IMPORTANT NOTE: The solution was first stirred at 80 °C for 1 h under an open atmosphere to ensure the escape of CO_2_ that forms from the decarboxylation reaction and to avoid an excess buildup of pressure. After this time, the vessel was sealed, and the reaction was then stirred at 130 °C for an additional 12 h. Upon cooling to room temperature, the reaction was quenched by pouring the solution into 100 mL of water. The resulting precipitate was collected and dried via vacuum filtration and then dissolved in ethyl acetate (25 mL). The residue was loaded directly onto silica gel and purified via column chromatography (12–100% ethyl acetate in hexanes) to give the product as a light‐brown solid.

##### 2.1.2.5 Synthesis of Compounds 8a–j: General Syntheses of Amine‐ or Sulfonamide‐Containing Compounds 8a–j

Procedure A (for amine‐containing 8a–b and 8e–j): DIPEA (for amines: 0.21 mmol, 3 equiv.; for ammonium hydrochloride salts: 0.42, 6 equiv.) was added to a pressure vessel charged with intermediate 7 (69 µmol, 1 equiv.) and the appropriate amine/ammonium hydrochloride salt (0.21 mmol, 3 equiv.) dissolved in DMSO (600 µL). The vessel was sealed, and the reaction was stirred at 140 °C for 12 h. Upon cooling to room temperature, water (25 mL) was added to the solution, and the aqueous layer was extracted with ethyl acetate (3 x 25 mL). The combined organic fractions were washed with water (5 x 25 mL), then with brine (25 mL), dried over sodium sulfate, filtered, and concentrated in vacuo. The residue was purified via column chromatography (5% MeOH in DCM) and then further purified via preparatory TLC (3% MeOH in DCM) to give the final products as light‐yellow solids.

Procedure B (for sulfonamides 8c and 8d): potassium carbonate (0.31 mmol, 6 equiv.) and the appropriate sulfonamide (0.31 mmol, 6 equiv.) were added to a pressure vessel charged with a solution of 7 (52 µmol, 1 equiv.) in DMSO (100 µL). The vessel was sealed, and the reaction was stirred at 140 °C for 6 h. Upon cooling to room temperature, the reaction was quenched with water (10 mL) and then extracted with ethyl acetate (3 x 10 mL). The combined organic fractions were washed with water (5 x 10 mL), then with brine (10 mL), dried over sodium sulfate, filtered, and concentrated in vacuo. The residue was purified via column chromatography (2% MeOH in DCM) and then further purified via preparatory TLC (2% MeOH in DCM) to give the products as light‐yellow solids.

#### 2.1.3 Synthesis of Compounds 13a–j

##### 2.1.3.1 General Synthesis of Indazolones 9a–b

Acylated dimedone 2a or 2b (61 mmol, 1.1 equiv.) was added to a pressure vessel and dissolved in ethanol (125 mL). Hydrazine hydrate (≈55% solution) (55 mmol, 1 equiv.) was added to the solution, and the reaction was stirred at 100 °C for 12 h. Upon cooling to room temperature, the solvent was removed in vacuo, and remaining matter was resuspended in ethyl acetate (50 mL). The organic layer was washed with water (50 mL), then with brine (50 mL), dried over sodium sulfate, filtered, and concentrated in vacuo. The residue was purified via column chromatography (2% MeOH in DCM) to give the products as light‐yellow solids.

##### 2.1.3.2 Synthesis of 6,8‐Difluoro‐3‐Propylisoquinolin‐1(2H)‐One 11

Methyl‐3‐oxohexanoate (5.50 mmol, 1.2 equiv.) was added to a solution of benzonitrile 3 (4.59 mmol, 1 equiv.) and potassium carbonate (5.50 mmol, 1.2 equiv.) in DMF (20 mL). The reaction was stirred at 70 °C until the complete consumption of starting material was observed, as determined via TLC (≈16 h). Upon cooling to room temperature, the solution was acidified to pH 5 with aqueous, 1 N HCl, followed by the addition of saturated, aqueous ammonium chloride (20 mL). The aqueous layer was extracted with ethyl acetate (3 x 20 mL). The combined organic fractions were washed with water (5 x 20 mL), then with brine (20 mL), dried over sodium sulfate, filtered, and concentrated in vacuo. The residue (crude intermediate 10), an orange/brown‐colored oil, was utilized in the next reaction without further purification or characterization.

The crude material from the previous step was added to a 50 mL pressure vessel and dissolved in 5.0 mL sulfuric acid (36 N), 2.5 mL of water, and 18 mL of glacial acetic acid (H_2_SO_4_:H_2_O:AcOH = 2:1:7). IMPORTANT NOTE: The solution was first stirred at 80 °C for 1 h under an open atmosphere to ensure the escape of CO_2_ that forms from the decarboxylation reaction and to avoid an excess buildup of pressure. The vessel was sealed, and the reaction was then stirred at 130 °C for 12 h. Upon cooling to room temperature, the reaction was quenched by pouring the solution into 200 mL of water. The aqueous layer was neutralized with saturated, aqueous sodium bicarbonate and extracted with ethyl acetate (3 x 75 mL). The combined organic fractions were washed with water (100 mL), then with brine (100 mL), dried over sodium sulfate, filtered, and concentrated in vacuo. The residue was purified via column chromatography (20% ethyl acetate in hexanes) to give the product as a brown solid.

##### 2.1.3.3 General Synthesis of Ring Systems 12a–h

A freshly prepared 1.0 M solution of Grignard reagent (1.79 mmol, 4 equiv.) in THF (5.0 mL), made from the respective alkyl bromide precursor, was added to a solution of intermediate 11 (100 mg, 448 µmol, 1 equiv.) in THF (1.0 mL). The reaction was heated to 110 °C via microwave irradiation for 15 min to 1 h until consumption of the starting material was observed via TLC. Upon cooling to room temperature, the reaction was quenched with water (5.0 mL) and extracted with ethyl acetate (5.0 mL). The combined organic fractions were washed with water (5 x 5.0 mL), then with brine (5.0 mL), dried over sodium sulfate, filtered, and concentrated in vacuo. The crude residue was utilized in subsequent reactions without further purification or characterization.

##### 2.1.3.4 General Synthesis of Compounds 13a–j

Sodium hydride (60% weight, 518 µmol, 1.5 equiv.) in DMF (3.0 mL) was added to a microwave vial filled with a solution of indazolone 9a or 9b (518 µmol, 1.5 equiv.) in DMF (3.0 mL). The reaction was stirred at room temperature for 15 min, after which intermediate 12a–h (346 µmol, 1 equiv.) in DMF (4.0 mL) was added. The reaction was heated to 140 °C via microwave irradiation and stirred at this temperature for 16 h. Upon cooling to room temperature, the reaction was quenched with water (5.0 mL) and extracted with ethyl acetate (3 x 10 mL). The combined organic fractions were washed with water (5 x 10 mL), then with brine (10 mL), dried over sodium sulfate, filtered, and concentrated in vacuo. The residue was purified via column chromatography (0–50% ethyl acetate in hexanes) to give the products as colorless solids.

### 2.2 Fluorescence Polarization Assay Procedure

The FP assay was performed in 96‐well format in black, flat‐bottom plates (Santa Cruz Biotechnology) with a final volume of 100 μL. Twenty‐five microliters of assay buffer (20 mM HEPES, 50 mM KCl, 10.5 mM MgCl_2_, 20 mM Na_2_MoO_4_, 0.01% NP‐40 detergent (NP‐40), and pH 7.3 with fresh 2 mM dithiothreitol (DTT) and 0.1 mg mL^−1^ bovine *γ*‐globulin (BGG) added before use), 25 μL of assay buffer containing 6 nM FITC‐GDA (fluorescent tracer, stock in DMSO and diluted in assay buffer), and 50 μL of assay buffer containing 10 nM of Hsp90*α* and Hsp90*β* were added to each well. Compounds were tested in triplicate wells (1% DMSO final concentration). For each plate, wells containing buffer only (background), tracer in buffer only (low polarization control), and protein and tracer in buffer with 1% DMSO (high polarization control) were included. Plates were incubated at 4 °C with rocking for 24 h. Polarization values (in mP units) were measured at 37 °C with an excitation filter at 485 nm and an emission filter at 528 nm. Polarization values were correlated to % tracer bound and compound concentrations. The concentration at which the tracer was 50% displaced by the compound of interest (IC_50_) was determined using Microsoft Excel and reported as an average and standard deviation from triplicate runs.

### 2.3 Evaluation of Aqueous Solubility

Aqueous solubility was determined using a miniaturized shake flask approach, under condition of pH 6.8 and analyte concentration of 1.0 mM by the method of Zhou et al.^[^ ^40^ ^]^ Aqueous solutions of analyte were incubated at room temperature in the chamber of a Whatman (Piscataway, NJ) Mini‐UniPrep syringeless filter for 24 h while shaking gently (600 rpm). Subsequent to incubation, filter plungers were pushed down to the bottom of the syringeless filter chamber assemblies, allowing filtrate to enter the plunger compartment. Following an additional 30 min incubation at room temperature, filtrates were diluted with 50:50 acetonitrile/water +0.1% formic acid and analyzed by LC–MS/MS. Analyte concentrations were determined by the interpolation of peak area ratio from a calibration curve formed by matrix spiked with authentic reference material.

### 2.4 General Cell Culture Procedures

A549, MDA‐MB‐231, and SKOV‐3 cells were grown in a water‐jacketed incubator at 37 °C with 5% CO_2_. A549, MDA‐MB‐231, and SKOV‐3 cells were cultured in RPMI‐1640, DMEM, and McCoy's media, respectively, all of which were supplemented with 10% HI FBS (Gibco, 10,438–026) and 1% Pen‐Strep (VWR, K952‐100 mL)

### 2.5 MTS Assay Procedure

A549, MDA‐MB‐231, and SKOV‐3 cells were grown to confluence and then seeded in clear, flat‐bottomed 96‐well plates at a density of 1000 cells per well. After an overnight attachment period, cells were treated either with 0.5% DMSO (vehicle control) or compound. The number of viable cells was determined at 72 h using the CellTiter 96 Aqueous One Solution Cell Proliferation assay kit (Promega Corp., Madison, WI), in accordance with the manufacturer's instructions. Twenty microliters of CellTiter 96 solution was added to each well, and the plates were incubated for 3.25 h at 37 °C, after which the absorbance of each well was measured at a wavelength of 490 nm with a microplate spectrophotometer (Biotek Epoch). All assay runs were performed in triplicate, and the antiproliferative values for each compound were determined from 3 independent experiments. Cell of treated plates was calculated relative to an untreated time zero plates measured at the time of treatment. Growth inhibition curves, half‐maximal inhibitory concentrations (IC_50_), and statistical analyses were calculated with GraphPad Prism V9.

### 2.6 Western Blot Procedure

MDA‐MB‐231 cells were grown in DMEM/F‐12 (Gibco, 11,320,033) media containing 1% Pen‐Strep (VWR, K952‐100 ML) and 10% FBS+ (Atlas Biologicals, F‐0500‐D) in a water‐jacketed incubator at 37 °C with 5% CO_2_. Approximately 300,000 cells were seeded into 6‐well plates and grown to ≈80% confluency. Media was replaced with fresh media containing compounds of interest at the indicated concentrations, or vehicle control for 24 h.

Cells were lysed using RIPA buffer‐containing protease and phosphatase inhibitors. Protein concentration in lysates were determined by BCA assay (Thermo). Proteins were separated by SDS‐PAGE using 10% SDS‐polyacrylamide gels and transferred to nitrocellulose membranes. Transfer efficiency and total protein evaluated by ponceau staining. Membranes were blocked with 7% nonfat dry milk solution and probed with antibodies raised against GAPDH, Hsp90, Hsp70, CDK4, (Cell Signaling, 12,790), CDK6 (Cell Signaling, 3136), and Akt (Cell Signaling, 9272). Antibody dilutions were 1:1000 and secondary antibodies were used at 1:5000 (Southern Biotech). Blots were developed using ECL (Amersham, 45‐000−999) on a ChemiDoc Imaging System (BioRad).

### 2.7 Procedure for Molecular Modeling and Docking Studies

The Maestro program from Schrödinger was used for the molecular modeling and docking studies. The cocrystal structure of PU‐3 bound to Hsp90*β* (PDB: 1UYM^[^ ^39^ ^]^) was utilized for modeling experiments. PyMOL was used for further visualization and in the preparation of Figure 1B.

## 3 Results and Discussion

### 3.1 Design and Synthesis of KUNB106 Analogs

**Scheme 1:**
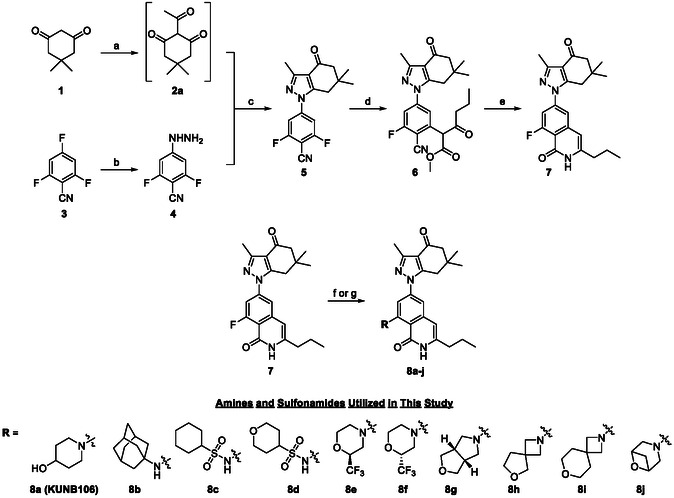
Synthesis of amine‐ and sulfonamide‐bearing analogs of **KUNB106**. Reagents and conditions: a) acetic anhydride, DMAP, DIPEA, DCM, 40 °C, 20 h. b) NH_2_NH_2_·H_2_O, Ethanol, 60 °C, 4 h, 65%. c) Ethanol, 100 °C, 6 h, 84%. d) Methyl‐3‐oxohexanoate, K_2_CO_3_, DMF, 70 °C, 16 h. e) H_2_SO_4_/water/acetic acid (2:1:7), 130 °C, 12 h, 28% (over 2 steps). f) Amine or ammonium hydrochloride salt, DIPEA, DMSO, 140 °C, 12 h, 41–86%. g) Sulfonamide, K_2_CO_3_, DMSO, 140 °C, 6 h, 20–22%.

In our previous report on the SAR of isoquinolinone‐based Hsp90*β*‐selective inhibitors, **KUNB105**, which bears a *trans*‐4‐aminocyclohexanol ring as the ‘southern’ appendage, was found to bind Hsp90*α* and Hsp90*β* with apparent K_D_'s of 0.886 and 0.040 µM, respectively. These values correspond to ≈22‐fold selectivity towards Hsp90*β*; however, replacement of this group with secondary amines resulted in compounds, such as piperidinol‐containing **KUNB106**, that exhibit improved (418‐fold) selectivity (Figure 1A).^[^ ^38^ ^]^ These data reveal the striking influence that the solvent‐exposed moiety imparts on affinity/selectivity towards Hsp90*β*. Additionally, such modifications are likely to enhance the solubility of these inhibitors, since the amine of **KUNB105** restricts bond rotation via participation in an intramolecular hydrogen bond with the adjacent lactam (Figure 1A, as demonstrated by the red dashed line). Therefore, we sought to further investigate this hypothesis via the incorporation of more polar and/or 3D amines and sulfonamides into the **KUNB106** scaffold. Synthesis of these initial derivatives is represented in **Scheme** **1**, which aligns with our previously established route.^[^ ^38^ ^,^ ^41^ ^]^ For example, the reaction between commercially available dimedone **1** and acetic anhydride in the presence of DIPEA and DMAP in DCM gave the 2‐acylated product, **2a**. As described elsewhere, these conditions were necessary to ensure complete conversion of the kinetic *O*‐acylated product into the thermodynamic *C*‐acylated adduct.^[^ ^41^ ^]^ Meanwhile, 2,4,6‐trifluorobenzonitrile **3** underwent an S_*N*_Ar with hydrazine monohydrate, which results in the formation of both *ortho‐* and *para‐*substituted products. Although we have previously found success in carrying forward the crude mixture, column chromatography was utilized to isolate the desired *para*‐substituted product, **4**. Intermediates **2a** and **4** were then combined in ethanol at reflux to generate the pyrazole core of benzonitrile, **5**. The benzonitrile then underwent a second S_*N*_Ar with methyl‐3‐oxohexanoate and potassium carbonate in DMF to install the *β*‐ketoester moiety. Upon exposure to heat and acidic conditions, a simultaneous hydrolysis‐decarboxylation‐cyclization occurred to form the requisite isoquinolinone ring system of **7**. Lastly, aryl fluoride intermediate **7** was subjected to a third S_*N*_Ar reaction with various amines and sulfonamides to achieve the final products, **8a–j**.

**Scheme 2:**
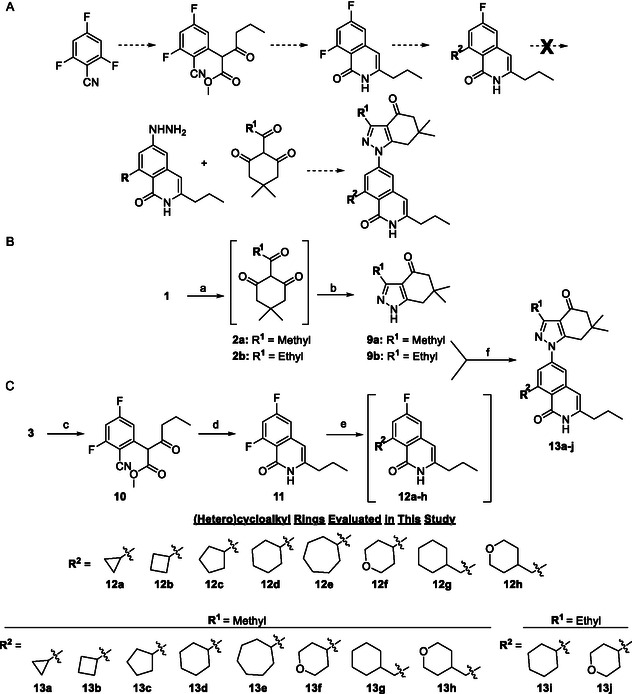
“Bottom up” approaches towards the synthesis of **KUNB106** analogs via Grignard reagents. A) Our initially proposed, but unsuccessful, approach. B) Synthesis of “northern” indazolone fragments. Reagents and conditions: a) Acetic or propionic anhydride, DMAP, DIPEA, DCM, 40 °C, 20 h. b) NH_2_NH_2_·H_2_O, Ethanol, 100 °C, 12 h, 80–85% (over 2 steps). C) Synthesis of “bottom” ring systems. Reagents and conditions: c) methyl‐3‐oxohexanoate, K_2_CO_3_, DMF, 70 °C, 16 h. d) H_2_SO_4_/water/acetic acid (2:1:7), 130 °C, 12 h, 20% (over 2 steps). e) Grignard reagent (1.0 molar), THF, 110 °C (microwave), 0.25–3 h. f) NaH, DMF, 140 °C (microwave), 16 h, 5–11% (over 2 steps).

Although amines can improve compound solubility via the introduction of ionizable centers, we have remained interested in exploring a wide range of substituents at this position to better understand SAR for this scaffold. Therefore, the preparation of **KUNB106** derivatives in which appendages are connected via C—C bonds was also explored using the general synthetic routes depicted in **Scheme** **2**. Grignard reagents were found to facilitate the rapid addition of such nucleophiles and provide opportunities for diversification.

As described in Scheme 1, synthesis of our Hsp90*β*‐selective inhibitors has involved a ‘top down’ approach. This route provides an aryl fluoride intermediate, such as **7**, through which S_*N*_Ar reactions enable a late‐stage generation of diverse inhibitors. However, the use of Grignard reagents complicated this approach, due to their potential reactivity with the indazolone ring system, either through nucleophilic addition or abstraction of the *α*‐proton. Therefore, initial attempts to circumvent this issue sought to reverse the order of our established route via construction of the bottom ring system, a S_*N*_Ar reaction with hydrazine, and then condensation with 2‐acyl dimedones (Scheme 2A). Unfortunately, it was observed that the fully assembled bottom ring systems become too deactivated to react with hydrazine. Consequently, we then pursued an alternative ‘bottom up’ approach that involves separate formation of the top and bottom ring systems, followed by a late‐stage coupling reaction (Scheme 2B,C).

Synthesis of ‘northern’ indazolone ring systems is depicted in Scheme 2B. Following the reaction of dimedone **1** with either acetic or propionic anhydride, acylated species **2a** and **2b** were treated with hydrazine to assemble 3‐methyl and 3‐ethyl indazolones, **9a** and **9b**, respectively. Meanwhile, preparation of ‘southern’ ring systems that harbor structural diversity is represented in Scheme 2C. 2,4,6‐Trifluorobenzonitrile **3** was similarly reacted with methyl‐3‐oxohexanoate, and then subsequently exposed to heat and acidic conditions to form isoquinolinone, **11**. A second S_*N*_Ar reaction between difluorinated species **11** with the appropriate Grignard reagent was then utilized to produce ring systems, **12a–h**. We anticipate that this addition selectively occurs at the position *ortho‐* to the lactam, which has been demonstrated by the synthesis of prior Hsp90 inhibitors with amines, Grignards, and other nucleophiles at this position.^[^ ^41^, ^42^ ^–^ ^43^ ^]^ Lastly, indazolones **9a** and **9b** and ring systems **12a–h** were coupled together via a sodium hydride‐mediated S_*N*_Ar reaction to achieve final products, **13a–j**. Because the bottom ring systems become more electron‐rich with increased substitution (i.e., less amenable to S_*N*_Ar reactions), microwave irradiation was necessary to accomplish these final two steps. Although this approach was successful, we caution that the final S_*N*_Ar with pre‐formed indazolones is not ideal due to poor yields and the generation of difficult‐to‐separate regioisomers. Consequently, some of these undesired 2*H*‐indazolone addition products were also isolated, characterized, and subjected to biological evaluation (refer to the Supporting Information for further details).

### 3.2 Evaluation of Hsp90 Binding Affinity

Upon preparation of these **KUNB106** derivatives, a fluorescence polarization (FP) assay was conducted to determine their affinity and selectivity towards Hsp90*α* and Hsp90*β* (**Table** **1**).^[^ ^44^ ^]^ **KUNB106** exhibits apparent K_D_ values of 38 and 0.091 µM against Hsp90*α* and Hsp90*β*, respectively, which corresponds to 418‐fold selectivity towards the desired isoform.^[^ ^38^ ^]^ Analogs with large bulky groups, such as adamantyl amine **8b** and sulfonamides **8c** and **8d**, displayed affinity for neither isoform. These data suggest a limit to which sterically hindered groups are tolerated at this region of the Hsp90*β* binding pocket, even if such modifications might benefit other properties like solubility. Furthermore, the data from these compounds reinforce our hypothesis that the protons of amine‐bearing analogs likely partake in an intramolecular hydrogen bond with the neighboring lactam, thus limiting their rotation.

**Table 1:** Apparent K_D_ values of **KUNB106** and analogs, as determined via the FP assay.

	Entry	Hsp90*α* K_D_ [ µM]	Hsp90*β* K_D_ µM]	Selectivity for Hsp90*β*
Amine and sulfonamide derivatives	**8a (KUNB106)**	38.00 ± 1.10	0.091 ± 0.010	418‐fold
	**8b**	>100	>100	Not significant
	**8c**	>100	>100	Not significant
	**8d**	>100	>100	Not significant
	**8e**	>100	0.744 ± 0.100	>134‐fold
	**8f**	>100	0.320 ± 0.006	>313‐fold
	**8g**	>100	0.855 ± 0.085	>117‐fold
	**8h**	>100	0.305 ± 0.013	>328‐fold
	**8i**	>100	0.330 ± 0.010	>303‐fold
	**8j**	9.28 ± 0.200	0.049 ± 0.002	189‐fold
Cycloalkyl derivatives	**13a**	>100	4.97 ± 0.53	>20‐fold
	**13b**	>100	0.470 ± 0.041	>213‐fold
	**13c**	>100	0.181 ± 0.019	>552‐fold
	**13d**	>100	0.195 ± 0.008	>513‐fold
	**13e**	>100	0.092 ± 0.002	>1,087‐fold
	**13f**	2.96 ± 0.04	0.044 ± 0.001	67‐fold
	**13g**	>100	>100	Not Significant
	**13h**	>100	0.425 ± 0.009	>235‐fold
	**13i**	>100	0.231 ± 0.020	>433‐fold
	**13j**	2.36 ± 0.09	0.023 ± 0.002	103‐fold

However, as noted in our previous study, this issue can be circumvented via the incorporation of ionizable secondary amines as the solvent‐exposed appendage.^[^ ^38^ ^]^ From those efforts, compounds with oxygen‐containing heterocycles, such as morpholine and piperidinol, were found to bind most potently, which we theorize is due to their participation in hydrogen‐bonding interactions with the ‘gate‐way’ residue, Lys‐58. Consequently, select derivatives of these moieties that alter their electronic properties were also evaluated. Trifluoromethylated enantiomers, **8e** and **8f**, were proposed to promote more favorable interactions with Lys‐58 by inductively increasing electron density towards this residue. Although the *R* isomer (**8f**) exhibited higher affinity and selectivity towards Hsp90*β*, neither improved these properties over the original morpholine analog. Additional derivatives were also assessed to further probe the extent to which steric bulk can be accommodated at this region of the binding pocket. Bicyclic compound **8g** was found to bind Hsp90*β* with an apparent K_D_ = 855 nM, which corresponds to >117‐fold selectivity, while spirocyclic compounds, **8h** and **8i**, exhibited comparable affinities of ≈300 nM (≈300‐fold selectivity). Most notably, bridged morpholine derivative, **8j**, reduced the apparent K_D_'s against Hsp90*α* and Hsp90*β* to 9.28 µM and 0.049 µM, respectively, which corresponds to 189‐fold selectivity towards Hsp90*β*.

Interestingly, **KUNB106** analogs with cycloalkyl rings failed to bind Hsp90*α* but manifested improved affinity and selectivity towards Hsp90*β* with increasing ring size. Cyclopropyl derivative **13a** exhibited an apparent Hsp90*β* K_D_ of 4.97 μM (≈20‐fold selectivity), while that of cyclobutyl‐containing **13b** was ≈10‐fold greater (0.470 μM, >200‐fold selectivity). Inclusion of either a cyclopentyl (**13c**) or cyclohexyl (**13d**) ring resulted in compounds with IC_50_ values of ≈200 nM (>500‐fold selectivity), while cycloheptane‐bearing **13e** was found to bind Hsp90*β* with an affinity of 92 nM (>1,000‐fold selectivity). Incorporation of an oxygen atom into the ring, as exemplified by tetrahydropyran derivative **13f**, displayed improved potency over **13d** (K_D_ = 44 nM), yet only a ≈70‐fold degree of selectivity.

When the cyclohexane and tetrahydropyran rings are connected to the **KUNB106** scaffold via a methylene linker, this extension respectively eliminated binding for **13g** and improved the selectivity of **13h** towards Hsp90*β* to >235‐fold, as compared to its directly attached counterpart, **13f**. Altogether, these data refine our understanding of the extent to which large, bulky groups are tolerated within this region of the Hsp90*β* binding pocket, as well as the importance of handles with heteroatoms to engage with Lys‐58. In our concurrent study on the SAR of the **KUNB106** ‘northern’ fragment, it was revealed that homologation of the pyrazole methyl to an ethyl could also improve Hsp90*β* selectivity at the cost of affinity towards both isoforms.^[^ ^41^ ^]^ Consequently, this observation led to the preparation and evaluation of ethyl‐bearing compounds, **13i** and **13j**. The affinity of **13i** towards Hsp90*β* (K_d_ = 231 nM) was comparable to that of **13d** (K_d_ = 195 nM). However, relative to **13f**, **13j** improved affinity to 23 nM and the selectivity to ≈100‐fold.

### 3.3 Evaluation of Aqueous Solubility

As described earlier in this work, aqueous solubility has been a major limitation of our Hsp90*β*‐selective inhibitors, most likely due to a combination of 1) the molecule's numerous aromatic rings, which contribute to intermolecular *π*‐stacking interactions and the generation of a high crystal lattice energy, and 2) an intramolecular hydrogen bonding interaction that effectively turns the “southern” region of the molecule into a tricyclic aromatic system. Consequently, the solubility of select compounds was measured to determine the impact of the modifications explored herein on this property (**Table** **2**). The solubility of **KUNB106** has previously been established to be 0.014 μM.^[^ ^35^ ^]^ Contrary to our initial hypothesis, analog **8b**, which incorporates a bulky adamantyl group, display sub‐nanomolar solubility at pH 6.8. However, bridged morpholine‐bearing **8j** increased this value to 1.62 µM, which validates this approach to improve these compounds. Although the cyclohexane ring of **13d** manifested poorer solubility than the adamantyl derivative, a single oxygen atom (**13f**) was sufficient to increase this value to 1.54 µM. For these latter cases (**13d** and **13f)**, extension of the original pyrazole methyl to an ethyl group (**13i** and **13j**, respectively) also afforded enhancements in solubility, which is consistent with our complementary study.^[^ ^41^ ^]^

**Table 2:** Aqueous solubility of select **KUNB106** analogs.

Entry	Aqueous solubility, pH 6.8 [ µM]	Fold improvement
**8a (KUNB106)**	0.014	–
**8b**	<0.0005	–
**8j**	1.62	116
**13d**	<0.00002	–
**13f**	1.54	110
**13i**	<0.001	–
**13j**	1.66	119

### 3.4 In Vitro Evaluation of KUNB106 Analogs

In recent studies, Hsp90*β*‐selective inhibitors manifested low micro/nanomolar efficacy against several cancer cell lines in vitro.^[^ ^38^ ^,^ ^41^ ^]^ Therefore, this series of **KUNB106** derivatives were assessed in an MTS antiproliferative assay against the A549, MDA‐MB‐231, and SKOV‐3 cell lines to determine the impact of their modifications on human cancers (**Table** **3**). The IC_50_ values of **KUNB106** against these cell lines were determined to be 7.38 ± 0.24, 3.82 ± 0.05, and 2.98 ± 0.19 μM against these cell lines, respectively.^[^ ^41^ ^]^

**Table 3:** Antiproliferative IC_50_ values of **KUNB106** derivatives against multiple human cancer cell lines, as determined via an MTS cell viability assay.

Entry	IC_50_ [ µM]		
	A549	MDA‐MB‐231	SKOV‐3
**8a (KUNB106)**	7.38 ± 0.24	3.82 ± 0.05	2.98 ± 0.19
**8b**	47.17 ± 2.16	35.72 ± 2.29	>50
**8c**	9.33 ± 0.52	8.51 ± 0.64	9.34 ± 0.56
**8d**	18.49 ± 1.21	8.91 ± 0.81	12.00 ± 0.88
**8e**	8.04 ± 0.06	4.59 ± 0.38	21.86 ± 1.18
**8f**	3.25 ± 0.18	1.62 ± 0.15	2.24 ± 0.22
**8g**	4.41 ± 0.23	7.00 ± 0.23	17.09 ± 1.73
**8h**	4.23 ± 0.12	5.22 ± 0.05	16.50 ± 0.41
**8i**	4.31 ± 0.20	2.19 ± 0.17	11.84 ± 0.61
**8j**	2.22 ± 0.07	0.471 ± 0.015	1.87 ± 0.11
**13a**	1.14 ± 0.09	0.399 ± 0.005	0.592 ± 0.024
**13b**	8.94 ± 0.38	2.55 ± 0.08	2.84 ± 0.18
**13c**	3.79 ± 0.32	0.926 ± 0.050	1.11 ± 0.01
**13d**	11.40 ± 1.10	3.32 ± 0.19	12.24 ± 1.14
**13e**	4.06 ± 0.09	5.61 ± 0.21	7.71 ± 0.15
**13f**	0.368 ± 0.019	0.286 ± 0.024	0.271 ± 0.007
**13g**	15.59 ± 0.84	>50	>50
**13h**	4.91 ± 0.43	1.71 ± 0.04	2.95 ± 0.18
**13i**	26.26 ± 1.94	>50	45.35 ± 1.53
**13j**	0.604 ± 0.053	0.050 ± 0.002	0.185 ± 0.017

According to the FP assay, most of these inhibitors are highly selective (>100‐fold) towards Hsp90*β* and bind the protein with apparent K_D_ values in the sub‐micromolar or nanomolar range. In the MTS assay, such selectivity/affinity generally corresponds to low micromolar antiproliferative IC_50_ values across all three cell lines. In fact, trends observed in the former assay were revealed to carry over to the latter. For instance, *R* enantiomer **8f** was both the more potent Hsp90*β*‐selective inhibitor and the more active species in cellulo, as compared to **8e**. Meanwhile, spirocyclic species **8h** and **8i** exhibited comparable apparent K_D_ and antiproliferative IC_50_ values. Conversely, compounds which failed to bind either cytosolic Hsp90 isoform, such as **8b** and **13g**, likewise manifested minimal cellular activity.

Unfortunately, the improved affinity/selectivity towards Hsp90*β* observed with the increased ring size of cycloalkyl derivatives **13a–e** did not translate into similar in cellulo results. Morpholine **8j** and tetrahydropyran analogs **13f** and **13j** displayed some of the lowest antiproliferative values within this series. Although these analogs are >60‐fold selective towards Hsp90*β* and possess apparent K_D_'s of ≈20–50 nM, it is cautioned that such activity might be attributed to their ability to also bind Hsp90*α* (K_D_’*s* = 2.4–9.3 μM).

Curiously, several **KUNB106** derivatives displayed activity despite poor affinity/selectivity towards Hsp90*β*. The antiproliferative IC_50_ values of sulfonamides **8c** and **8d** were ≈10–20 μM across the three cell lines examined in this study. However, those of cyclopropyl‐containing **13a** were in the nanomolar range, which suggests off‐target activity. It is also noted that the triple‐negative breast cancer MDA‐MB‐231 cell line appears most sensitive to our Hsp90*β*‐selective inhibitors, though any precise mechanism(s) behind this phenomenon are currently unknown and outside the scope of this preliminary study.

**Figure 2:**
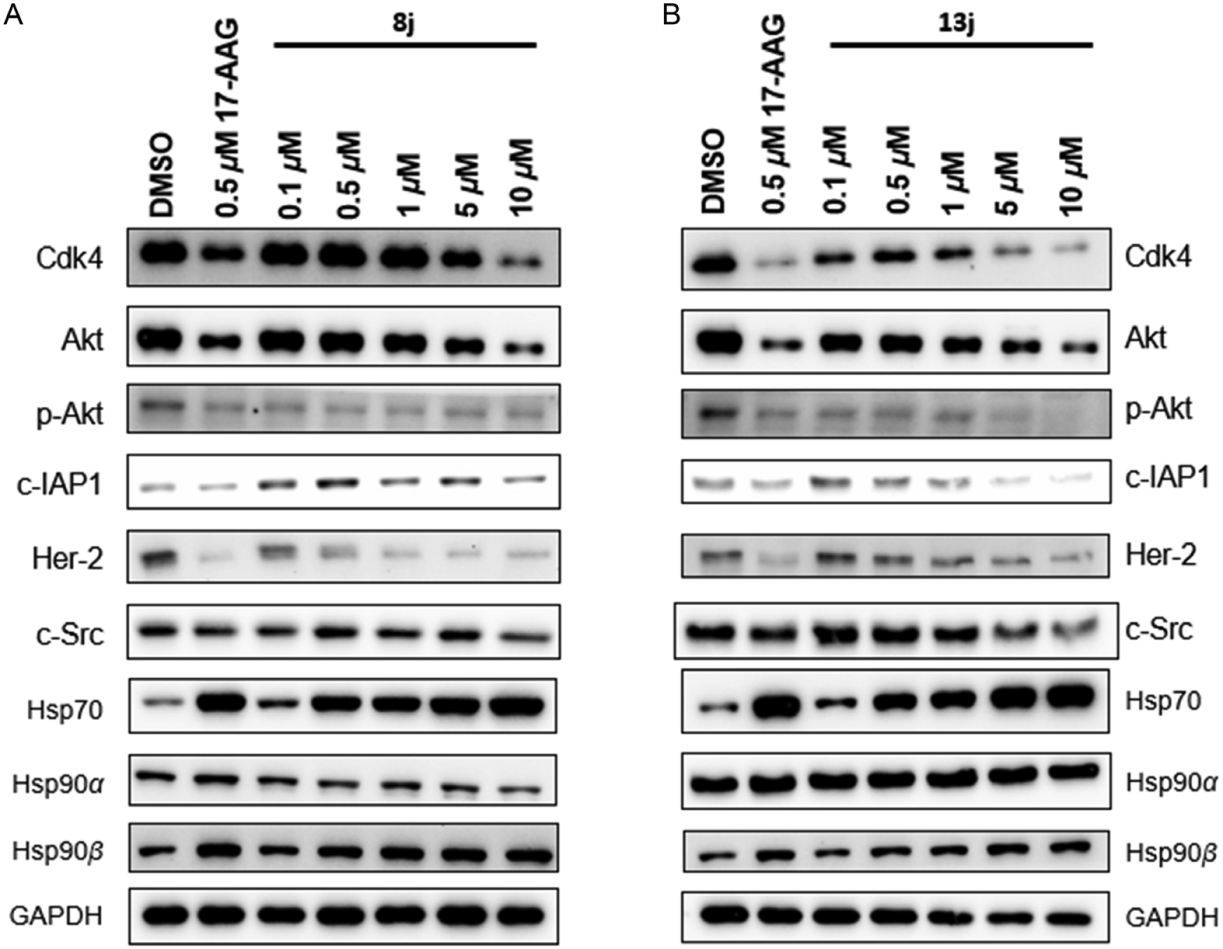
Western blot analysis of Hsp90 client substrates and heat shock proteins with A) **8j** and B) **13j** in MDA‐MB‐231 cells. 17‐AAG (0.5 μM) was included as the positive control, and the vehicle (DMSO) was included as the negative control.

Hsp90 inhibition promotes the degradation of client protein substrates via the ubiquitin‐proteasome pathway. Consequently, **8j** and **13j** were selected to assess their impact on Hsp90 clients and heat shock proteins via Western blot analysis of MDA‐MB‐231 cells (**Figure** **2**A,B). After a 24 h incubation period, both compounds were revealed to facilitate the degradation of the known Hsp90*β*‐dependent clients Cdk4, Akt, p‐Akt, and c‐IAP1 in a dose‐dependent manner, which is consistent with earlier Hsp90*β*‐selective inhibitors.^[^ ^14^ ^,^ ^15^ ^,^ ^38^ ^,^ ^41^ ^]^ Similar results were obtained with regards to Her‐2 levels, though as we have previously indicated, the biological activation of this protein is dependent upon both cytosolic Hsp90 isoforms.^[^ ^38^ ^]^ c‐Src is a known Hsp90*α*‐dependent client;^[^ ^45^ ^,^ ^46^ ^]^ however, levels of this species are affected at higher doses of both compounds. Although both **8j** and **13j** are >100‐fold selective towards Hsp90*β*, their apparent K_D_ values against Hsp90*α* were determined to be 9.28 and 2.36 μM, respectively, which likely accounts for this observation. Altogether, these data validate the isoform‐selectivity of **8j** and **13j** within the cellular context. However, given that the apparent Hsp90*β* K_D_ values of **8j** and **13j** are 49 and 23 nM, respectively, lower doses of these inhibitors will be necessary to bypass detrimental effects on c‐Src and/or other Hsp90*α*‐dependent clients.

Induction of Hsp70 levels is a traditional hallmark of the heat‐shock response and is noted to occur in response to both inhibitors in a dose‐dependent manner. Interestingly, levels of Hsp90*α*, the inducible cytosolic Hsp90 isoform, remain unaffected by both compounds. Such activity has been observed in several recent reports from our lab that include our complementary SAR study, as well as our evaluation on the safety profile of Hsp90*β*‐selective inhibitors, in which one such compound was administered to both MDA‐MB‐231 and MDA‐MB‐468 triple‐negative breast cancer cells.^[^ ^17^ ^,^ ^41^ ^]^ Furthermore, other reports demonstrate that similar compounds manifest low activity against several normal, nontransformed human cell lines (ex: ARPE‐19 and MCF10A), as well as in vivo safety and efficacy.^[^ ^17^ ^,^ ^35^ ^,^ ^47^ ^,^ ^48^ ^]^ Overall, this indicates that **8j** and **13j** can avoid the dose‐escalating toxicities associated with Hsp90 *pan*‐inhibitors and further validates the therapeutic advantages of Hsp90*β*‐selective inhibition.

## 4 Conclusion

Due to its pivotal role in maintaining the proper conformations of >400 client protein substrates, Hsp90 is implicated in a host of biological processes and disease states. Consequently, modulation of the molecular chaperone via small molecule inhibitors is a promising strategy with various therapeutic applications. Although Hsp90 *pan*‐inhibitors have experienced disappointing clinical outcomes, the recent development of Hsp90 isoform‐selective inhibitors represents an exciting paradigm shift. For instance, it has been proven that even a two amino acid difference can be exploited to produce highly potent Hsp90*β*‐selective inhibitors. In a recent report that describes the structure‐guided design of an isoquinolin‐1‐(2*H*)‐one‐containing scaffold, we demonstrated that even subtle changes to the “southern,” solvent‐exposed appendage can drastically modulate affinity and selectivity towards Hsp90*β*.

In this work, nineteen analogs of the Hsp90*β*‐selective inhibitor **KUNB106** were synthesized and evaluated to further elucidate SAR at this region of the molecule. Notably, this study featured numerous derivatives with large/3D amines or sulfonamides, as well as several species that are connected via C—C bonds. Altogether, the data support earlier observations that minor perturbations within this region of the molecule can manifest significant effects on both affinity and selectivity. Results from the FP assay indicate a limit to which steric bulk is tolerated, yet small, bicyclic structures (such as bridged morpholine **8j**) appear to be optimal. These data also reinforce the importance of a heteroatom, as several oxygen‐containing species displayed high affinity and selectivity towards Hsp90*β*. In particular, **8j** and tetrahydropyran **13f** significantly increased affinity towards both cytosolic isoforms, although this comes at the cost of reduced selectivity. Nevertheless, selectivity can be perturbed via extension of the “northern” indazolone methyl to an ethyl group (**13j**), as has been demonstrated elsewhere. Moreover, these modifications were found to enhance the aqueous solubility of **KUNB106** by >100‐fold, which is encouraging for the continued optimization of drug‐like properties. Results from the MTS assay reveal these compounds to manifest antiproliferative activity against several cancer cell lines with micromolar IC_50_ values, though **8j**, **13f**, and **13j** achieved this at nanomolar concentrations. Subsequent evaluation of **8j** and **13j** via Western blot analysis in MDA‐MB‐231 cells validated selective Hsp90*β* inhibition in cellulo, as numerous Hsp90*β*‐dependent clients underwent degradation in a dose‐dependent manner. However, the known Hsp90*α*‐dependent client c‐Src is also impacted at higher concentrations and confirms these compounds’ relatively high affinity for Hsp90*α*. Nevertheless, the failure of these compounds to induce Hsp90*α* levels in cellulo is a positive indication of the therapeutic advantages of Hsp90*β*‐selective inhibition, and evidence that increased levels of Hsp90 will not complicate subsequent studies.

## Abbreviations

**Table:** 

17‐AAG	17‐allylaminogeldanamycin
Akt	protein kinase B
AMPK	AMP‐activated protein kinase
ATP	adenosine triphosphate
Cdk4	cyclin‐dependent kinase 4
c‐IAP1	cellular inhibitor of apoptosis 1
CNS	central nervous system
DCM	dichloromethane
DIPEA	N,N‐diisopropylethylamine
DMAP	N,N‐dimethylaminopyridine
ERK	extracellular signal‐regulated kinase
GAPDH	glyceraldehyde=3‐phosphate dehydrogenase
Grp94	94‐kDa glucose‐regulated protein
Hsp	heat shock protein
Hsp70	heat shock protein 70
Hsp90	heat shock protein 90
IL‐1*β*	interleukin‐1 beta
LPS	lipopolysaccharides
MAPK	mitogen‐activated kinase
MMP3	matrix metalloproteinase 3
NF‐*κ*B	nuclear factor kappa‐light‐chain‐enhancer of activated B cells
NO	nitric oxide
RSK	ribosomal s6 kinase
SAR	structure‐activity relationship
S*N*Ar	nucleophilic aromatic substitution
TNF‐*α*	tumor necrosis factor α
TRAP1	Tumor necrosis receptor‐associated protein 1

## Conflict of Interest

The authors declare the following competing financial interest(s): B.S.J.B. has recently started Grannus Therapeutics to move this research towards clinical development. The authors have founded Grannus Therapeutics to develop a similar scaffold. B.S.J.B., T.D., and M.A.S. are listed as coinventors on a patent pending to the University of Notre Dame related to this work. J.M.S. is an equity holder in Botanical Results, LLC and Teleport Pharmaceuticals, LLC, as well as a consultant for Napreva.

## Author Contributions

**Terin D’Amico** and **Michael A. Serwetnyk** wrote the manuscript. **Terin D’Amico**, **Michael A. Serwetnyk,** and **Xiaozheng Dou** performed the synthesis, purification, characterization, and FP assay evaluation of all compounds featured in this manuscript. **Terin D’Amico** and **Michael A. Serwetnyk** also evaluated all compounds via the MTS assay. **Ian Mersich** performed the Western blot analyses of select compounds. **Deborah Barlow** and **Karen L. Houseknecht** performed solubility studies on select compounds. **Karen L. Houseknecht**, **Brian S. J. Blagg**, and **John M. Streicher** were awarded the funding that supported the work featured in this manuscript. **Aktar Ali** oversaw **Ian Mersich** in the biological evaluation of select compounds. **Brian S. J. Blagg** oversaw and trained **Terin D’Amico**, **Michael A. Serwetnyk** and **Xiaozheng Dou** in the design, synthesis, purification, and characterization of all compounds. **Brian S. J. Blagg** also revised the manuscript. **Terin D’Amico** and **Michael A. Serwetnyk** are co‐first authors.

## Supporting information

Supplementary Material

## Data Availability

The data that support the findings of this study are available from the corresponding author upon reasonable request.
