# Supplementary material for: Hsp90β‐Selective Inhibitors: Probing the Solvent‐Accessible Frontier
Source: ChemMedChem. 2025 Nov 18;20(24):e202500657. doi: 10.1002/cmdc.202500657 (PMC12711159; doi:10.1002/cmdc.202500657)
Supplement: Supplementary file 1 — Supplementary Material [file CMDC-20-e202500657-s001.pdf]

Supporting Information

Hsp90*β*-selective Inhibitors: Probing the Solvent-accessible Frontier

Terin D'Amico[a,‡], Michael A. Serwetnyk[a,‡], Xiaozheng Dou[a], Ian Mersich[a,b], Deborah Barlow[c], Karen L. Houseknecht[c], John M. Streicher[d], Aktar Ali[b], and Brian S. J. Blagg[a,*]

[a] Department of Chemistry and Biochemistry, University of Notre Dame, Notre Dame, Indiana 46556, United States of America

[b] Warren Center for Drug Discovery, University of Notre Dame, Notre Dame, Indiana 46556. United States of America

[c] Department of Biomedical Sciences, College of Osteopathic Medicine, Portland Laboratory for Biotechnology and Health Sciences, University of New England, Portland, Maine 04103, United States of America

[d] Department of Pharmacology, College of Medicine, University of Arizona, Tucson, Arizona 85724, United States of America

‡ - Indicates co-first authors

*Corresponding author: bblagg@nd.edu

Table of Contents

1. Characterization of Intermediates and Compounds – 3–13

2. NMR Spectra of All New Intermediates and Compounds – 14–35

3. Characterization, NMR Spectra, and Biological Evaluation of Select 2*H*-Indazolone Addition Products – 36–42

4. Supplemental References – 42

1. Characterization of Intermediates and Compounds

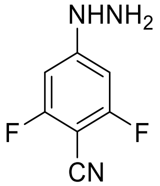

*2,6-difluoro-4-hydrazineylbenzonitrile (**4**).* Yield 65%, 6.98 g. Characterization of **4** is consistent with a prior report.[1]

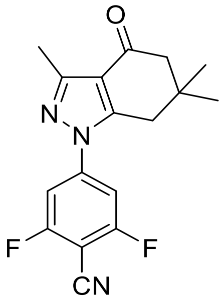

*2,6-difluoro-4-(3,6,6-trimethyl-4-oxo-4,5,6,7-tetrahydro-1H-indazol-1-yl)benzonitrile (**5**).* Yield 84%, 780 mg. Characterization of **5** is consistent with prior reports.[1,2]

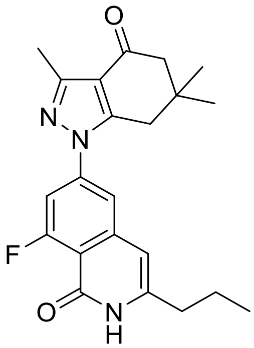

*8-fluoro-3-propyl-6-(3,6,6-trimethyl-4-oxo-4,5,6,7-tetrahydro-1H-indazol-1-yl)isoquinolin-1(2H)-on (**7**).* Yield 28%, 185 mg. Characterization of **7** is consistent with prior reports.[1,2]

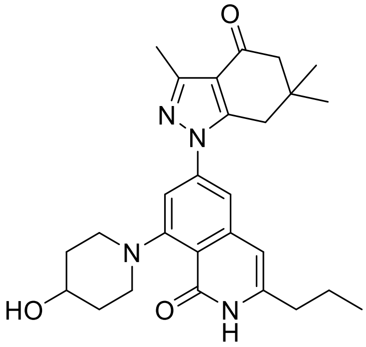

*8-(4-hydroxypiperidin-1-yl)-3-propyl-6-(3,6,6-trimethyl-4-oxo-4,5,6,7-tetrahydro-1H-indazol-1-yl)isoquinolin-1(2H)-one (**KUNB106**, **8a**)*. Yield 47%, 17 mg. Characterization of **KUNB106** is consistent with prior reports.[1,2]

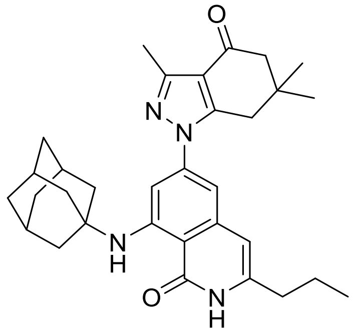

*8-(((1s,3s)-adamantan-1-yl)amino)-3-propyl-6-(3,6,6-trimethyl-4-oxo-4,5,6,7-tetrahydro-1H-indazol-1-yl)isoquinolin-1(2H)-one (**8b**)*. Yield 46 %, 20 mg; ^{1}H NMR (400 MHz, Chloroform-*d*) δ 10.47 (s, 1H), 9.60 (s, 1H), 6.74 (d, *J* = 2.0 Hz, 1H), 6.68 (d, *J* = 1.8 Hz, 1H), 6.18 (d, *J* = 1.8 Hz, 1H), 2.91 (s, 2H), 2.55 (d, *J* = 11.8 Hz, 5H), 2.42 (s, 2H), 2.18 (t, *J* = 3.3 Hz, 3H), 2.14 (d, *J* = 2.8 Hz, 6H), 1.84–1.67 (m, 8H), 1.12 (s, 6H), 0.99 (t, *J* = 7.3 Hz, 3H). ^{13}C NMR (101 MHz, $CDCl_3$) δ 193.49, 166.37, 150.86, 149.95, 148.85, 142.48, 142.25, 141.68, 117.22, 108.09, 105.50, 104.71, 102.49, 52.41, 51.70, 42.11 (2), 38.34, 36.58 (2), 35.84, 34.63, 29.55 (2), 28.44 (2), 21.20, 13.53, 13.43. HRMS (ESI/Q-TOF) m/z: [M + H]+ Calcd for $C_{32}H_{41}N_4O_2$ 513.3224; Found 513.3213.

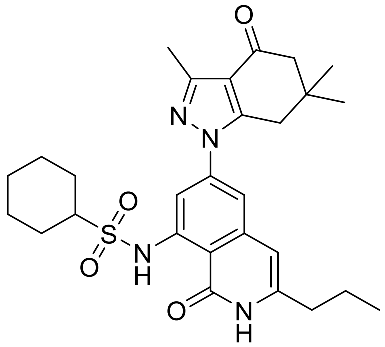

*N-(1-oxo-3-propyl-6-(3,6,6-trimethyl-4-oxo-4,5,6,7-tetrahydro-1H-indazol-1-yl)-1,2-dihydroisoquinolin-8-yl)cyclohexanesulfonamide (**8c**)*. Yield 20%, 5.6 mg; ^{1}H NMR (400 MHz, Chloroform-*d*) δ 12.30 (s, 1H), 10.96 (s, 1H), 7.76 (d, *J* = 2.0 Hz, 1H), 7.41 (d, *J* = 2.0 Hz, 1H), 6.43 (d, *J* = 1.8 Hz, 1H), 3.11 (tt, *J* = 12.2, 3.6 Hz, 1H), 2.91 (s, 2H), 2.62 (t, *J* = 7.4 Hz, 2H), 2.57 (s, 3H), 2.43 (s, 2H), 2.22 (d, *J* = 12.6 Hz, 2H), 1.89 (d, *J* = 11.0 Hz, 2H), 1.80 (p, *J* = 7.4 Hz, 2H), 1.70 – 1.62 (m, 2H), 1.24 (q, *J* = 11.2, 9.3 Hz, 4H), 1.12 (s, 6H), 1.04 (t, *J* = 7.3 Hz, 3H). ^{13}C NMR (101 MHz, $CDCl_3$) δ 193.53, 165.83, 150.72, 149.54, 143.04, 142.76, 142.71, 141.69, 117.84, 113.20, 109.32, 106.46, 106.02, 60.92, 52.29, 37.78, 35.95, 35.02, 28.46 (2), 26.23 (2), 25.11 (2), 25.01, 21.41, 13.50, 13.38. HRMS (ESI/Q-TOF) m/z: [M + H]+ Calcd for $C_{28}H_{37}N_4O_4S$ 525.253; Found 525.252.

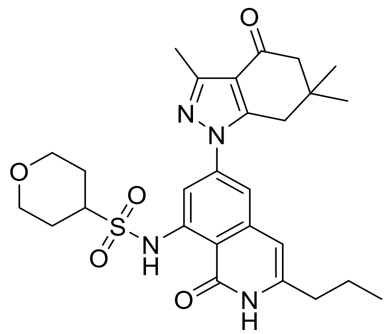

*N-(1-oxo-3-propyl-6-(3,6,6-trimethyl-4-oxo-4,5,6,7-tetrahydro-1H-indazol-1-yl)-1,2-dihydroisoquinolin-8-yl)cyclohexanesulfonamide (**8d**).* Yield 22%, 6.2 mg; ^{1}H NMR (400 MHz, Chloroform-*d*) δ 12.41 (s, 1H), 11.06 (s, 1H), 7.79 (d, *J* = 2.0 Hz, 1H), 7.41 (d, *J* = 2.0 Hz, 1H), 6.45 (d, *J* = 1.7 Hz, 1H), 4.07 (dt, *J* = 11.7, 3.4 Hz, 2H), 3.35 (qd, *J* = 11.3, 10.7, 4.4 Hz, 3H), 2.91 (s, 2H), 2.63 (t, *J* = 7.4 Hz, 2H), 2.57 (s, 3H), 2.43 (s, 2H), 2.02 (td, *J* = 11.4, 10.2, 4.3 Hz, 4H), 1.81 (h, *J* = 7.4 Hz, 2H), 1.12 (s, 6H), 1.05 (t, *J* = 7.3 Hz, 3H). ^{13}C NMR (101 MHz, $CDCl_3$) δ 193.48, 165.82, 150.81, 149.52, 143.17, 142.78, 142.46, 141.70, 117.89, 113.48, 109.38, 106.74, 106.08, 66.43 (2), 58.12, 52.27, 37.78, 35.96, 35.04, 28.47 (2), 26.29 (2), 21.44, 13.50, 13.41. HRMS (ESI/Q-TOF) m/z: [M + H]+ Calcd for $C_{27}H_{35}N_4O_5S$ 527.2323; Found 527.2326.

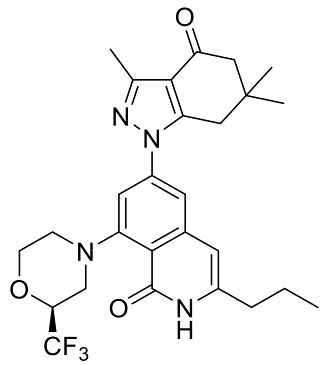

*(S)-3-propyl-8-(2-(trifluoromethyl)morpholino)-6-(3,6,6-trimethyl-4-oxo-4,5,6,7-tetrahydro-1H-indazol-1-yl)isoquinolin-1(2H)-one (**8e**).* Yield 51%, 21 mg; ^{1}H NMR (400 MHz, Chloroform-*d*) δ 10.08 (s, 1H), 7.14 – 7.06 (m, 2H), 6.28 (s, 1H), 4.43 (dtt, *J* = 8.7, 6.4, 3.9 Hz, 1H), 4.19 – 4.04 (m, 2H), 3.73 – 3.64 (m, 1H), 3.41 (dd, *J* = 11.5, 2.2 Hz, 1H), 3.10 (td, *J* = 11.4, 3.4 Hz, 1H), 2.89 (d, *J* = 16.3 Hz, 3H), 2.58 (d, *J* = 9.7 Hz, 5H), 2.43 (s, 2H), 1.77 (h, *J* = 7.4 Hz, 3H), 1.12 (d, *J* = 4.3 Hz, 5H), 1.03 (t, *J* = 7.4 Hz, 3H). ^{13}C NMR (101 MHz, $CDCl_3$) δ 193.43, 162.39, 154.52, 150.54, 149.21, 143.27, 143.22, 141.95, 124.78, 122.00, 117.58, 115.60, 113.50, 110.25, 104.36, 73.93 (q, *J* = 31.2 Hz), 67.17, 52.34, 51.92, 51.82, 37.66, 35.94, 34.79, 28.55, 28.35, 21.11, 13.50, 13.47. ^{19}F NMR (376 MHz, $CDCl_3$) δ -77.44. HRMS (ESI) m/z [M + H] calc'd for $C_{27}H_{32}F_3N_4O_3$, 517.2421, found 517.2426.

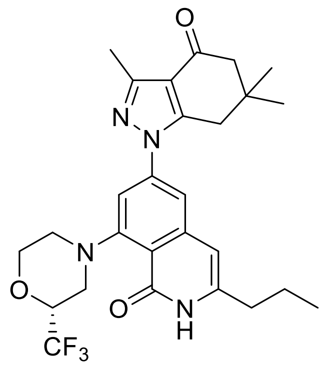

*(R)-3-propyl-8-(2-(trifluoromethyl)morpholino)-6-(3,6,6-trimethyl-4-oxo-4,5,6,7-tetrahydro-1H-indazol-1-yl)isoquinolin-1(2H)-one (**8f**)*. Yield 86%, 35mg; ^{1}H NMR (400 MHz, Chloroform-*d*) δ 10.41 (s, 1H), 7.15 – 7.07 (m, 2H), 6.29 (s, 1H), 4.42 (ddt, *J* = 10.5, 6.5, 3.2 Hz, 1H), 4.19 – 4.04 (m, 2H), 3.73 – 3.62 (m, 1H), 3.45 – 3.38 (m, 1H), 3.10 (td, *J* = 11.4, 3.3 Hz, 1H), 2.89 (d, *J* = 16.7 Hz, 3H), 2.60 (t, *J* = 7.6 Hz, 2H), 2.56 (s, 3H), 2.42 (s, 2H), 1.78 (h, *J* = 7.4 Hz, 2H), 1.12 (d, *J* = 4.4 Hz, 6H), 1.03 (t, *J* = 7.3 Hz, 3H). ^{13}C NMR (101 MHz, $CDCl_3$) δ 193.44, 162.59, 154.51, 150.52, 149.23, 143.43, 143.25, 141.92, 124.77, 121.99, 117.57, 115.60, 113.51, 110.24, 104.36, 73.94 (q, *J* = 31.2 Hz), 67.17, 52.34, 51.90, 51.83, 37.65, 35.94, 34.75, 28.54, 28.34, 21.12, 13.49, 13.46. ^{19}F NMR (376 MHz, $CDCl_3$) δ -77.42. HRMS (ESI) m/z [M + H] calc'd for $C_{27}H_{32}F_3N_4O_3$, 517.2421, found 517.2427.

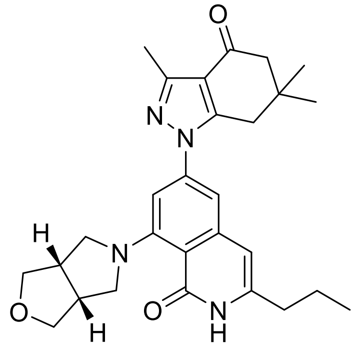

*3-propyl-8-((3aR,6aS)-tetrahydro-1H-furo[3,4-c]pyrrol-5(3H)-yl)-6-(3,6,6-trimethyl-4-oxo-4,5,6,7-tetrahydro-1H-indazol-1-yl)isoquinolin-1(2H)-one (**8g**)*. Yield 85%, 32 mg; ^{1}H NMR (400 MHz, Chloroform-*d*) δ 10.28 (s, 1H), 6.96 (dd, *J* = 22.8, 2.0 Hz, 2H), 6.22 (s, 1H), 3.89 (dd, *J* = 8.8, 5.9 Hz, 2H), 3.82 (dd, *J* = 9.0, 3.2 Hz, 2H), 3.67 – 3.57 (m, 2H), 3.20 (dd, *J* = 9.5, 3.7 Hz, 2H), 3.06 (dd, *J* = 7.4, 4.2 Hz, 2H), 2.84 (s, 2H), 2.56 (d, *J* = 2.7 Hz, 5H), 2.40 (s, 2H), 1.74 (h, *J* = 7.4 Hz, 2H), 1.10 (s, 6H), 1.00 (t, *J* = 7.3 Hz, 3H). ^{13}C NMR (101 MHz, $CDCl_3$) δ 193.51, 162.86, 151.71, 150.21, 149.20, 142.96, 142.94, 141.67, 117.32, 114.59, 111.57, 108.81, 104.23, 72.82 (2), 56.87 (2), 52.40, 43.66 (2), 37.56, 35.91, 34.64, 28.45 (2), 21.13, 13.55, 13.48. HRMS (ESI) m/z [M + H] calc'd for $C_{28}H_{35}N_4O_3$, 475.2704, found 475.2697.

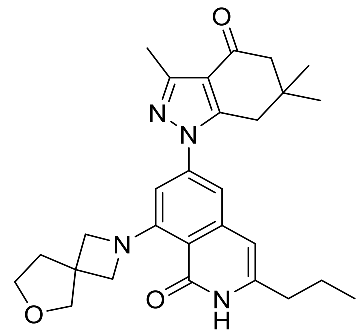

*3-propyl-8-(6-oxa-2-azaspiro[3.4]octan-2-yl)-6-(3,6,6-trimethyl-4-oxo-4,5,6,7-tetrahydro-1H-indazol-1-yl)isoquinolin-1(2H)-one (**8h**).* Yield 45%, 17 mg; ^{1}H NMR (400 MHz, Chloroform-*d*) δ 10.11 (s, 1H), 6.84 (d, *J* = 2.0 Hz, 1H), 6.51 (d, *J* = 2.0 Hz, 1H), 6.19 (d, *J* = 1.8 Hz, 1H), 4.17 (s, 4H), 3.93 (s, 2H), 3.86 (t, *J* = 6.9 Hz, 2H), 2.82 (s, 2H), 2.55 (s, 5H), 2.40 (s, 2H), 2.21 (t, *J* = 6.9 Hz, 2H), 1.77 (h, *J* = 7.4 Hz, 3H), 1.10 (s, 6H), 1.03 (t, *J* = 7.3 Hz, 3H). ^{13}C NMR (101 MHz, $CDCl_3$) δ 193.49, 162.61, 153.61, 150.14, 149.20, 142.55, 141.68, 117.28, 110.89, 108.99, 105.29, 104.16, 67.78 (2), 64.83, 52.42, 40.78, 38.22, 37.59, 35.89, 34.78, 28.44 (2), 21.15, 13.60, 13.48. HRMS (ESI) m/z [M + H] calc'd for $C_{28}H_{35}N_4O_3$, 475.2704, found 475.2698.

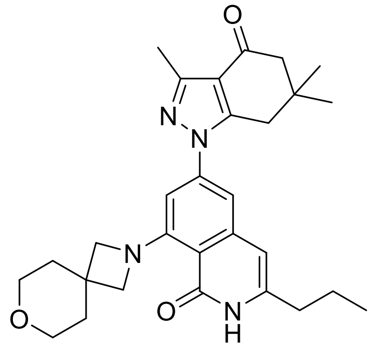

*3-propyl-8-(7-oxa-2-azaspiro[3.5]nonan-2-yl)-6-(3,6,6-trimethyl-4-oxo-4,5,6,7-tetrahydro-1H-indazol-1-yl)isoquinolin-1(2H)-one (**8i**).* Yield 41%, 16 mg; ^{1}H NMR (400 MHz, Chloroform-*d*) δ 10.04 (s, 1H), 6.80 (d, *J* = 2.0 Hz, 1H), 6.49 (d, *J* = 2.0 Hz, 1H), 6.18 (d, *J* = 1.8 Hz, 1H), 3.97 (s, 4H), 3.66 (q, *J* = 5.1 Hz, 4H), 2.82 (s, 2H), 2.55 (d, *J* = 4.5 Hz, 5H), 2.40 (s, 2H), 1.84 (t, *J* = 5.2 Hz, 4H), 1.76 (h, *J* = 7.4 Hz, 3H), 1.10 (s, 6H), 1.02 (t, *J* = 7.3 Hz, 3H). ^{13}C NMR (101 MHz, $CDCl_3$) δ 193.50, 162.83, 153.78, 150.09, 149.23, 142.58, 142.56, 141.65, 127.79, 117.22, 113.91, 110.51, 108.51, 104.98, 104.20, 65.32 (2), 65.05 (2), 52.42, 37.56, 36.53, 35.88, 34.74 (2), 32.69, 28.43 (2), 21.17, 13.61, 13.48. HRMS (ESI) m/z [M + H] calc'd for $C_{29}H_{37}N_4O_3$, 489.2860, found 489.2865.

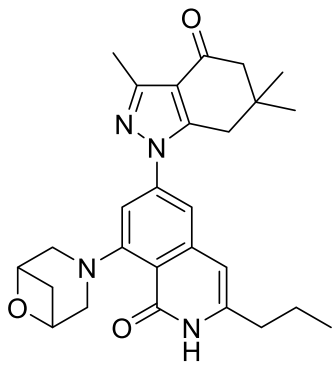

*8-(6-oxa-3-azabicyclo[3.1.1]heptan-3-yl)-3-propyl-6-(3,6,6-trimethyl-4-oxo-4,5,6,7-tetrahydro-1H-indazol-1-yl)isoquinolin-1(2H)-one (**8j**)*. Yield 72%, 26 mg; ^{1}H NMR (400 MHz, Chloroform-*d*) δ 10.44 (s, 1H), 7.16 (d, *J* = 2.0 Hz, 1H), 7.06 (d, *J* = 2.0 Hz, 1H), 6.26 (s, 1H), 4.67 (d, *J* = 6.0 Hz, 2H), 4.00 – 3.91 (m, 2H), 3.63 (d, *J* = 11.5 Hz, 2H), 3.22 (dt, *J* = 7.8, 6.1 Hz, 1H), 2.87 (s, 2H), 2.74 (d, *J* = 7.9 Hz, 1H), 2.55 (d, *J* = 12.2 Hz, 5H), 2.42 (s, 2H), 1.74 (h, *J* = 7.4 Hz, 2H), 1.12 (s, 6H), 1.00 (t, *J* = 7.4 Hz, 3H). ^{13}C NMR (101 MHz, $CDCl_3$) δ 193.46, 163.09, 153.57, 150.27, 149.17, 143.36, 142.89, 141.91, 138.23, 133.08, 123.67, 117.40, 116.85, 114.89, 111.99, 109.77, 104.49, 79.54 (2), 78.22 (2), 53.96, 52.41, 49.56, 37.60, 35.94, 34.38, 32.61, 31.37, 28.48 (2), 21.06, 13.49, 13.45. HRMS (ESI) m/z [M + H] calc'd for $C_{27}H_{33}N_4O_3$, 461.2547, found 461.2546.

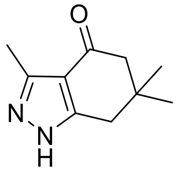

*3,6,6-trimethyl-1,5,6,7-tetrahydro-4H-indazol-4-one (**9a**)*. Yield 85%, 8.3 g; ^{1}H NMR (400 MHz, Chloroform-*d*) δ 7.80 (s, 1H), 2.68 (s, 2H), 2.54 (d, *J* = 1.6 Hz, 3H), 2.34 (d, *J* = 1.5 Hz, 2H), 1.08 (d, *J* = 1.8 Hz, 6H). ^{13}C NMR (101 MHz, $CDCl_3$) δ 194.72, 154.17, 145.00, 114.61, 53.01, 36.35, 35.60, 28.45 (2), 12.06. HRMS (ESI) m/z [M + H] calc'd for $C_{10}H_{15}N_2O$, 179.1179, found 179.1177. Characterization of **9a** is consistent with previous reports.[3]

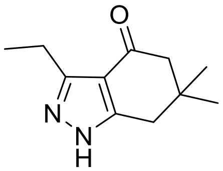

*3-ethyl-6,6-dimethyl-1,5,6,7-tetrahydro-4H-indazol-4-one (**9b**)*. Yield 80%, 7.6 g; ^{1}H NMR (400 MHz, Chloroform-*d*) δ 10.91 (s, 1H), 2.93 (q, *J* = 7.6 Hz, 2H), 2.66 (s, 2H), 2.33 (s, 2H), 1.25 (t, *J* = 7.5 Hz, 3H), 1.06 (s, 6H). ^{13}C NMR (101 MHz, $CDCl_3$) δ 194.47, 154.37, 150.72, 113.85, 53.06, 36.34, 35.51, 28.42 (2), 19.99, 12.80. HRMS (ESI) m/z [M + H] calc'd for $C_{11}H_{17}N_2O$, 193.1335, found 193.1333.

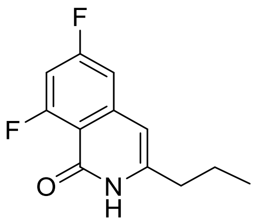

*6,8-difluoro-3-propylisoquinolin-1(2H)-one (**11**).* Yield 20%, 333 mg; Characterization of **11** is consistent with previous studies.[1]

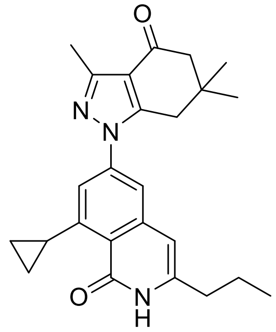

*8-cyclopropyl-3-propyl-6-(3,6,6-trimethyl-4-oxo-4,5,6,7-tetrahydro-1H-indazol-1-yl)isoquinolin-1(2H)-one (**13a**).* Yield 7%, 11 mg; ^{1}H NMR (400 MHz, Chloroform-*d*) δ 11.47 (s, 1H), 7.33 (d, *J* = 2.2 Hz, 1H), 7.10 (d, *J* = 2.1 Hz, 1H), 6.30 (s, 1H), 3.67 (td, *J* = 8.7, 4.3 Hz, 1H), 2.84 (s, 2H), 2.60 (t, *J* = 7.6 Hz, 2H), 2.56 (s, 3H), 2.41 (s, 2H), 1.81 (h, *J* = 7.4 Hz, 2H), 1.11 (s, 8H), 1.00 (t, *J* = 7.3 Hz, 3H), 0.87–0.79 (m, 2H). ^{13}C NMR (101 MHz, $CDCl_3$) δ 193.43, 165.10, 150.33, 149.33, 149.13, 143.14, 141.63, 141.15, 122.15, 117.54, 117.39, 117.16, 104.51, 52.40, 37.55, 35.94, 35.12, 28.44 (2), 21.65, 15.09, 13.62, 13.46, 9.83 (2). HRMS (ESI/Q-TOF) m/z: [M + H]+ Calcd for $C_{25}H_{30}N_3O_2$ 404.2333; Found 404.2342. R_f = 0.32 (50% ethyl acetate in hexanes)

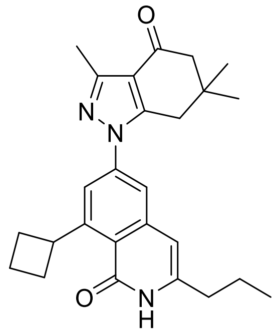

*8-cyclobutyl-3-propyl-6-(3,6,6-trimethyl-4-oxo-4,5,6,7-tetrahydro-1H-indazol-1-yl)isoquinolin-1(2H)-one (**13b**).*Yield 6%, 9 mg; ^{1}H NMR (400 MHz, Chloroform-*d*) δ 11.91 (s, 1H), 7.50 (d, *J* = 2.2 Hz, 1H), 7.37 (d, *J* = 2.2 Hz, 1H), 6.32 (s, 1H), 4.90 (p, *J* = 8.6 Hz, 1H), 2.89 (s, 2H), 2.65 (t, *J* = 7.6 Hz, 2H), 2.61–2.49 (m, 5H), 2.43 (s, 2H), 2.24–2.11 (m, 2H), 2.11–1.98 (m, 1H), 1.87 (dt, *J* = 13.0, 6.7 Hz, 3H), 1.13 (s, 6H), 1.08 (t, *J* = 7.4 Hz, 3H). ^{13}C NMR (101 MHz, $CDCl_3$) δ 193.46, 164.73, 150.38, 150.33, 149.19, 143.37, 141.79, 141.08, 120.64, 119.07, 117.46, 117.40, 104.38, 52.43, 39.90, 37.60, 35.95, 35.14, 29.50 (2), 28.46 (2), 21.73, 18.13, 13.75, 13.49. HRMS (ESI/Q-TOF) m/z: [M + H]+ Calcd for $C_{26}H_{32}N_3O_2$ 418.2489; Found 418.2496. R_f = 0.47 (50% ethyl acetate in hexanes)

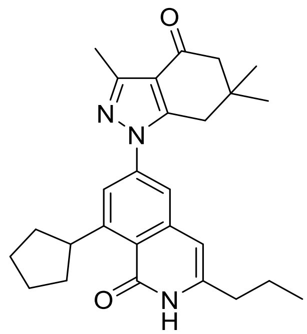

*8-cyclopentyl-3-propyl-6-(3,6,6-trimethyl-4-oxo-4,5,6,7-tetrahydro-1H-indazol-1-yl)isoquinolin-1(2H)-one (**13c**).* Yield 5%, 8 mg; ^{1}H NMR (400 MHz, Chloroform-*d*) δ 11.89 (s, 1H), 7.50 (d, *J* = 2.2 Hz, 1H), 7.39 (d, *J* = 2.2 Hz, 1H), 6.33 (s, 1H), 5.02 – 4.90 (m, 1H), 2.87 (s, 2H), 2.63 (t, *J* = 7.6 Hz, 2H), 2.57 (s, 3H), 2.42 (s, 2H), 2.25 (h, *J* = 5.3, 4.7 Hz, 2H), 1.92–1.72 (m, 6H), 1.66 (dp, *J* = 14.9, 5.7, 3.8 Hz, 2H), 1.12 (s, 6H), 1.03 (t, *J* = 7.3 Hz, 3H). ^{13}C NMR (101 MHz, $CDCl_3$) δ 193.46, 165.17, 152.24, 150.32, 149.14, 143.00, 141.76, 141.04, 121.05, 118.56, 117.52, 117.39, 104.73, 52.42, 42.17, 37.59, 35.93, 34.91, 34.44 (2), 28.45 (2), 25.49 (2), 21.57, 13.63, 13.49. HRMS (ESI/Q-TOF) m/z: [M + H]+ Calcd for $C_{27}H_{34}N_3O_2$ 432.2646; Found 432.2653. R_f = 0.54 (50% ethyl acetate in hexanes)

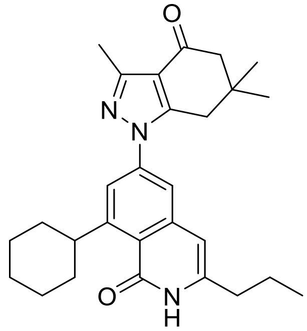

*8-cyclohexyl-3-propyl-6-(3,6,6-trimethyl-4-oxo-4,5,6,7-tetrahydro-1H-indazol-1-yl)isoquinolin-1(2H)-one (**13d**).* Yield 6%, 10 mg; ^{1}H NMR (400 MHz, Chloroform-*d*) δ 11.50 (s, 1H), 7.47 (d, *J* = 2.2 Hz, 1H), 7.40 (d, *J* = 2.1 Hz, 1H), 6.33 (s, 1H), 4.60 (t, *J* = 11.6 Hz, 1H), 2.87 (s, 2H), 2.66 (t, *J* = 7.5 Hz, 2H), 2.58 (s, 3H), 2.43 (s, 2H), 2.06 (d, *J* = 11.9 Hz, 2H), 1.90 (d, *J* = 13.0 Hz, 2H), 1.81 (dt, *J* = 14.4, 7.3 Hz, 3H), 1.65–1.50 (m, 2H), 1.50–1.38 (m, 2H), 1.31 (tdd, *J* = 17.9, 11.4, 4.5 Hz, 1H), 1.13 (s, 6H), 1.03 (t, *J* = 7.3 Hz, 3H). ^{13}C NMR (101 MHz, $CDCl_3$) δ 193.48, 164.96, 153.94, 150.34, 149.16, 142.77, 141.82, 141.11, 118.77, 117.51, 117.40, 104.73, 52.44, 40.03, 37.60, 35.95, 34.76 (2), 34.67, 28.46 (2), 27.29 (2), 26.44, 21.38, 13.51, 13.50. HRMS (ESI/Q-TOF) m/z: [M + H]+ Calcd for $C_{28}H_{36}N_3O_2$ 446.2802; Found 446.2809. R_f = 0.57 (50% ethyl acetate in hexanes)

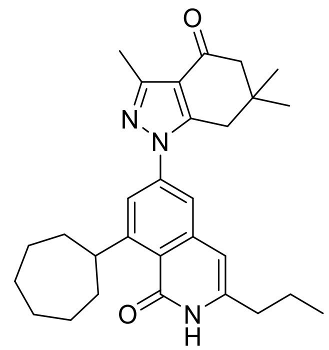

*8-cycloheptyl-3-propyl-6-(3,6,6-trimethyl-4-oxo-4,5,6,7-tetrahydro-1H-indazol-1-yl)isoquinolin-1(2H)-one (**13e**).* Yield 5%, 8 mg; ^{1}H NMR (400 MHz, Chloroform-*d*) δ 10.90 (s, 1H), 7.43 (d, *J* = 2.2 Hz, 1H), 7.38 (d, *J* = 2.2 Hz, 1H), 6.31 (d, *J* = 1.8 Hz, 1H), 4.86 (t, *J* = 5.9 Hz, 1H), 2.87 (s, 2H), 2.63 (t, *J* = 7.4 Hz, 2H), 2.57 (s, 3H), 2.43 (s, 2H), 2.10 (d, *J* = 12.4 Hz, 2H), 1.90–1.73 (m, 6H), 1.64 (d, *J* = 11.7 Hz, 6H), 1.13 (s, 6H), 1.02 (t, *J* = 7.3 Hz, 3H). ^{13}C NMR (101 MHz, $CDCl_3$) δ 193.50, 164.72, 156.22, 150.33, 149.14, 142.42, 141.46, 141.09, 119.87, 119.17, 117.39, 117.23, 104.62, 52.42, 40.97, 37.61 (2), 37.16, 35.94, 34.63, 28.46 (2), 27.94 (2), 27.74 (2), 21.24, 13.50, 13.43. HRMS (ESI/Q-TOF) m/z: [M + H]+ Calcd for $C_{29}H_{38}N_3O_2$ 460.2959; Found 460.2952. R_f = 0.60 (50% ethyl acetate in hexanes)

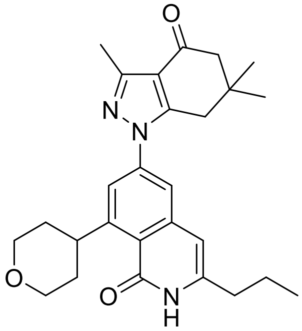

*3-propyl-8-(tetrahydro-2H-pyran-4-yl)-6-(3,6,6-trimethyl-4-oxo-4,5,6,7-tetrahydro-1H-indazol-1-yl)isoquinolin-1(2H)-one (**13f**).* Yield 9%, 14 mg; ^{1}H NMR (400 MHz, Chloroform-*d*) δ 11.09 (s, 1H), 7.53 – 7.41 (m, 2H), 6.35 (d, *J* = 1.8 Hz, 1H), 4.88 (tt, *J* = 11.7, 3.4 Hz, 1H), 4.14 (dd, *J* = 11.1, 4.0 Hz, 2H), 3.69 (td, *J* = 11.6, 2.0 Hz, 2H), 2.88 (s, 2H), 2.64 (t, *J* = 7.4 Hz, 2H), 2.58 (s, 3H), 2.44 (s, 2H), 1.97 (d, *J* = 12.3 Hz, 2H), 1.91 – 1.74 (m, 4H), 1.13 (s, 6H), 1.04 (t, *J* = 7.3 Hz, 3H). ^{13}C NMR (101 MHz, $CDCl_3$) δ 193.44, 164.65, 151.53, 150.51, 149.12, 142.72, 141.91, 141.33, 120.31, 118.77, 117.96, 117.55, 104.80, 68.79 (2), 52.39, 37.64, 37.45, 35.98, 34.70, 34.23 (2), 28.49 (2), 21.33, 13.53, 13.48. HRMS (ESI/Q-TOF) m/z: [M + H]+ Calcd for $C_{29}H_{34}N_3O_3$ 448.2595; Found 448.2602. R_f = 0.15 (50% ethyl acetate in hexanes)

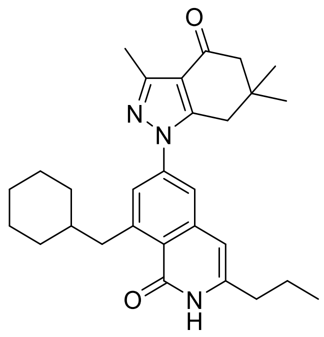

*8-(cyclohexylmethyl)-3-propyl-6-(3,6,6-trimethyl-4-oxo-4,5,6,7-tetrahydro-1H-indazol-1-yl)isoquinolin-1(2H)-one (**13g**).* Yield 6%, 9 mg; ^{1}H NMR (400 MHz, Chloroform-*d*) δ 11.55 (s, 1H), 7.47–7.39 (m, 1H), 7.18 (dd, *J* = 6.7, 2.3 Hz, 1H), 6.35–6.28 (m, 1H), 3.33 (d, *J* = 6.4 Hz, 2H), 2.88 (s, 2H), 2.64 (t, *J* = 7.4 Hz, 2H), 2.57 (s, 3H), 2.42 (s, 2H), 1.89–1.72 (m, 6H), 1.69 (d, *J* = 8.4 Hz, 4H), 1.21–1.16 (m, 3H), 1.12 (s, 6H), 1.03 (t, *J* = 7.4 Hz, 3H). ^{13}C NMR (101 MHz, $CDCl_3$) δ 193.47, 164.88, 150.35, 149.21, 146.78, 143.01, 141.91, 140.31, 123.73, 121.26, 117.94, 117.39, 104.47, 52.40, 43.44, 39.22, 37.67, 35.93 (2), 34.71, 33.39 (2), 28.55, 28.46 (2), 26.69, 26.37, 21.42, 13.49. HRMS (ESI/Q-TOF) m/z: [M + H]+ Calcd for $C_{29}H_{38}N_3O_2$ 460.2959; Found 460.2947. R_f = 0.56 (50% ethyl acetate in hexanes)

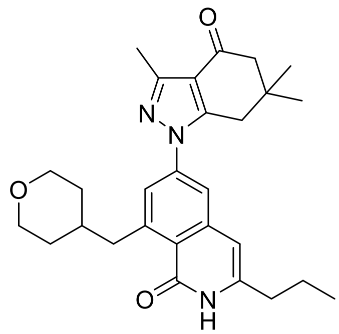

*3-propyl-8-((tetrahydro-2H-pyran-4-yl)methyl)-6-(3,6,6-trimethyl-4-oxo-4,5,6,7-tetrahydro-1H-indazol-1-yl)isoquinolin-1(2H)-one (**13h**).* Yield 5%, 7 mg; ^{1}H NMR (400 MHz, Chloroform-*d*) δ 11.37 (s, 1H), 7.46 (d, *J* = 2.2 Hz, 1H), 7.22 (d, *J* = 2.2 Hz, 1H), 6.34 (d, *J* = 1.7 Hz, 1H), 3.98–3.90 (m, 2H), 3.43–3.27 (m, 4H), 2.88 (s, 2H), 2.62 (t, *J* = 7.4 Hz, 2H), 2.57 (s, 3H), 2.43 (s, 2H), 2.07 (dtp, *J* = 10.7, 6.8, 3.3 Hz, 1H), 1.81 (h, *J* = 7.4, 6.9 Hz, 2H), 1.69–1.58 (m, 2H), 1.47 (qd, *J* = 12.1, 4.5 Hz, 2H), 1.12 (s, 6H), 1.03 (t, *J* = 7.3 Hz, 3H). ^{13}C NMR (101 MHz, $CDCl_3$) δ 193.41, 164.75, 150.48, 149.16, 145.54, 143.00, 141.99, 140.50, 123.80, 121.09, 118.14, 117.51, 104.63, 68.09 (2), 52.35, 43.01, 37.70, 36.58, 35.95, 34.81, 33.22 (2), 28.48 (2), 21.43, 13.55, 13.48. HRMS (ESI/Q-TOF) m/z: [M + H]+ Calcd for $C_{28}H_{36}N_3O_3$ 462.2751; Found 462.2759. R_f = 0.15 (50% ethyl acetate in hexanes)

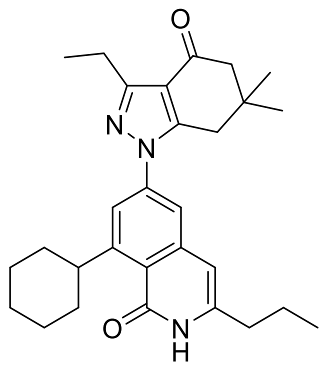

*8-cyclohexyl-6-(3-ethyl-6,6-dimethyl-4-oxo-4,5,6,7-tetrahydro-1H-indazol-1-yl)-3-propylisoquinolin-1(2H)-one (**13i**).* Yield 11%, 18 mg; ^{1}H NMR (400 MHz, Chloroform-*d*) δ 11.36 (s, 1H), 7.49–7.36 (m, 2H), 6.33 (d, *J* = 1.8 Hz, 1H), 4.60 (tt, *J* = 11.4, 3.0 Hz, 1H), 2.99 (q, *J* = 7.5 Hz, 2H), 2.87 (s, 2H), 2.65 (t, *J* = 7.4 Hz, 2H), 2.43 (s, 2H), 2.11–2.01 (m, 2H), 1.90 (ddd, *J* = 13.5, 7.0, 3.8 Hz, 2H), 1.82 (q, *J* = 7.3 Hz, 3H), 1.57 (qt, *J* = 12.6, 3.3 Hz, 3H), 1.43 (qd, *J* = 12.1, 3.3 Hz, 2H), 1.33 (t, *J* = 7.5 Hz, 3H), 1.12 (s, 6H), 1.03 (t, *J* = 7.3 Hz, 3H). ^{13}C NMR (101 MHz, $CDCl_3$) δ 193.19, 164.88, 155.84, 153.89, 149.33, 142.70, 141.79, 141.17, 120.41, 118.80, 117.70, 116.74, 104.66, 52.53, 40.00, 37.62, 35.89, 34.77 (2), 34.66, 28.45 (2), 27.28 (2), 26.44, 21.39, 21.35, 13.50, 12.94. HRMS (ESI/Q-TOF) m/z: [M + H]+ Calcd for $C_{29}H_{38}N_3O_2$ 460.2959; Found 460.2943. R_f = 0.61 (50% ethyl acetate in hexanes)

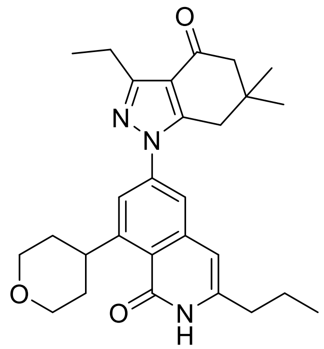

*6-(3-ethyl-6,6-dimethyl-4-oxo-4,5,6,7-tetrahydro-1H-indazol-1-yl)-3-propyl-8-(tetrahydro-2H-pyran-4-yl)isoquinolin-1(2H)-one (**13j**).* Yield 9%, 15 mg; ^{1}H NMR (400 MHz, Chloroform-*d*) δ 11.13–10.99 (m, 1H), 7.47 (q, *J* = 2.2 Hz, 2H), 6.36 (s, 1H), 4.88 (t, *J* = 11.7 Hz, 1H), 4.14 (dd, *J* = 11.4, 4.0 Hz, 2H), 3.69 (t, *J* = 11.3 Hz, 2H), 2.99 (q, *J* = 7.5 Hz, 2H), 2.88 (s, 2H), 2.64 (t, *J* = 7.4 Hz, 2H), 2.44 (s, 2H), 1.97 (d, *J* = 12.5 Hz, 2H), 1.92 – 1.75 (m, 4H), 1.33 (t, *J* = 7.5 Hz, 3H), 1.13 (s, 6H), 1.04 (t, *J* = 7.3 Hz, 3H). ^{13}C NMR (101 MHz, $CDCl_3$) δ 193.14, 165.03, 155.98, 151.41, 149.29, 142.91, 141.96, 141.37, 120.23, 118.83, 118.11, 116.89, 104.90, 68.83 (2), 52.48, 37.66, 37.47, 35.92, 34.61, 34.24 (2), 28.48 (2), 21.38, 21.36, 13.54, 12.88. HRMS (ESI/Q-TOF) m/z: [M + H]+ Calcd for $C_{28}H_{35}N_3O_3$ 462.2751; Found 462.2749. R_f = 0.17 (50% ethyl acetate in hexanes)

1.1. ^{1}H and ^{13}C NMR Spectra for New Compounds

1.1.1. ^{1}H and ^{13}C NMR Spectra for **8b**

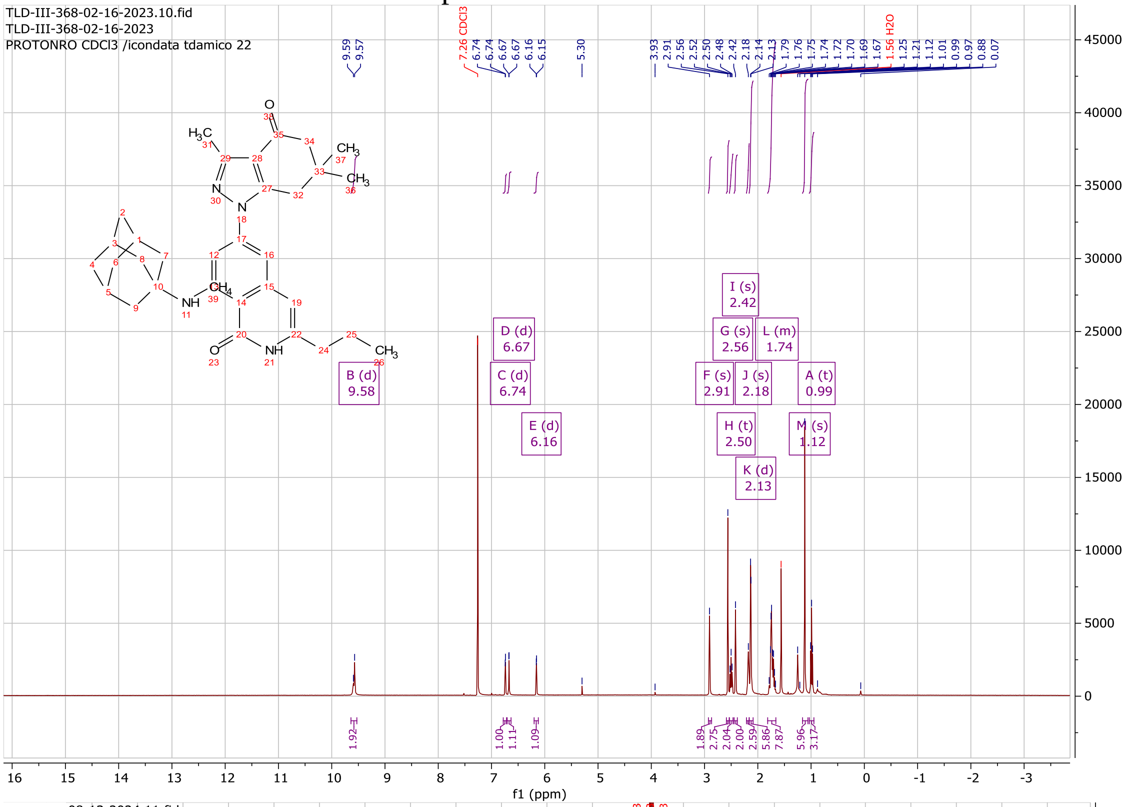

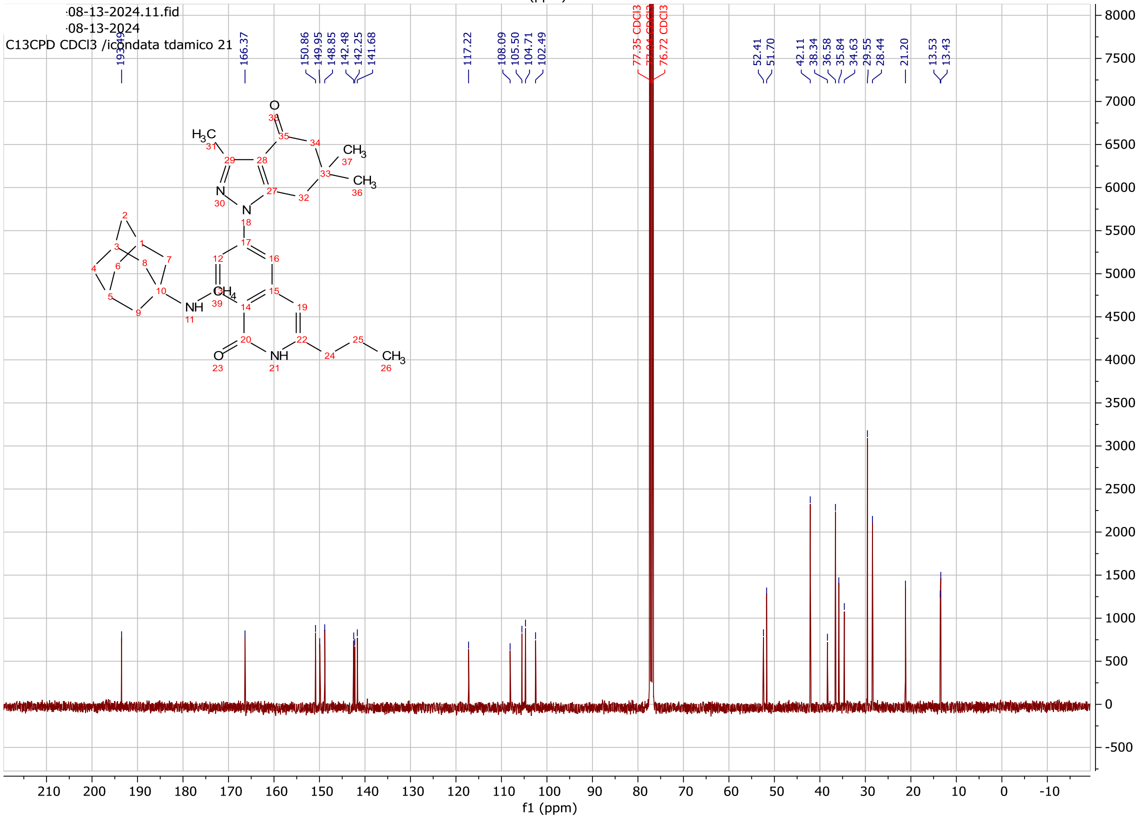

1.1.2. ^{1}H and ^{13}C NMR Spectra for **8c**

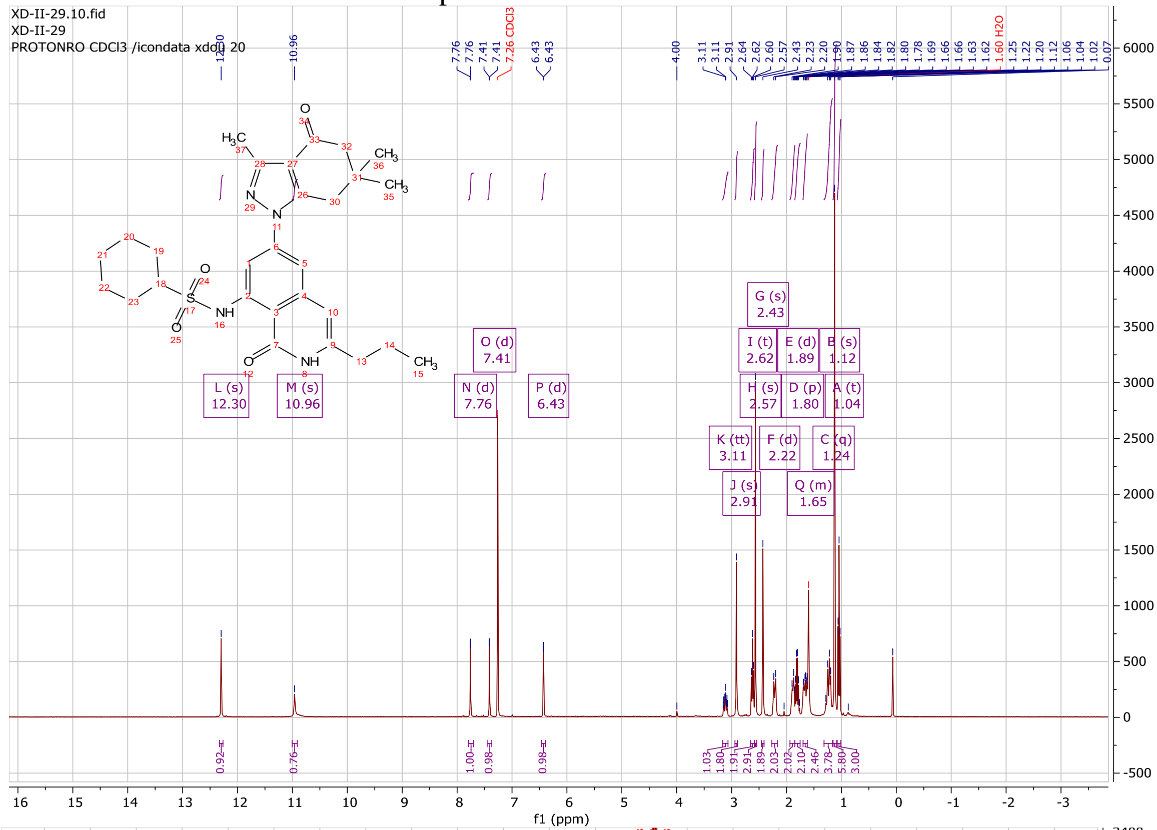

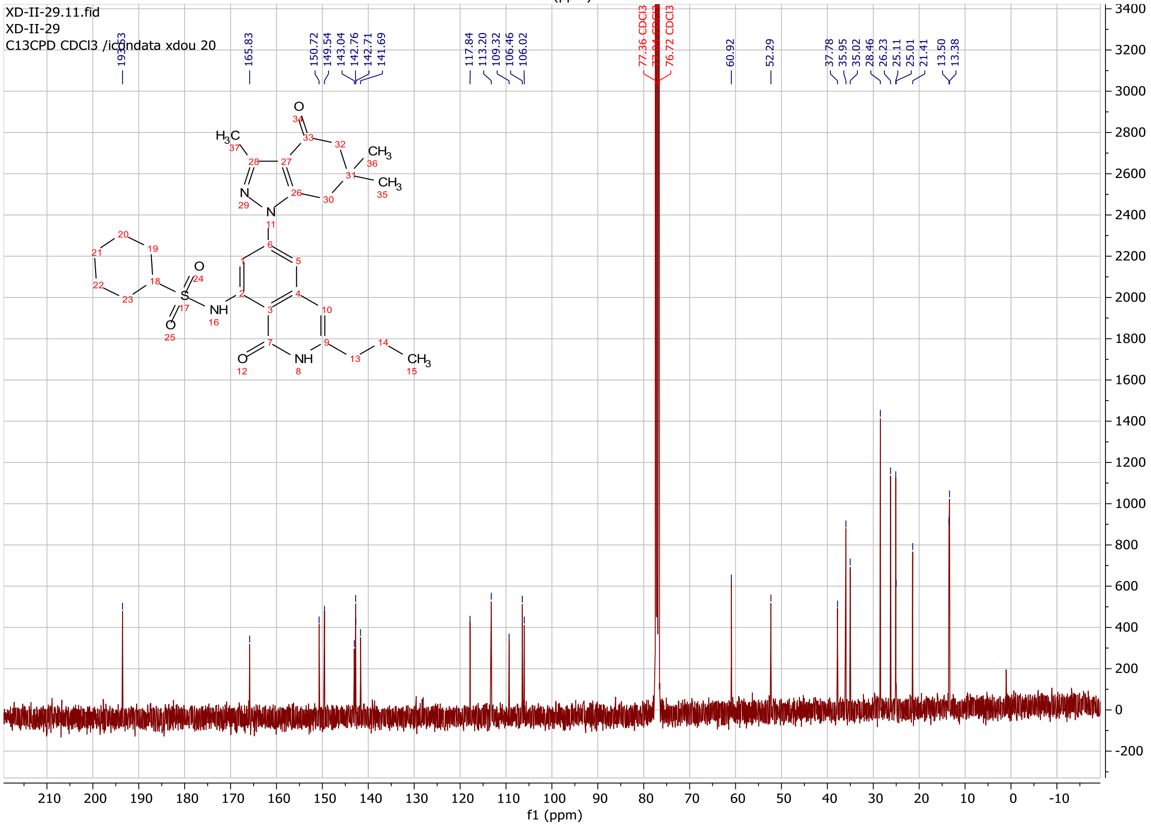

1.1.3. ^{1}H and ^{13}C NMR Spectra for **8d**

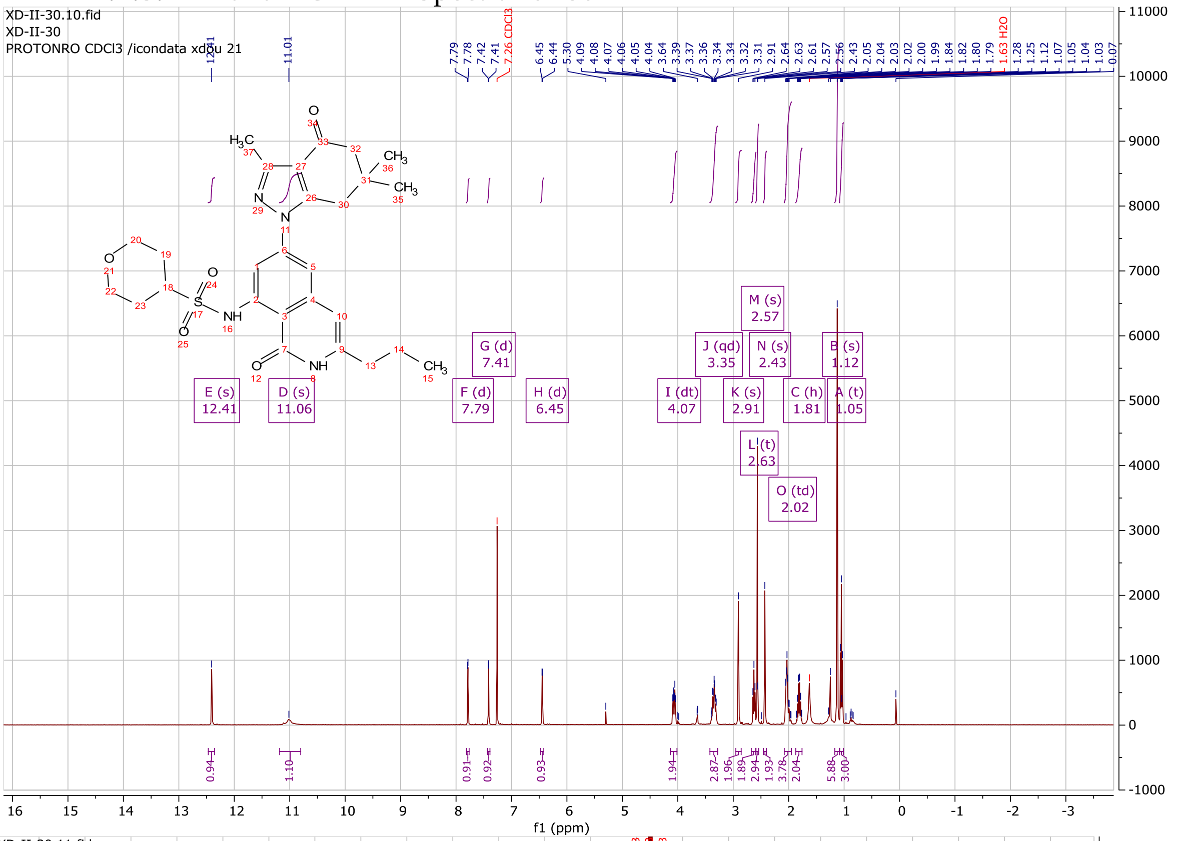

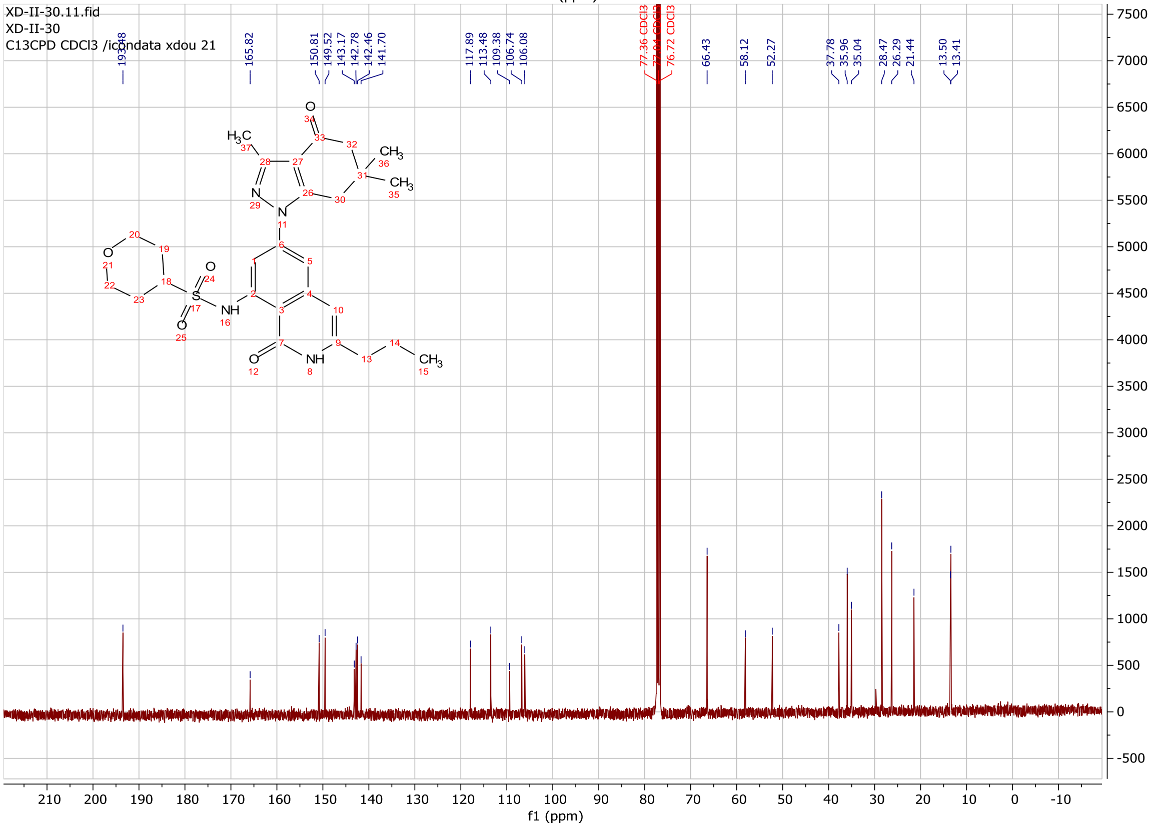

1.1.4. ^{1}H, ^{13}C, and ^{19}F NMR Spectra for **8e**

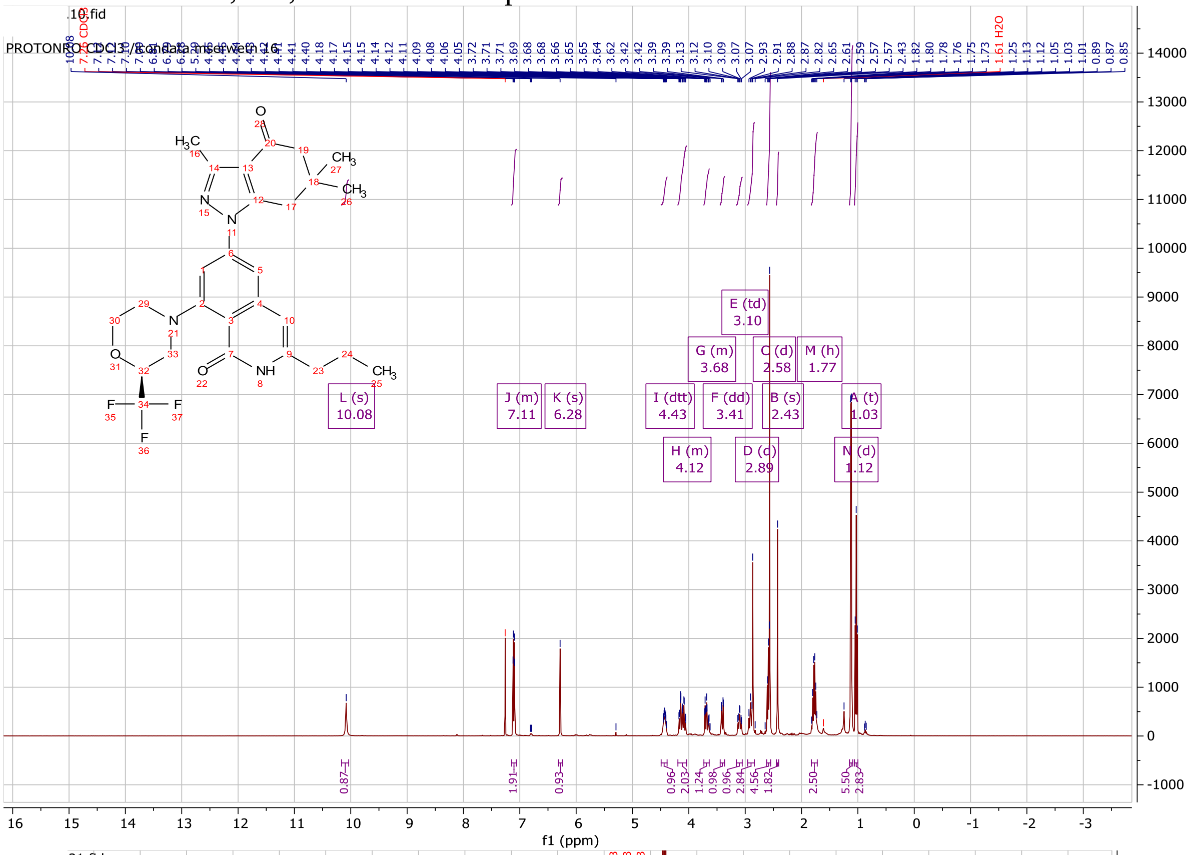

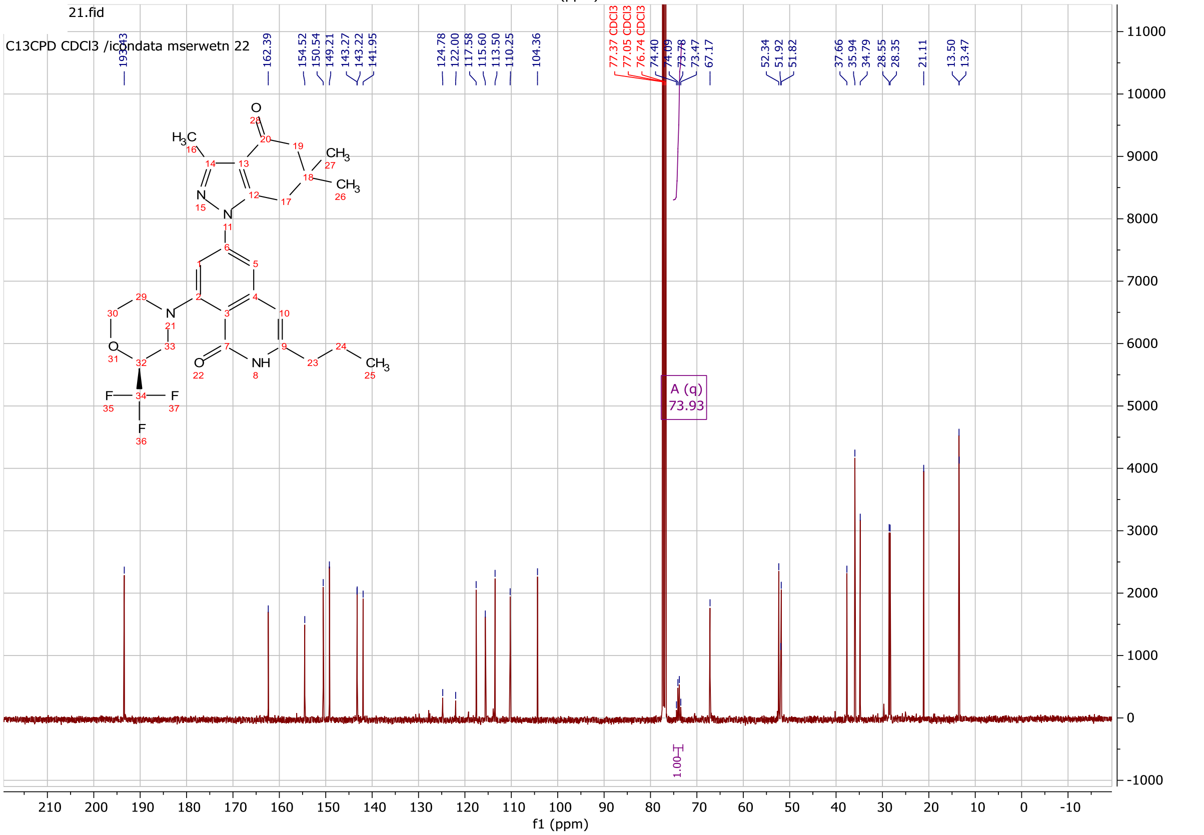

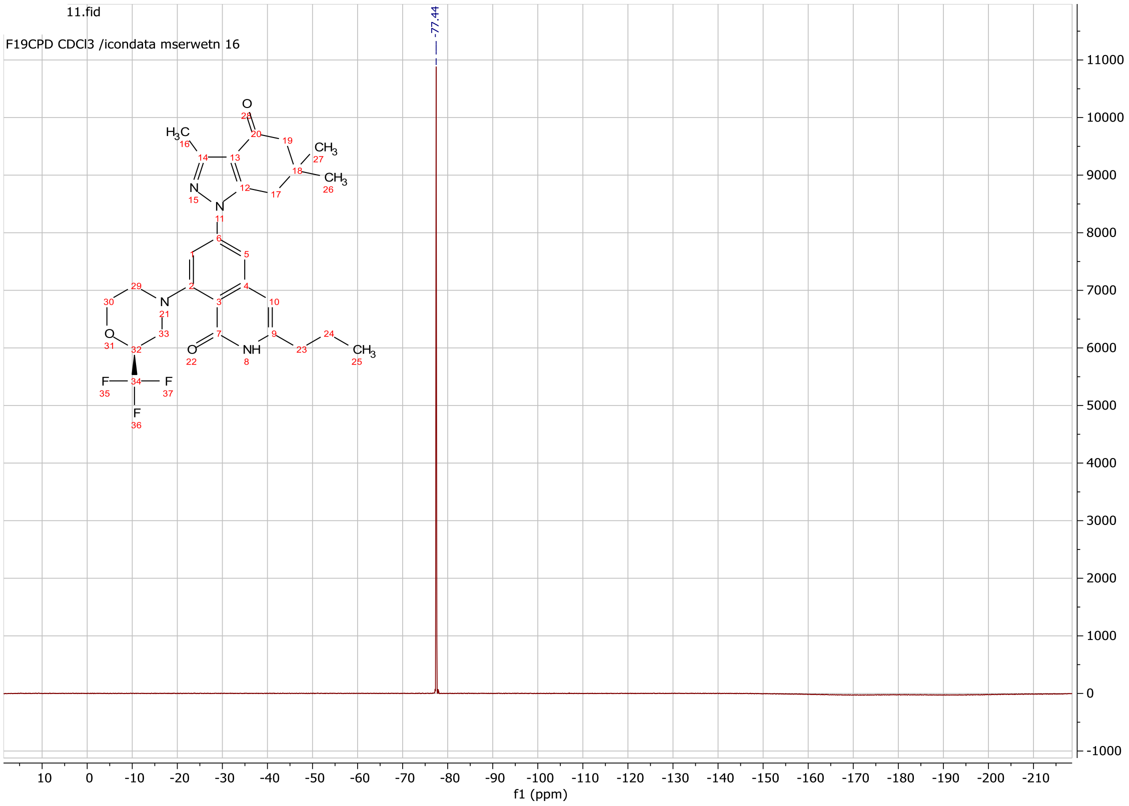

1.1.5. ^{1}H, ^{13}C, and ^{19}F NMR Spectra for **8f**

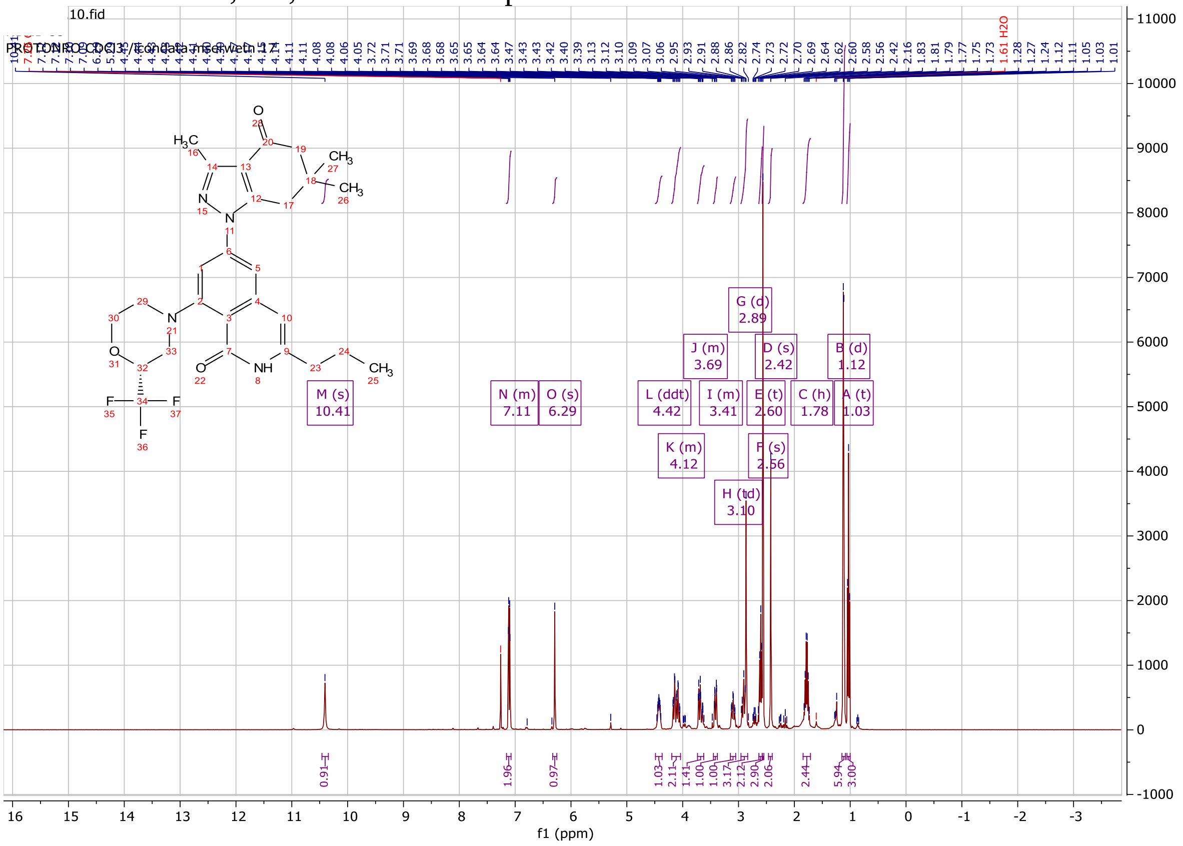

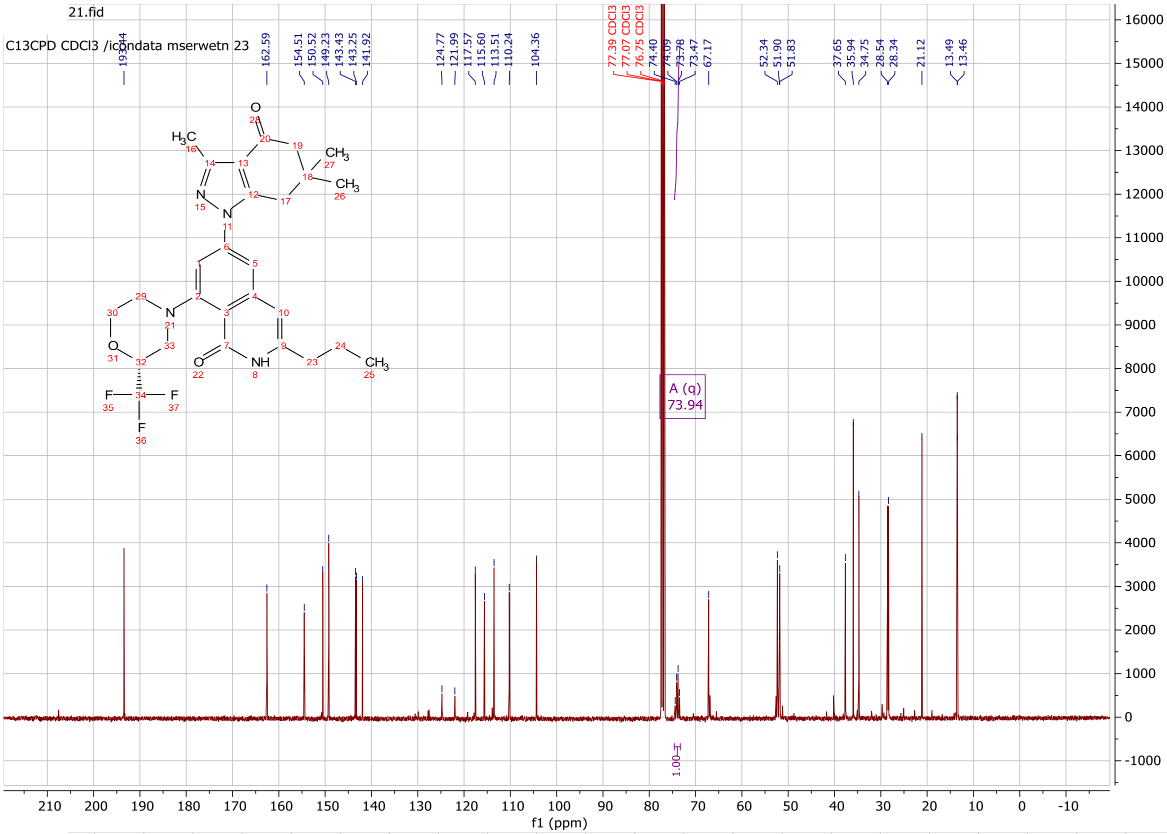
21.fid
C13CPD CDCl3 /icondata mserwetn 23
193.44
162.59
154.51
150.52
149.23
143.43
143.25
141.92
124.77
121.99
117.57
115.60
113.51
110.24
104.36
77.39 CDCl3
77.07 CDCl3
76.75 CDCl3
74.40
74.09
73.78
73.47
67.17
52.34
51.90
51.83
37.65
35.94
34.75
28.54
28.34
21.12
13.49
13.46
A (q)
73.94
1.00
f1 (ppm)

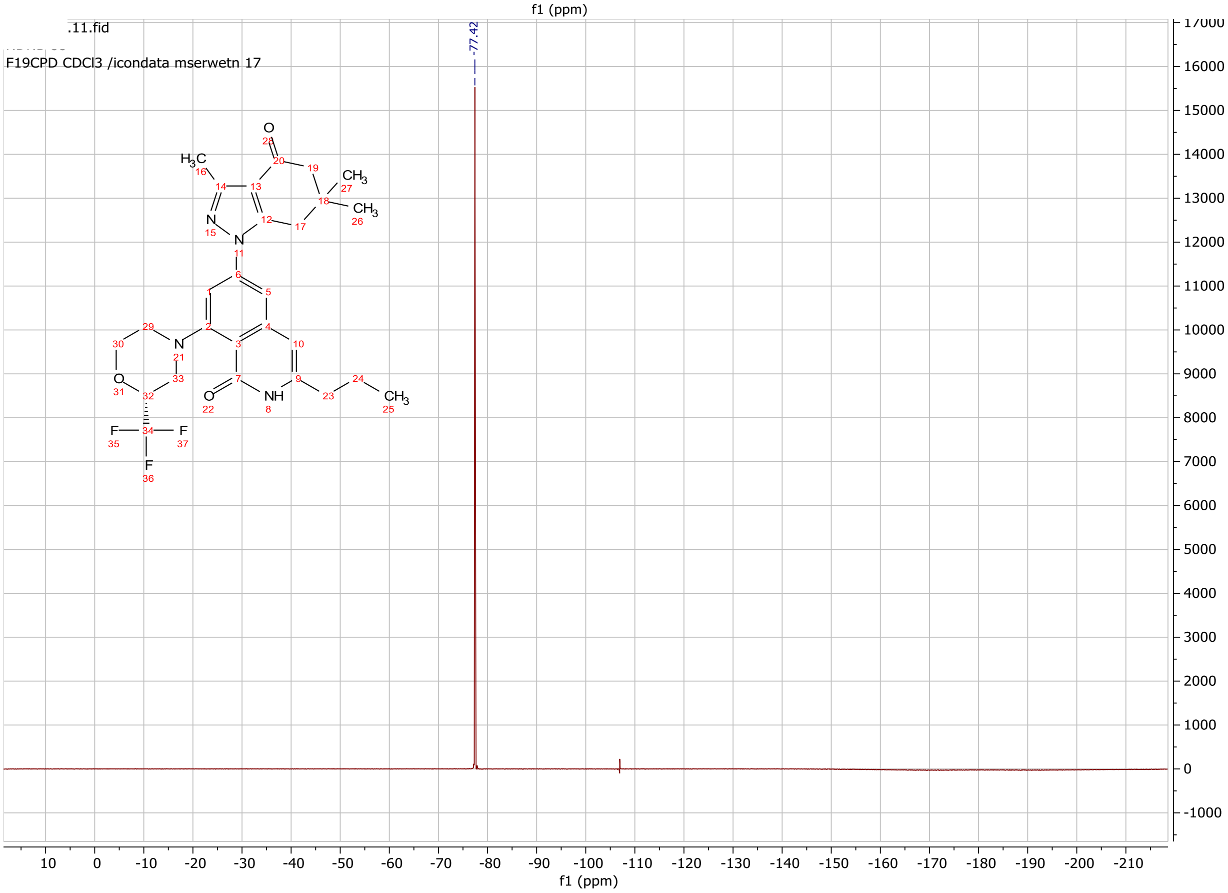
.11.fid
F19CPD CDCl3 /icondata mserwetn 17
-77.42
f1 (ppm)

1.1.6. ^{1}H and ^{13}C NMR Spectra for **8g**

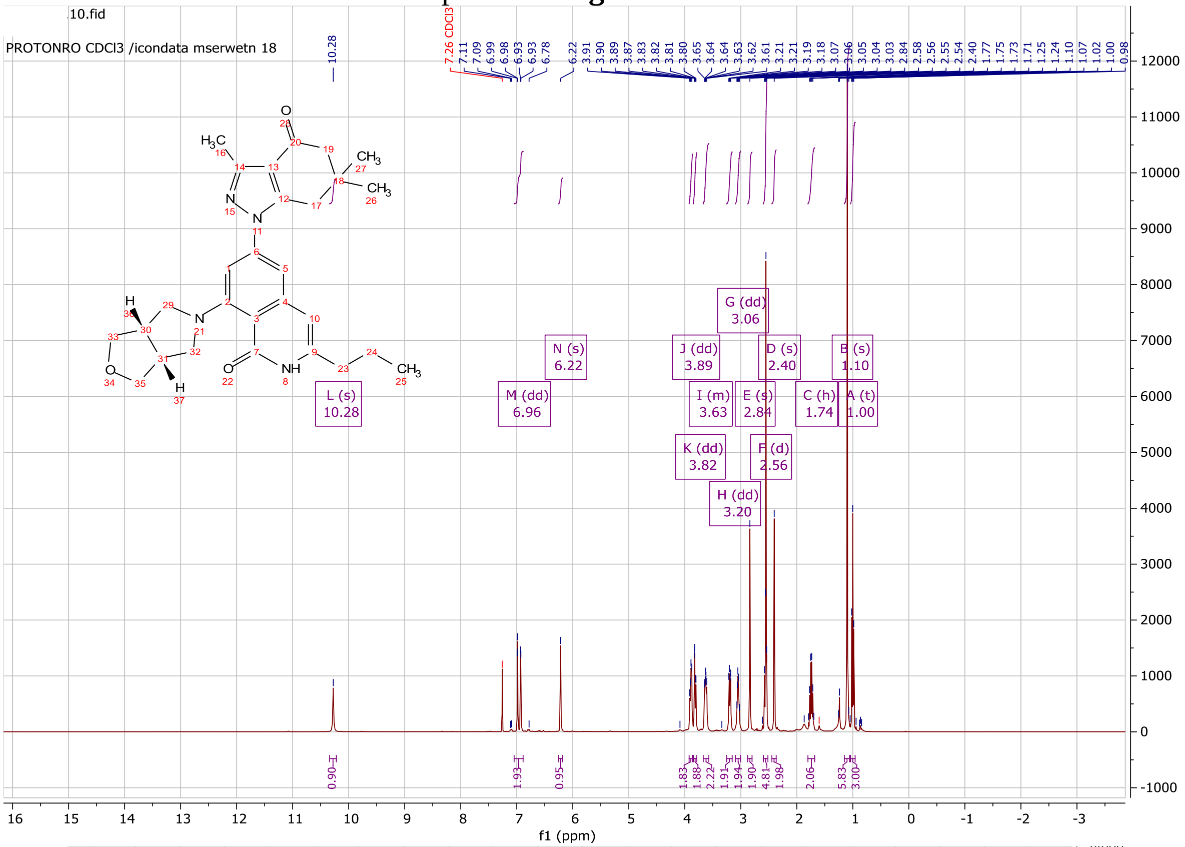

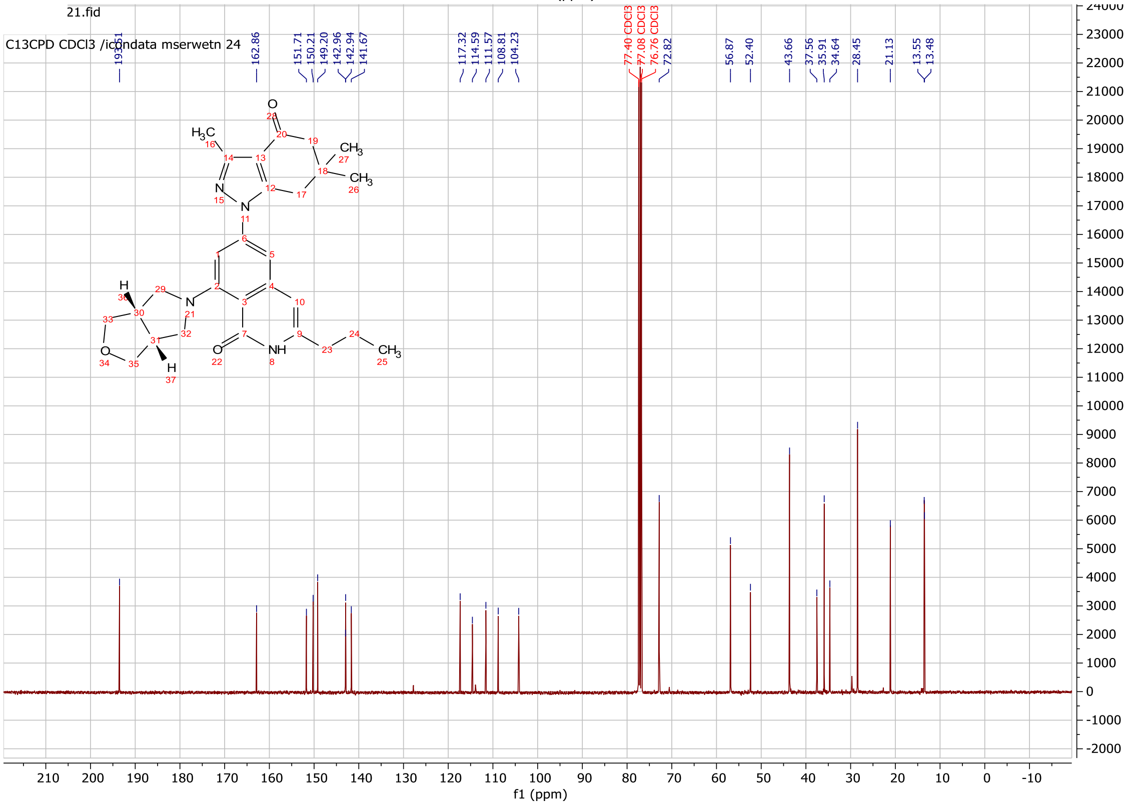

1.1.7. ^{1}H and ^{13}C NMR Spectra for **8h**

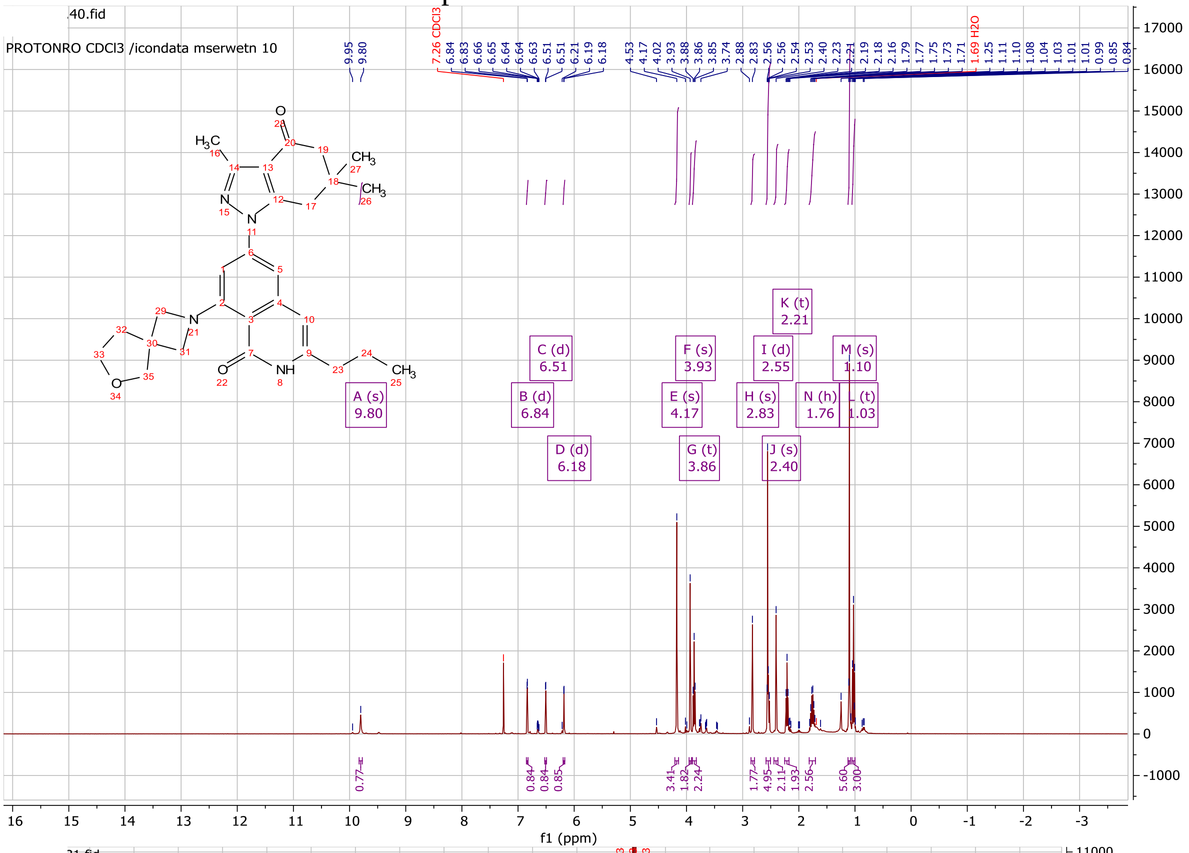

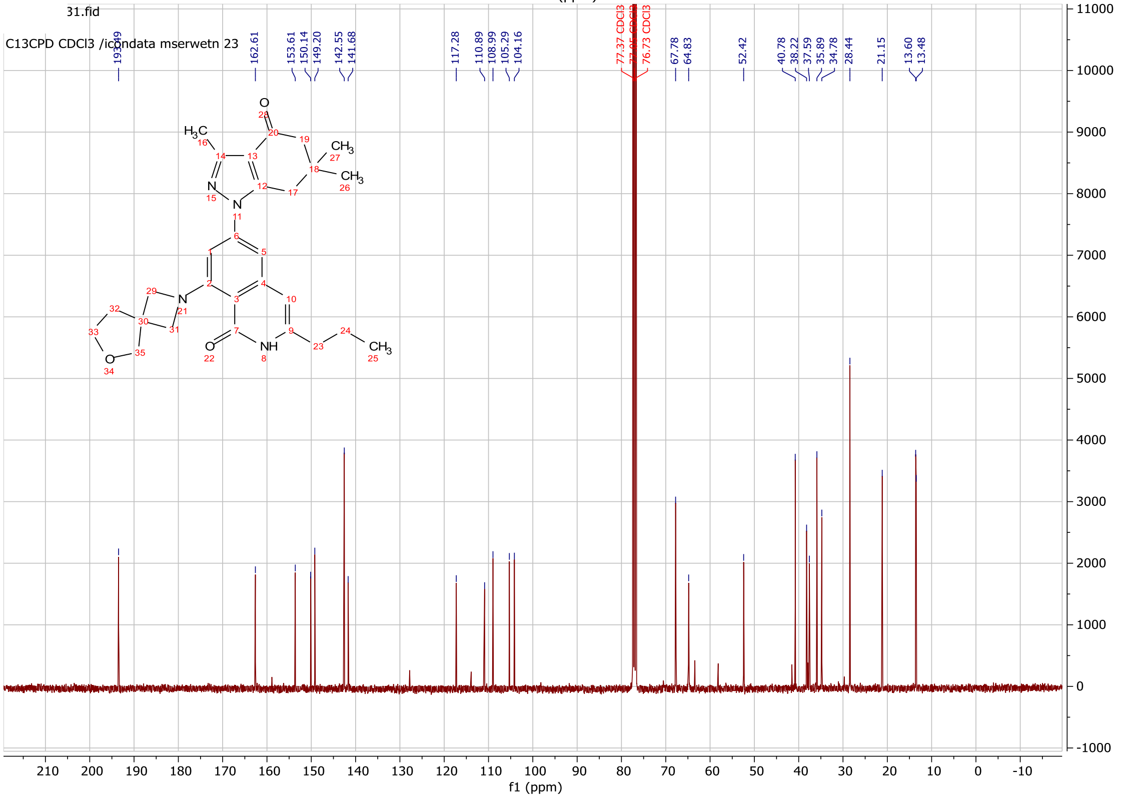

1.1.8. ^{1}H and ^{13}C NMR Spectra for **8i**

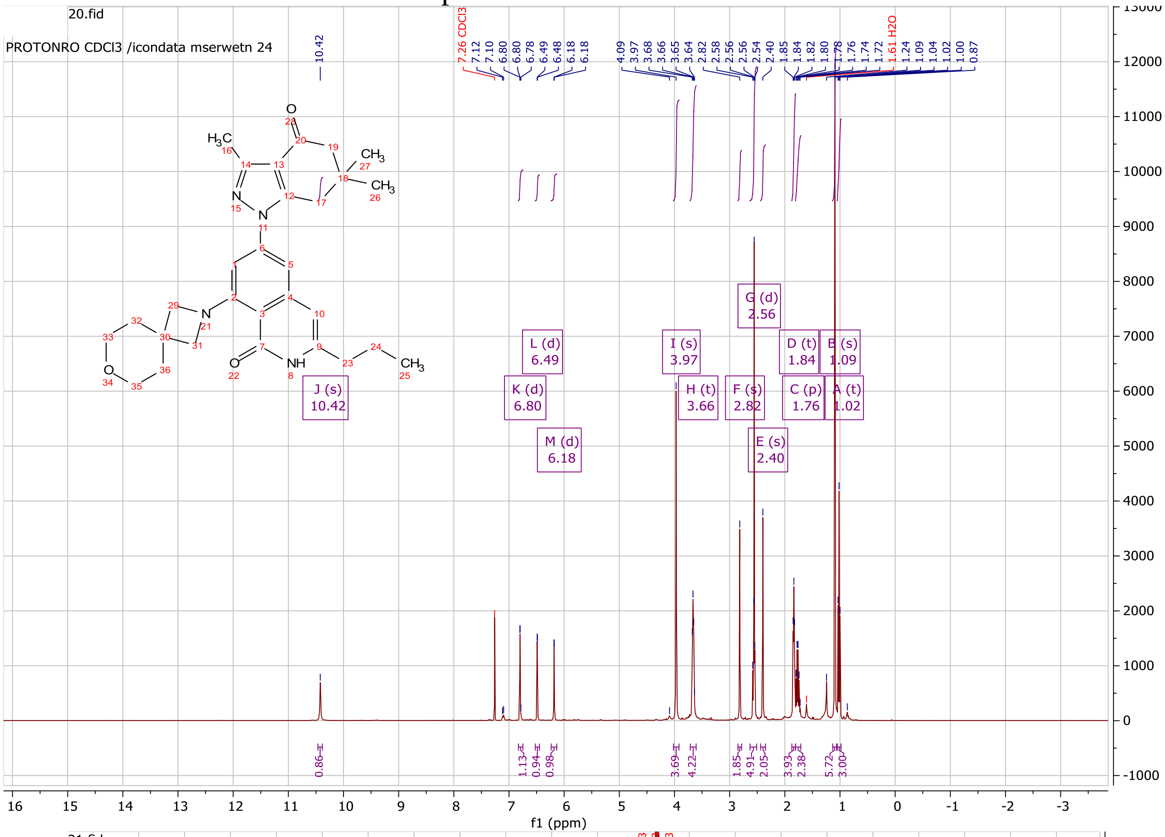

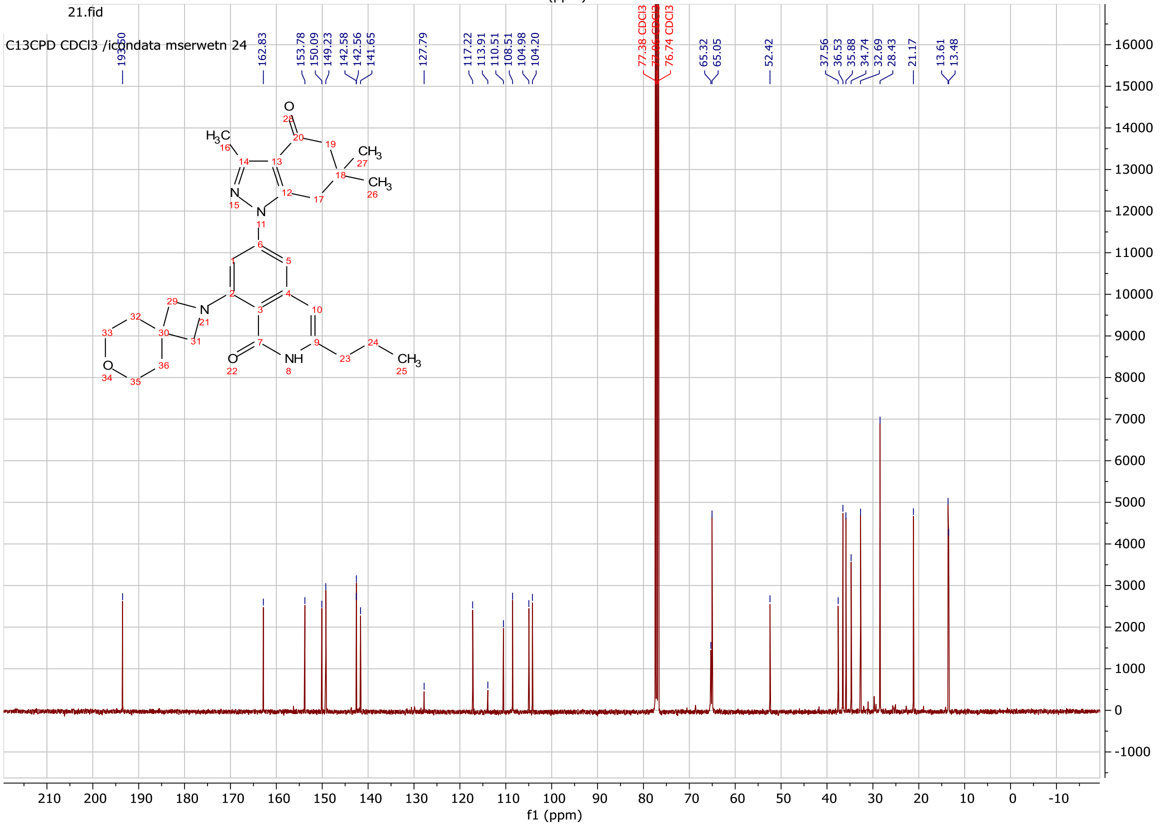

1.1.9. ^{1}H and ^{13}C NMR Spectra for **8j**

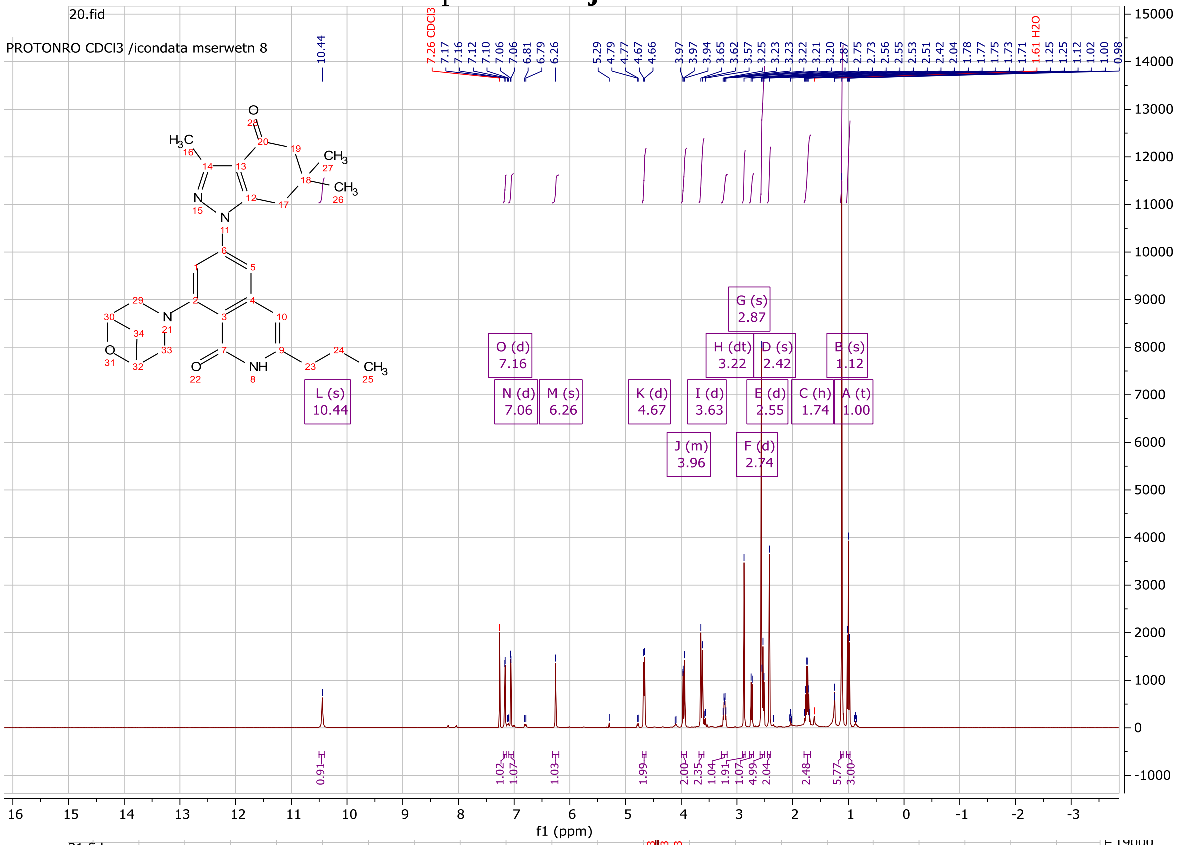

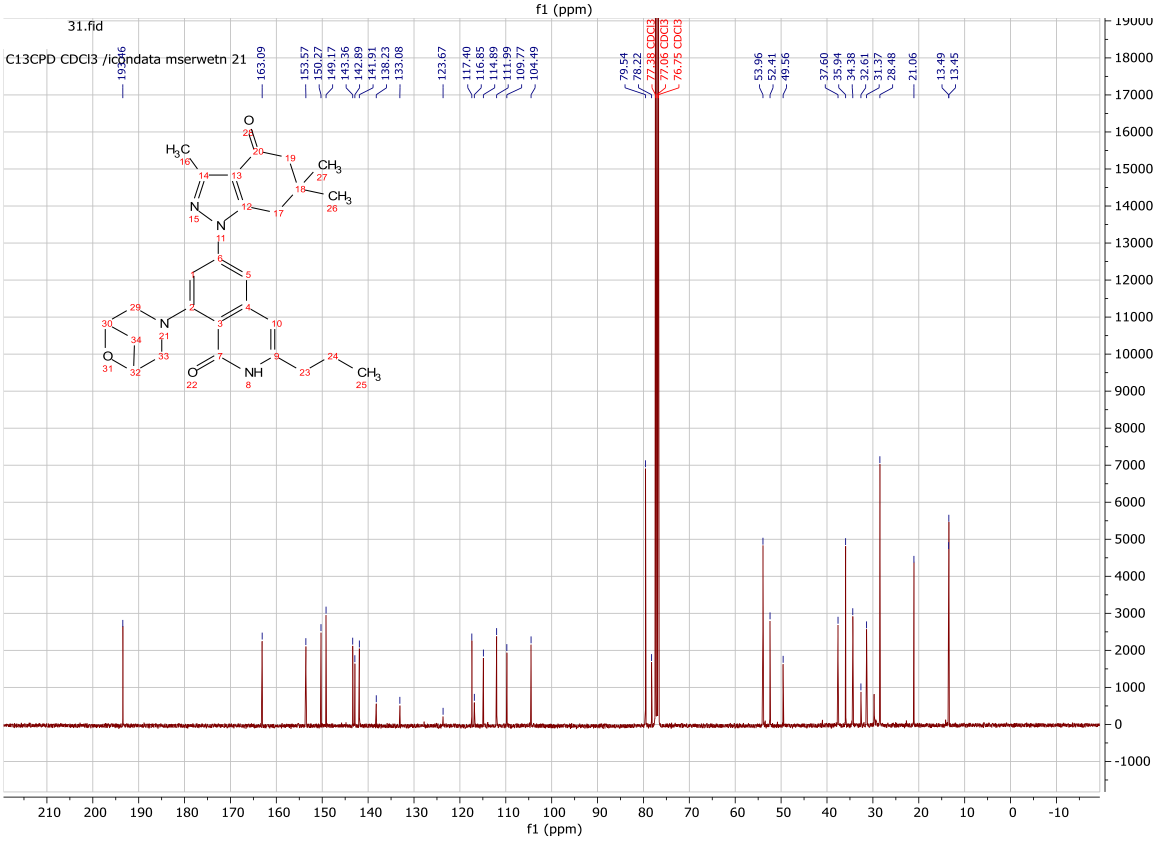

1.1.10. ^{1}H and ^{13}C NMR Spectra for **9a**

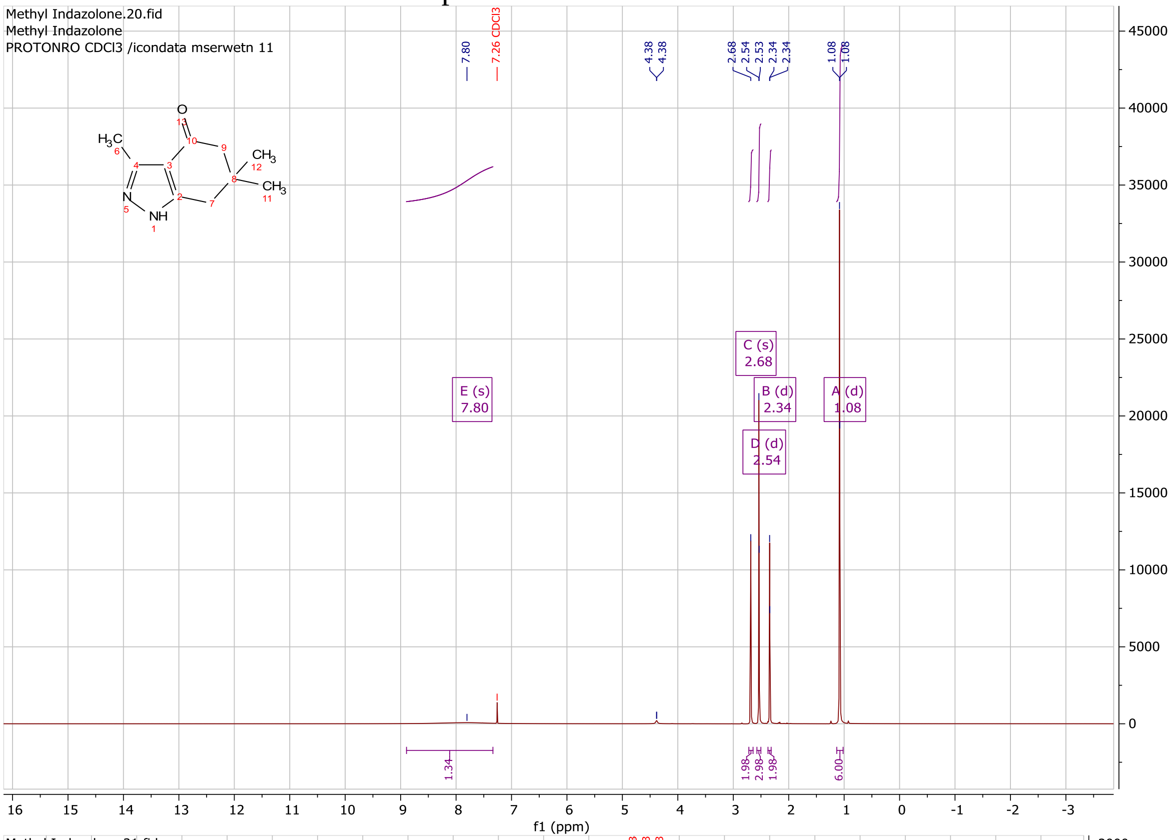

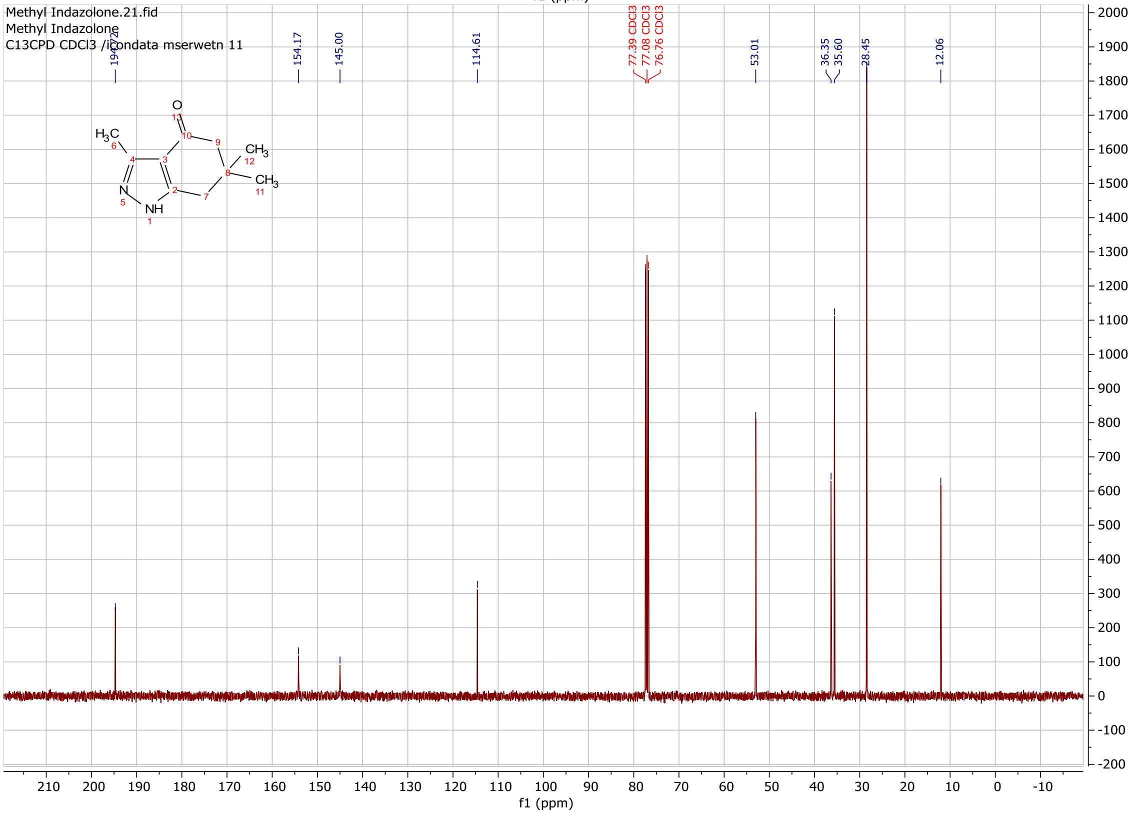

1.1.11. ^{1}H and ^{13}C NMR Spectra for **9b**

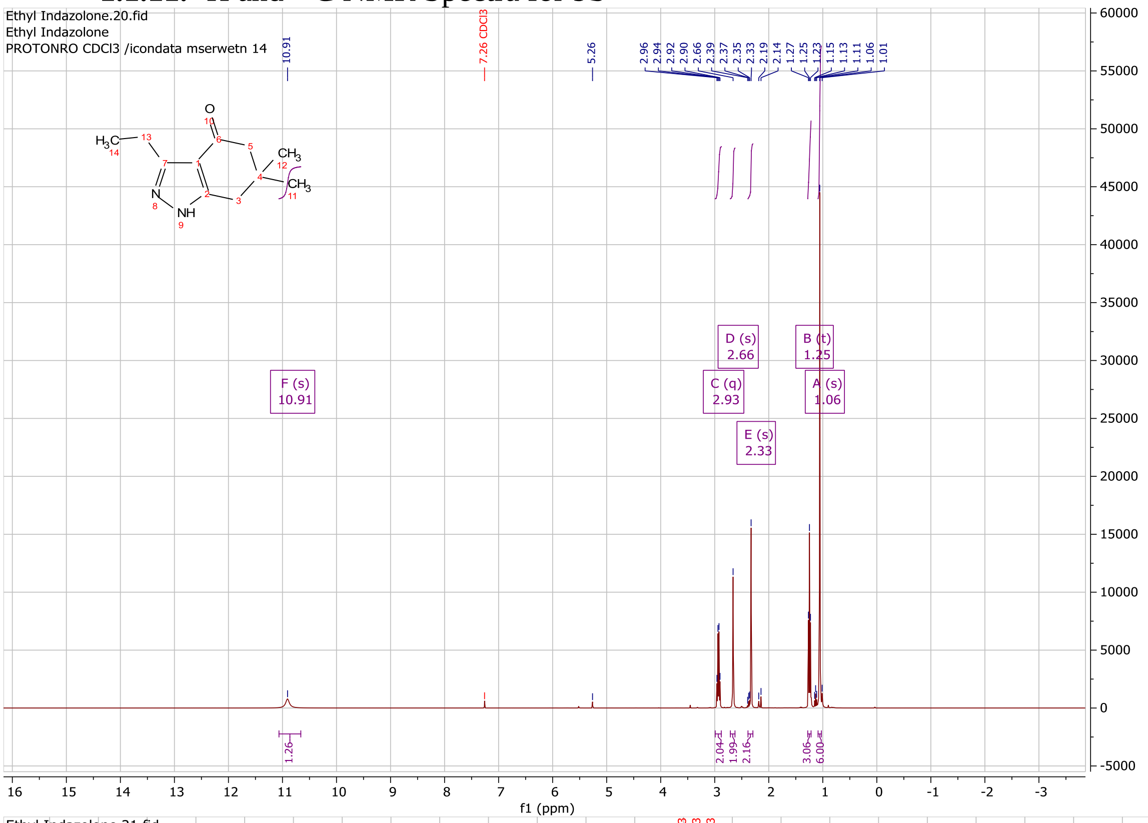

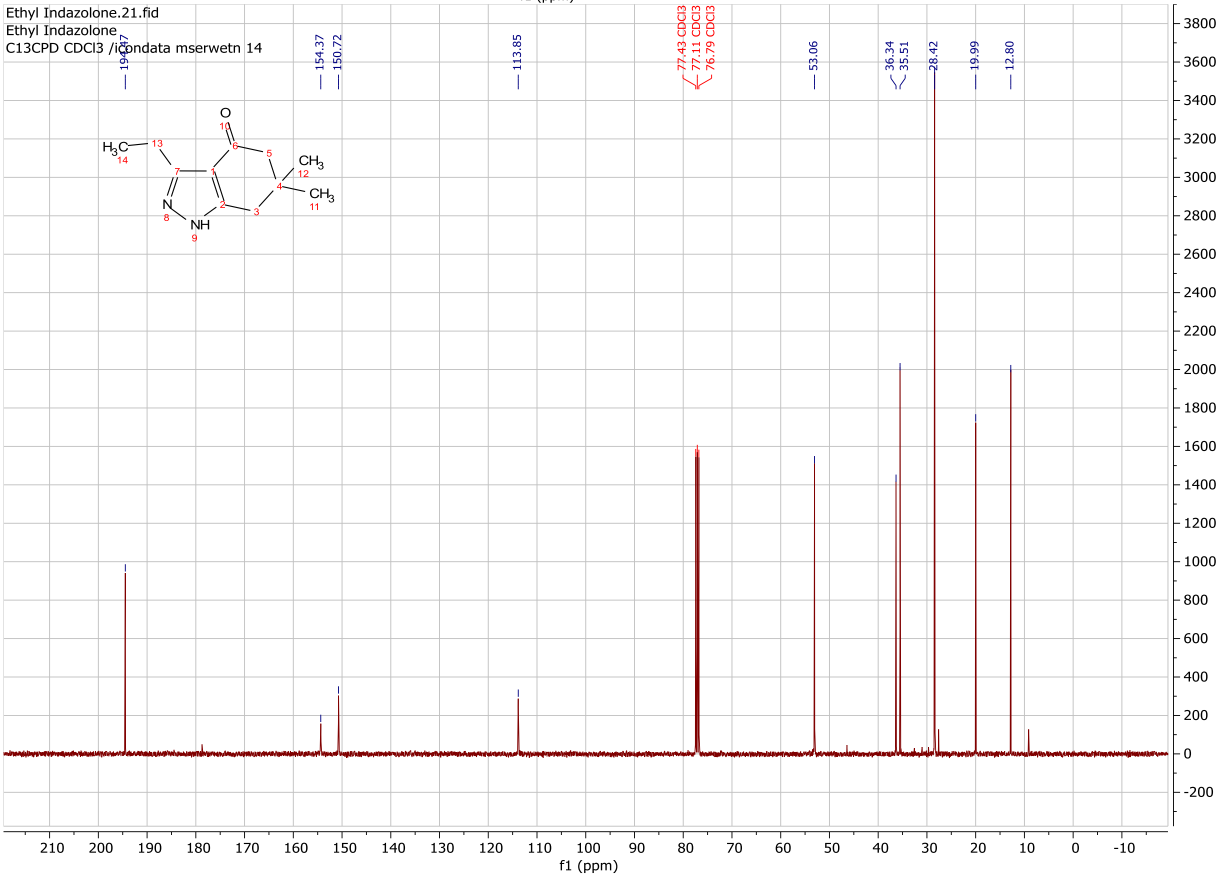

1.1.12. ^{1}H and ^{13}C NMR Spectra for **13a**

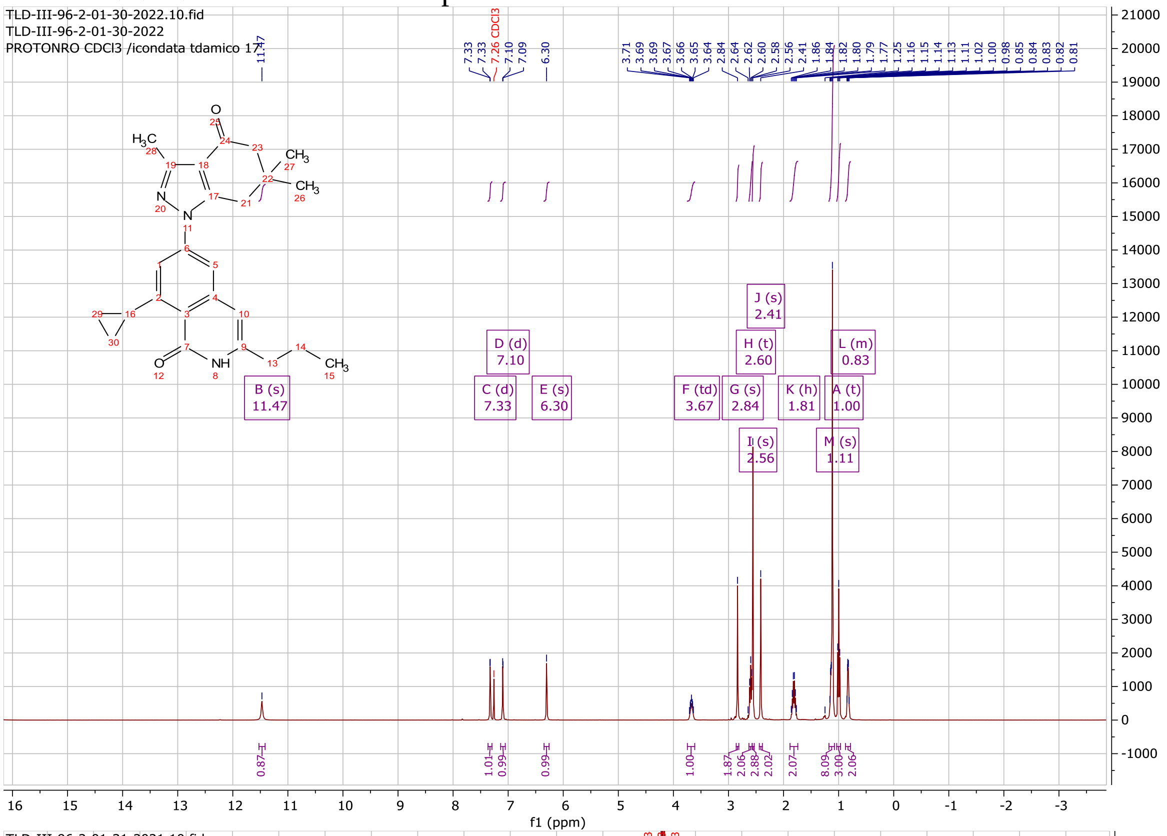

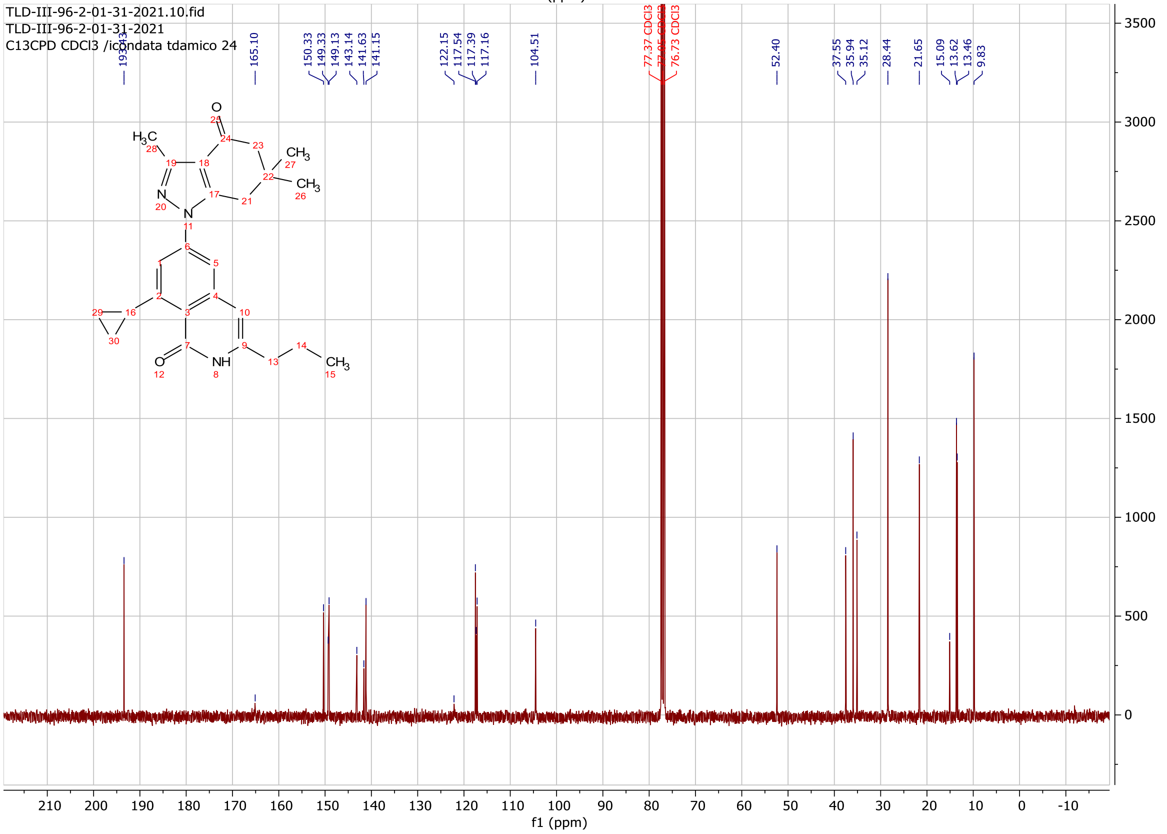

1.1.13. ^{1}H and ^{13}C NMR Spectra for **13b**

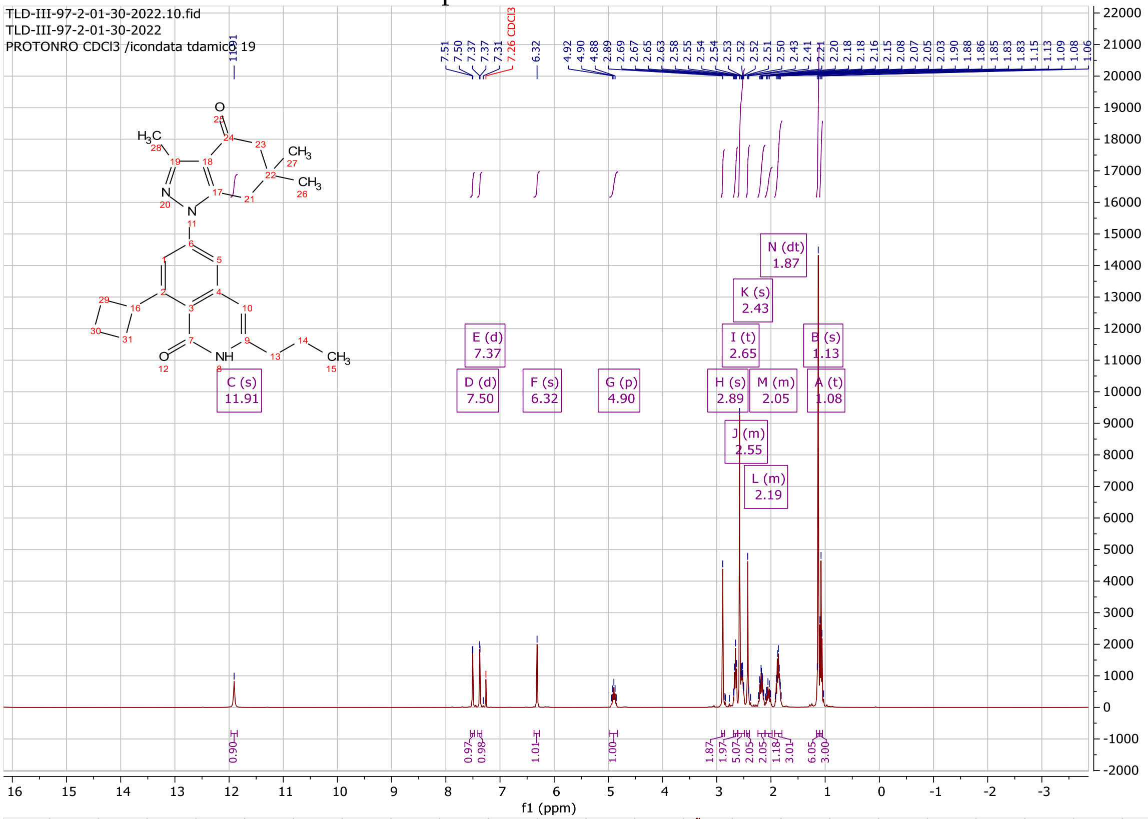

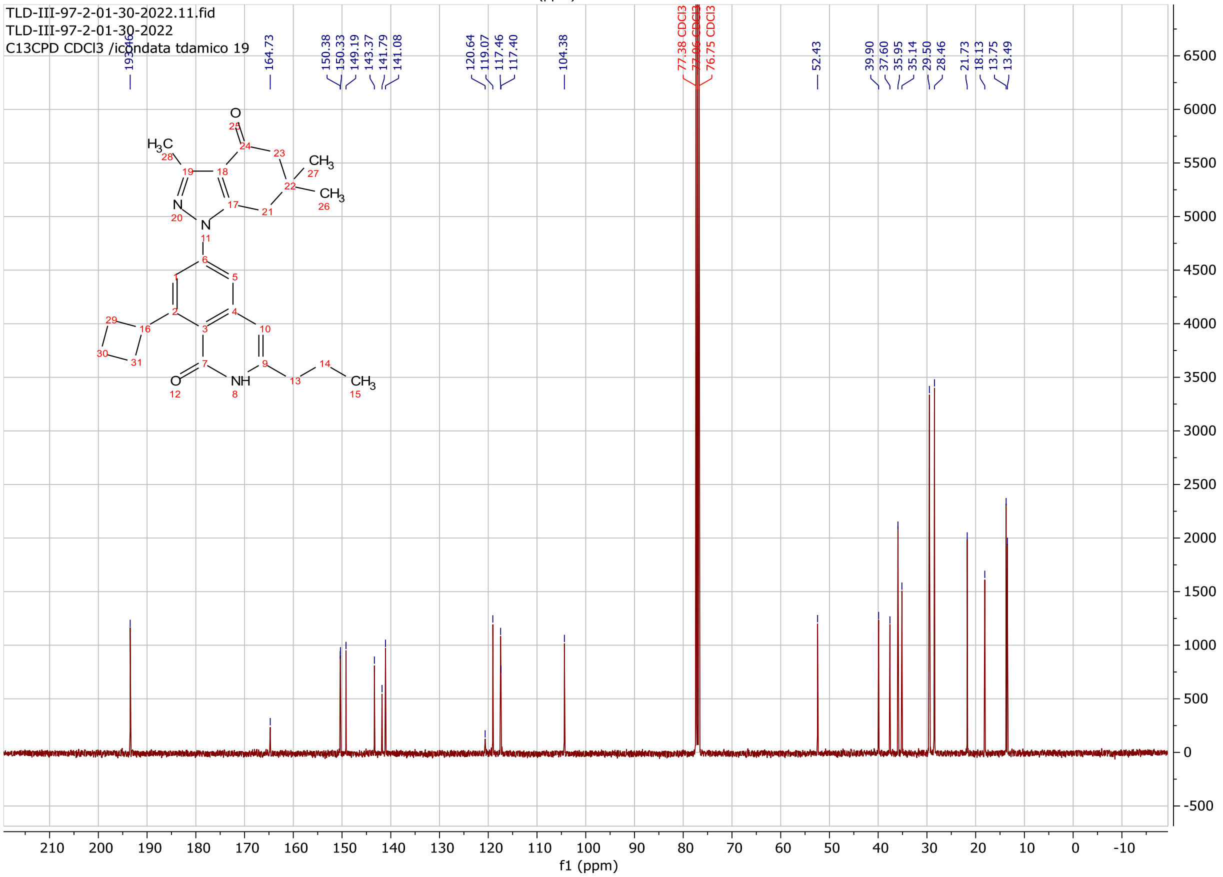

1.1.14. ^{1}H and ^{13}C NMR Spectra for **13c**

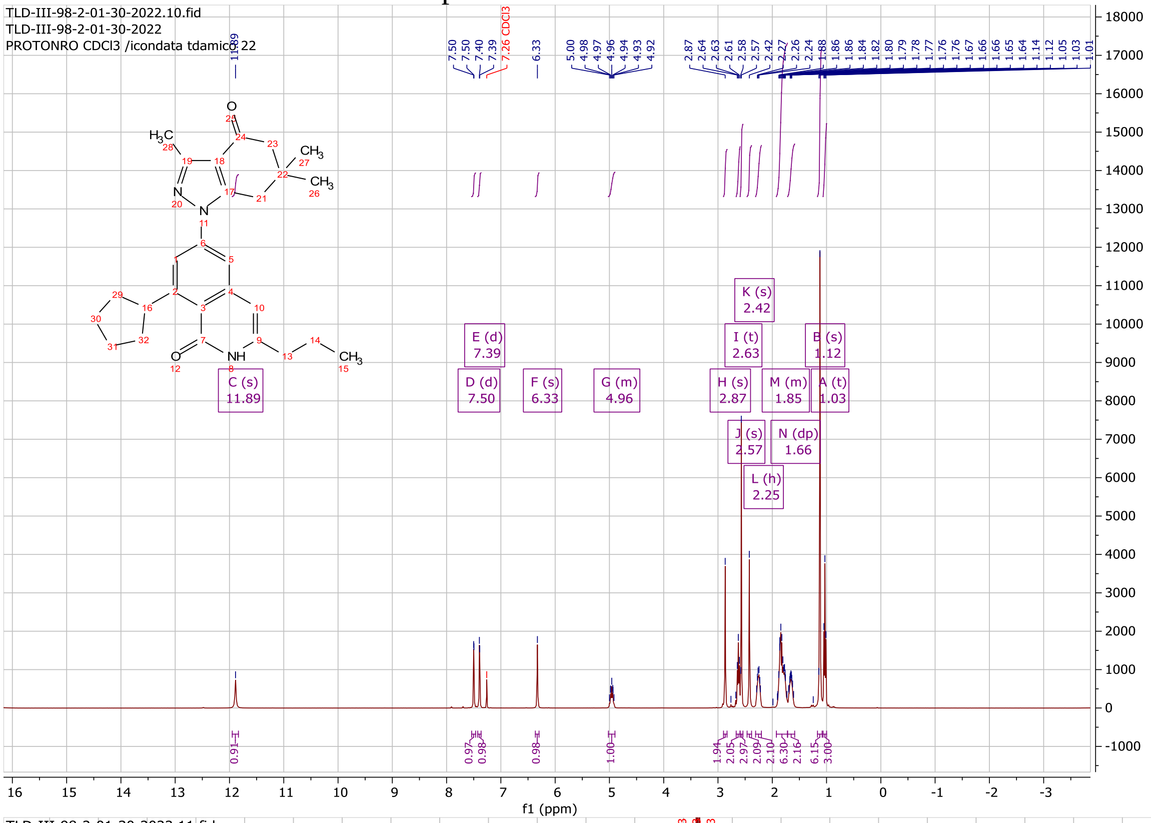

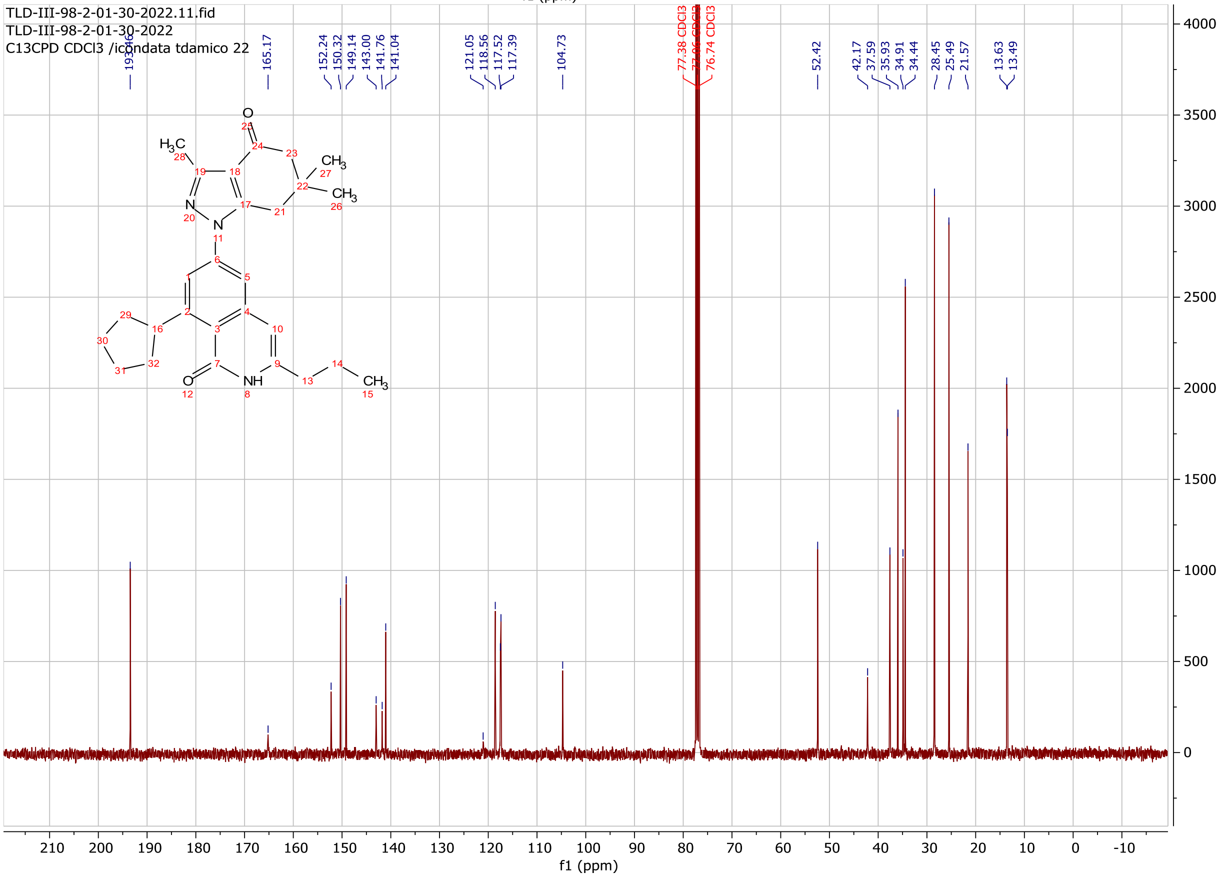

1.1.15. ^{1}H and ^{13}C NMR Spectra for **13d**

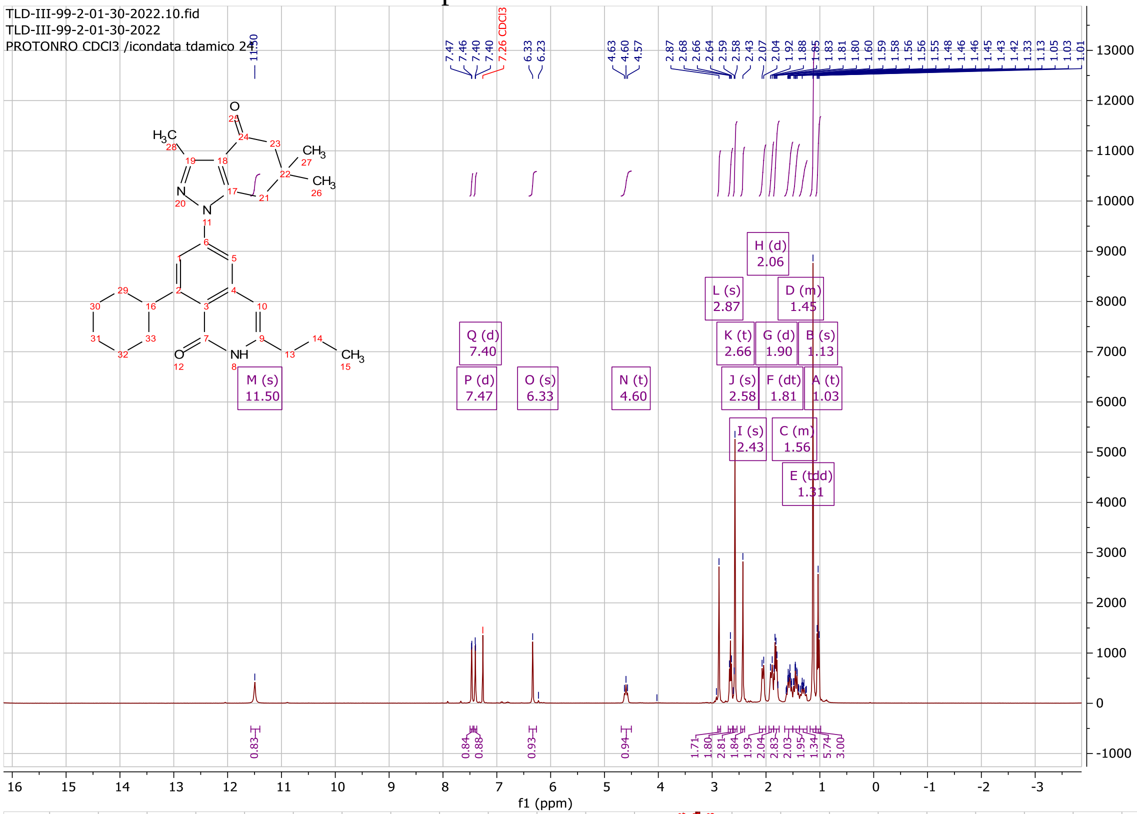

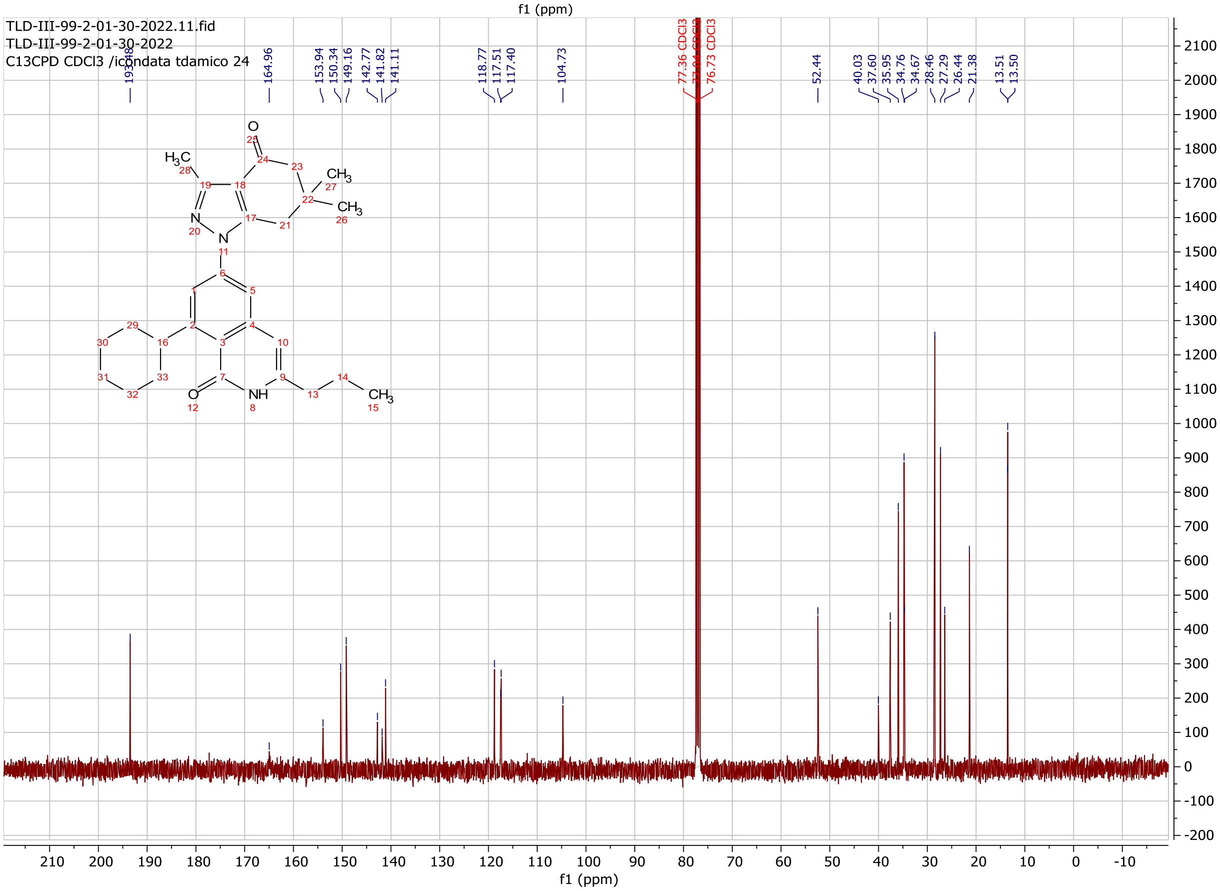

1.1.16. ^{1}H and ^{13}C NMR Spectra for **13e**

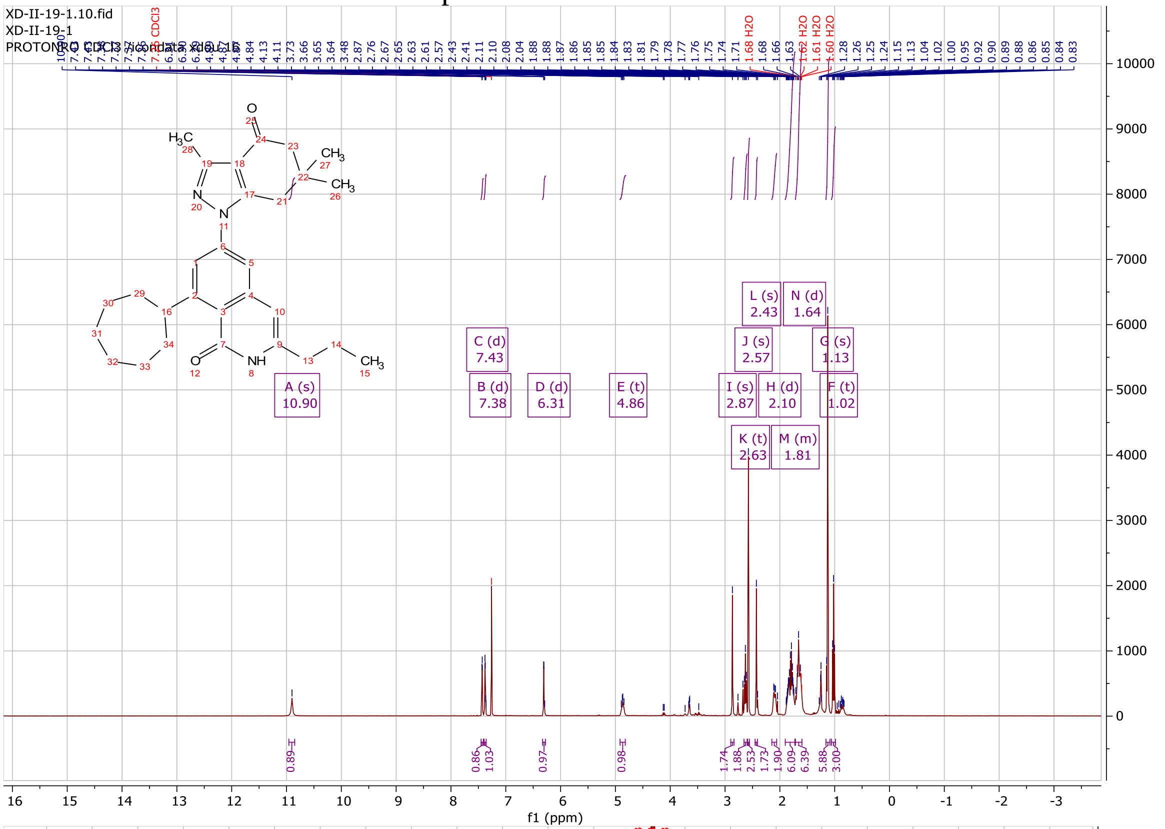

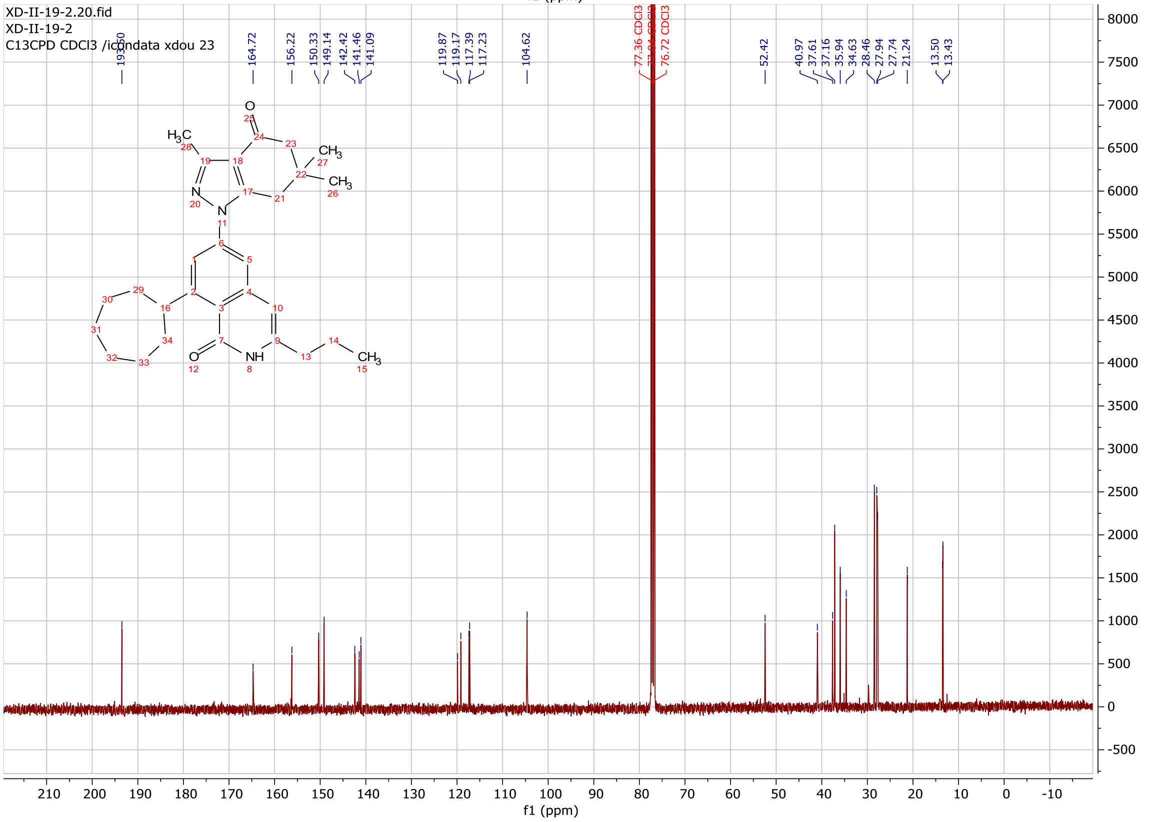

1.1.17. ^{1}H and ^{13}C NMR Spectra for **13f**

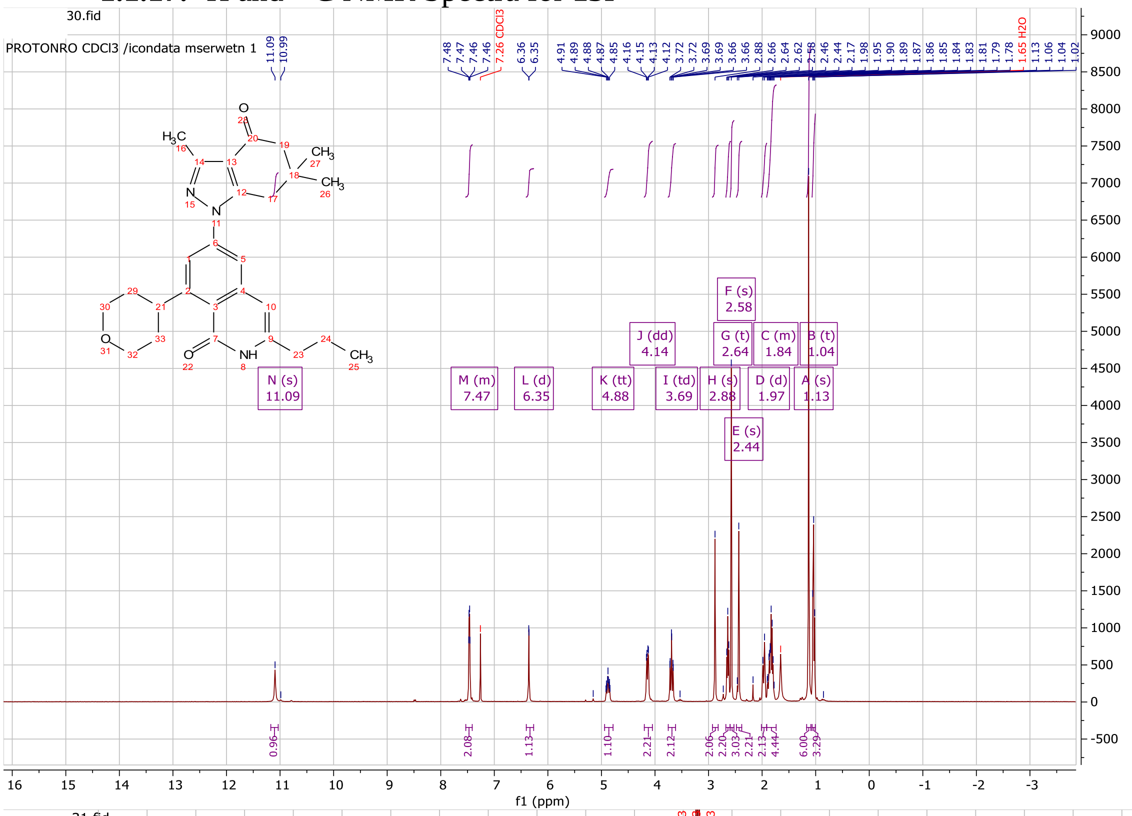

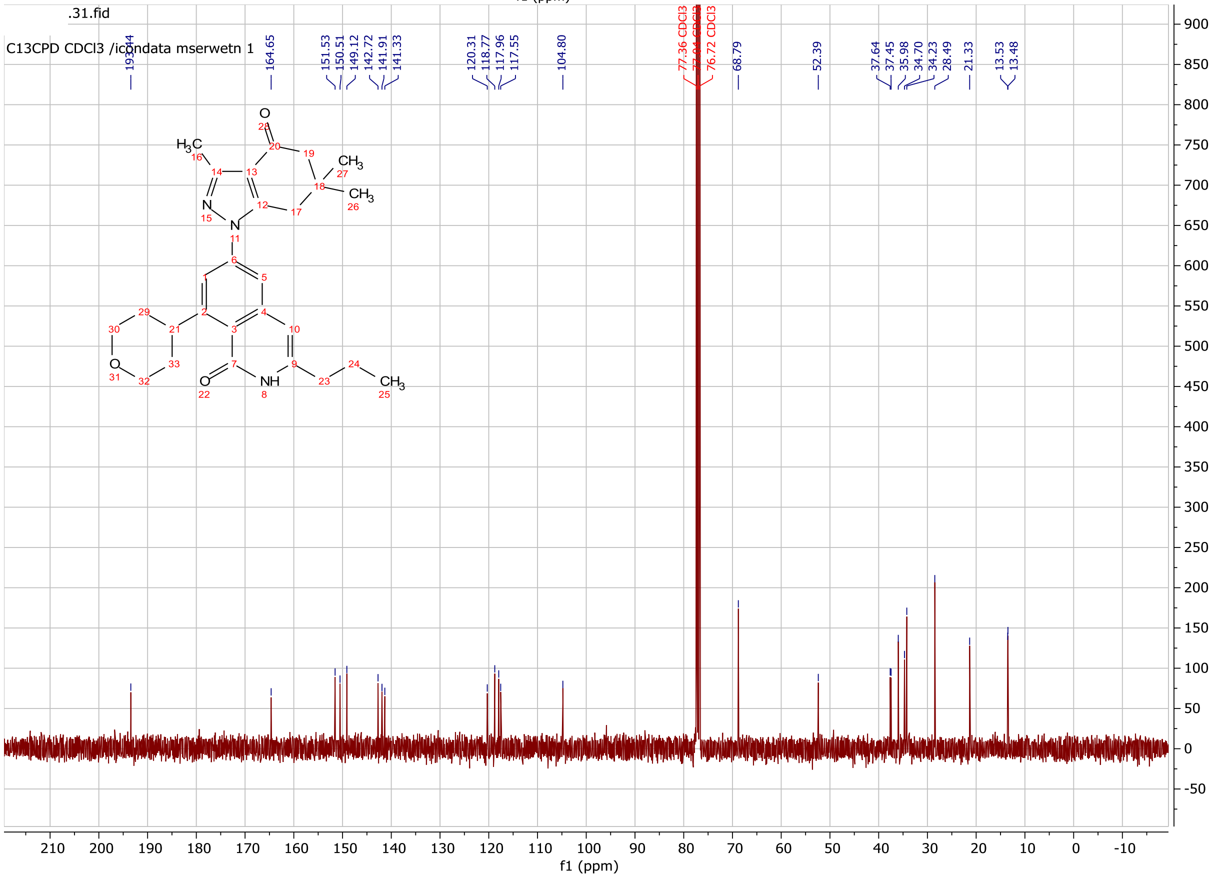

1.1.18. ^{1}H and ^{13}C NMR Spectra for **13g**

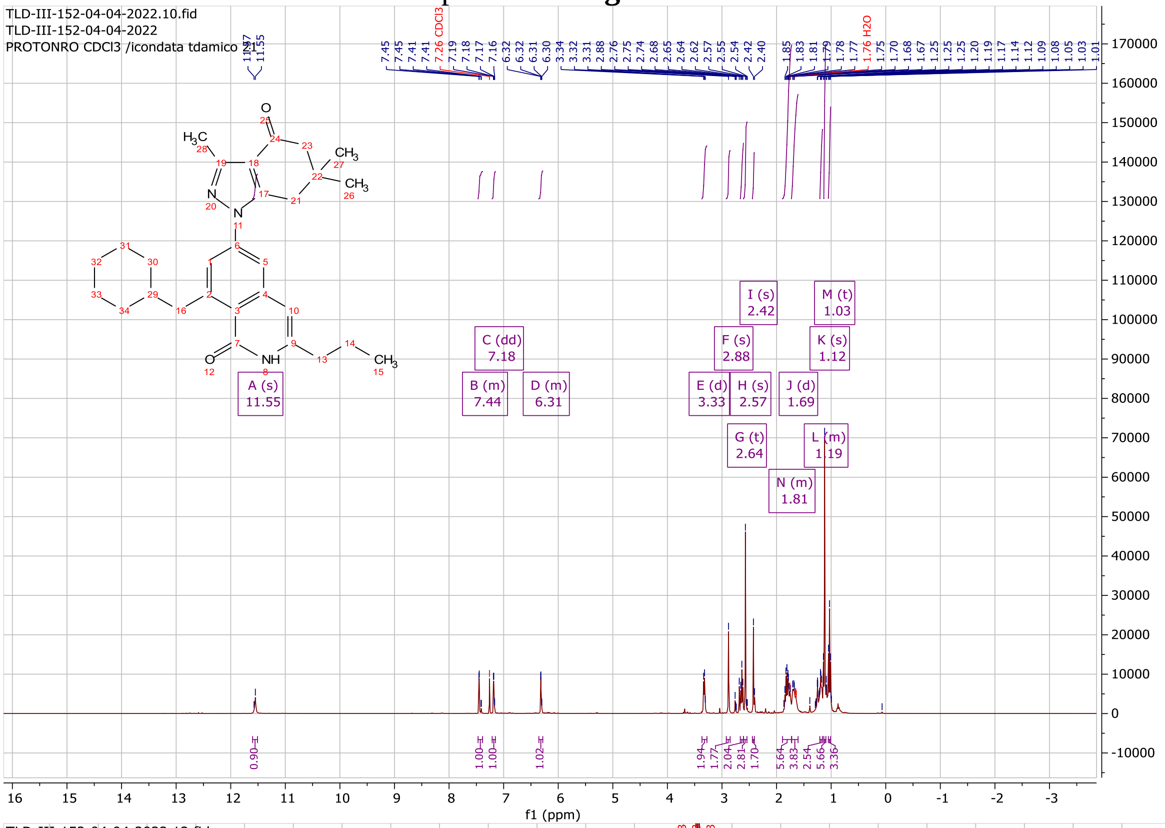

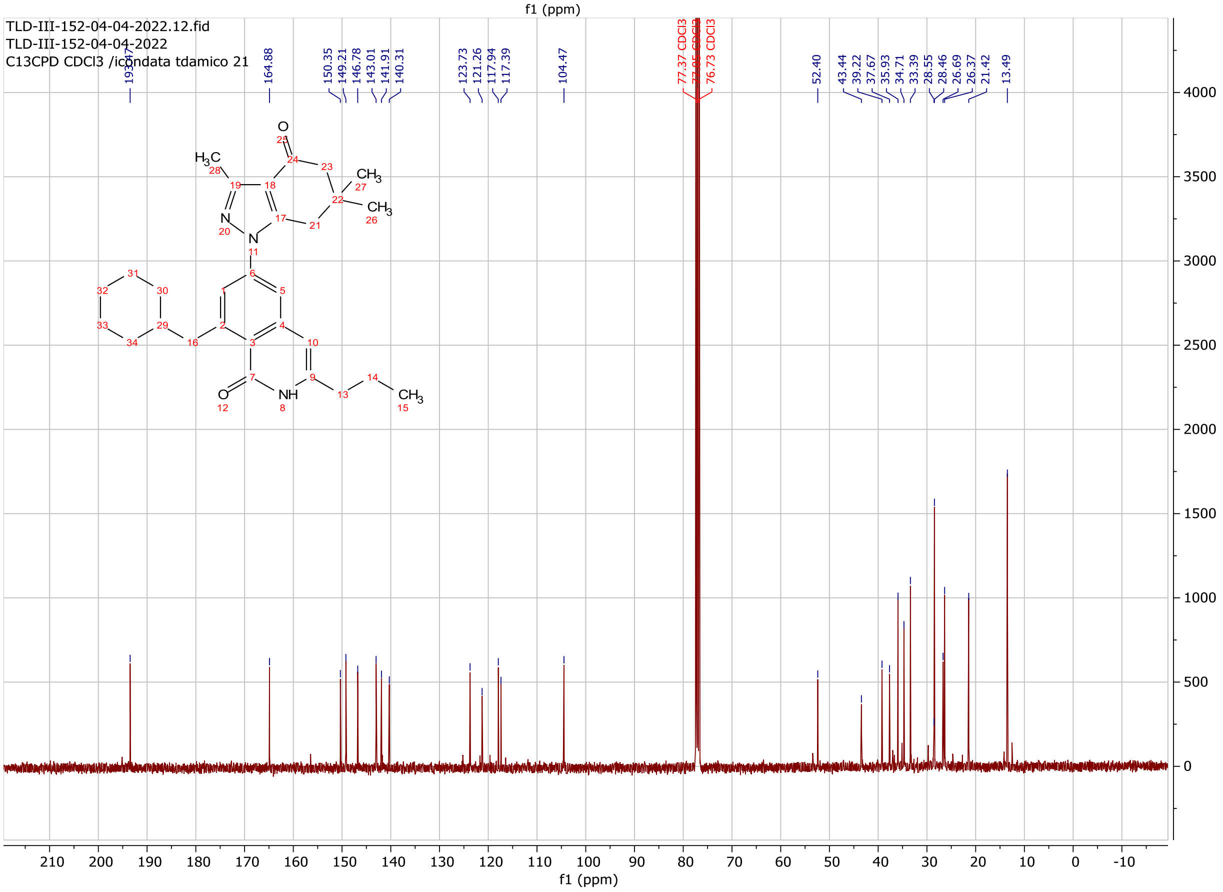

1.1.19. ^{1}H and ^{13}C NMR Spectra for **13h**

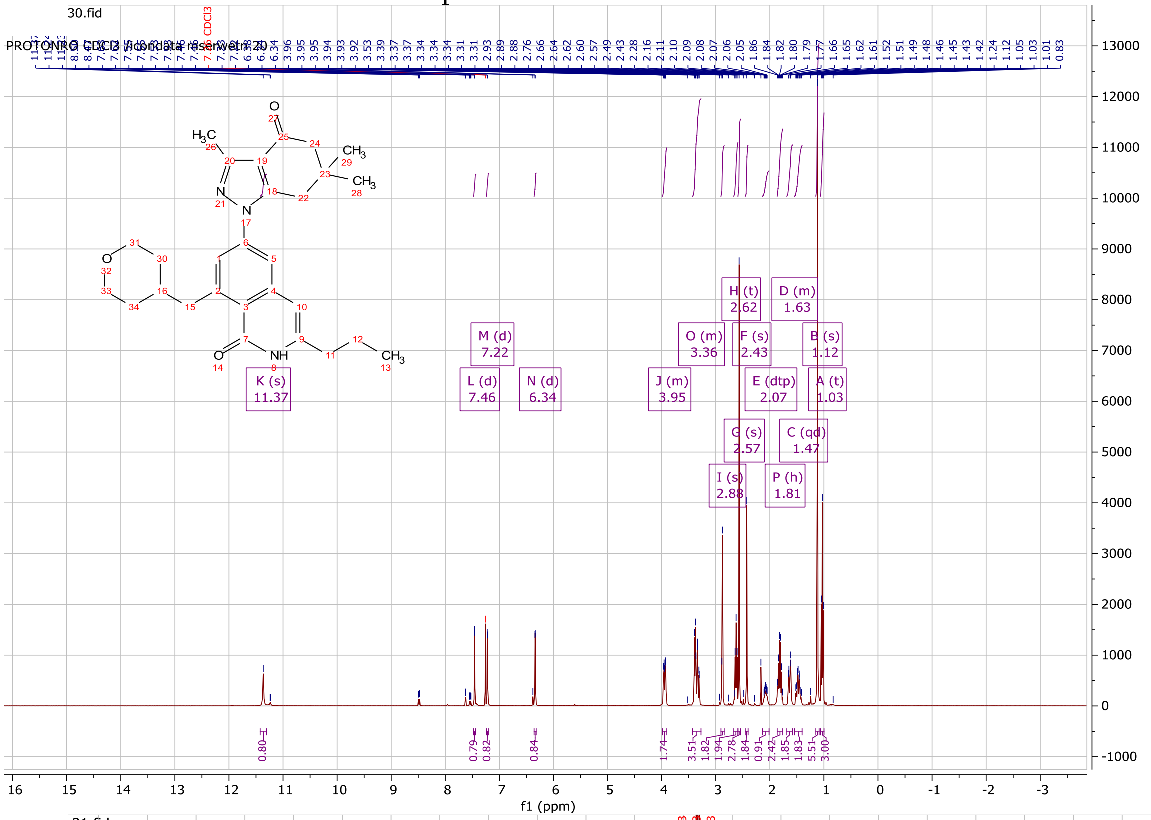

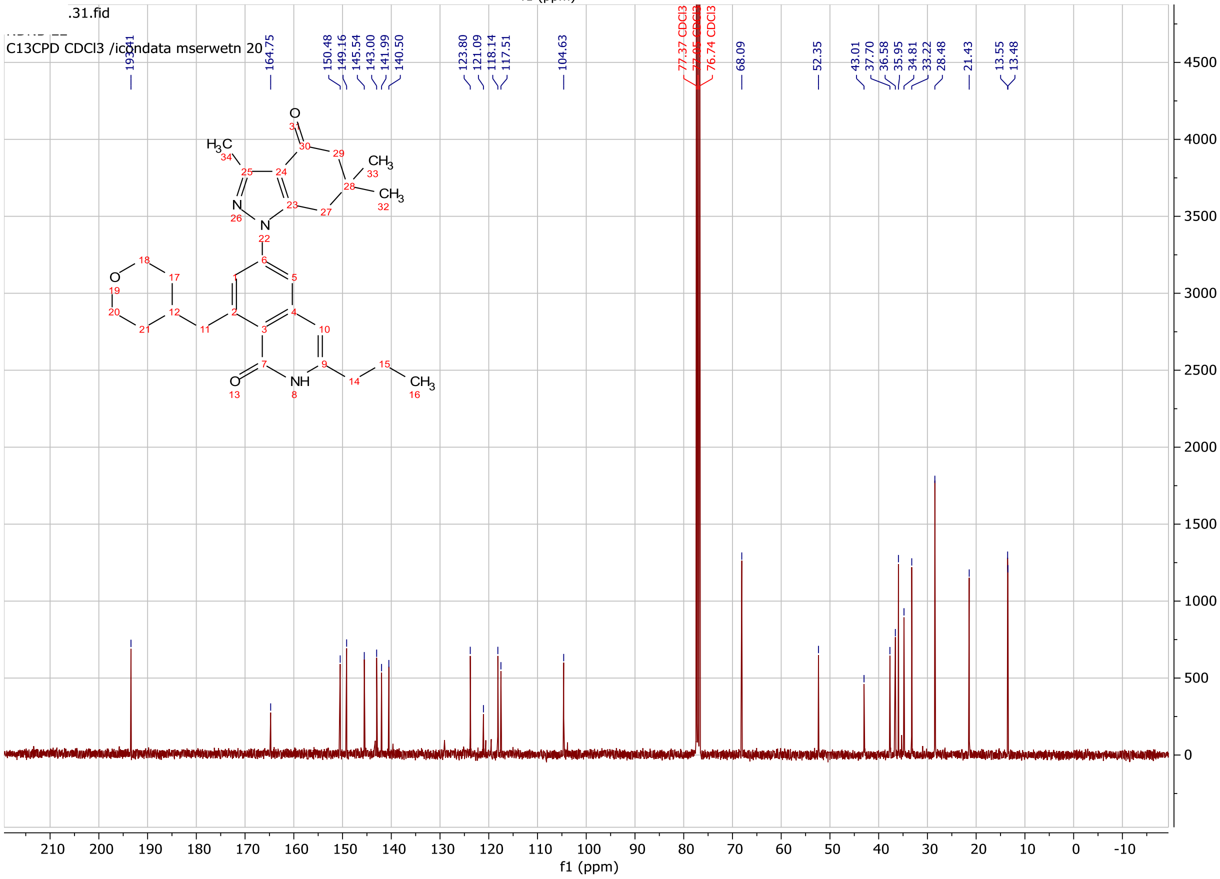

1.1.20. ^{1}H and ^{13}C NMR Spectra for **13i**

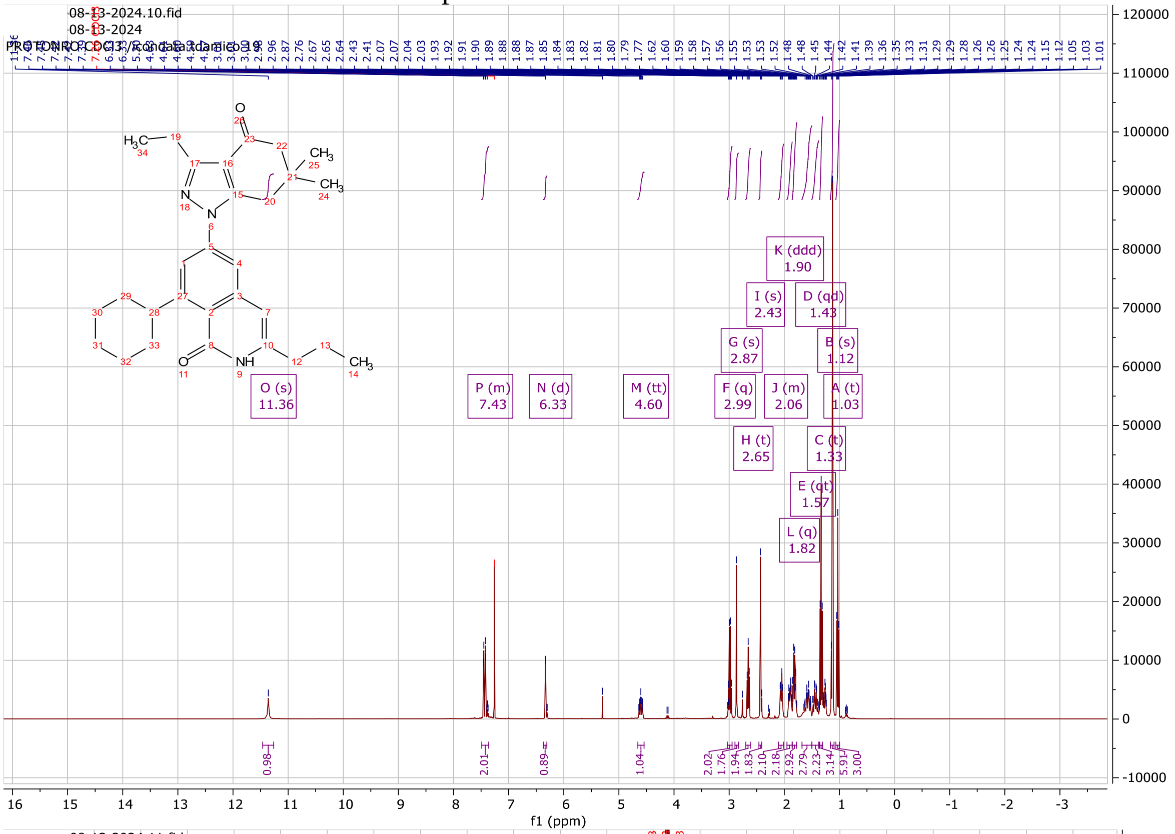

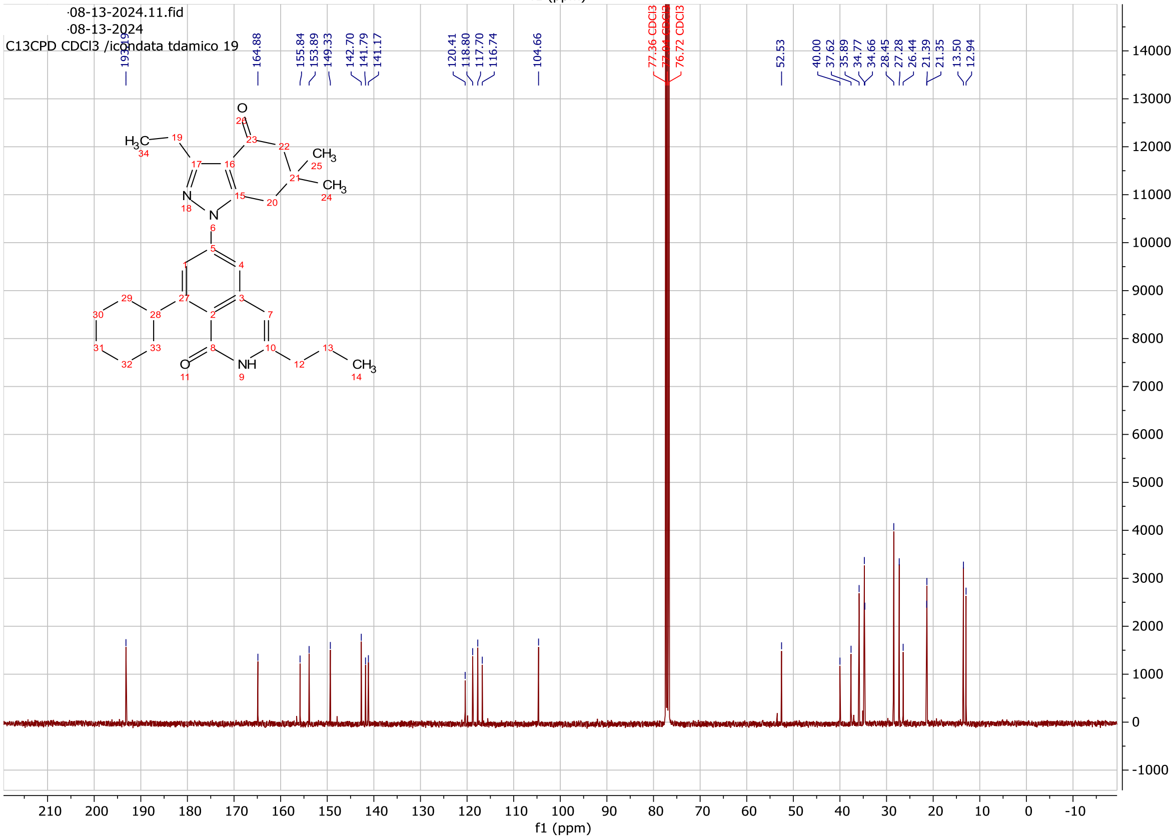

1.1.21. ^{1}H and ^{13}C NMR Spectra for **13j**

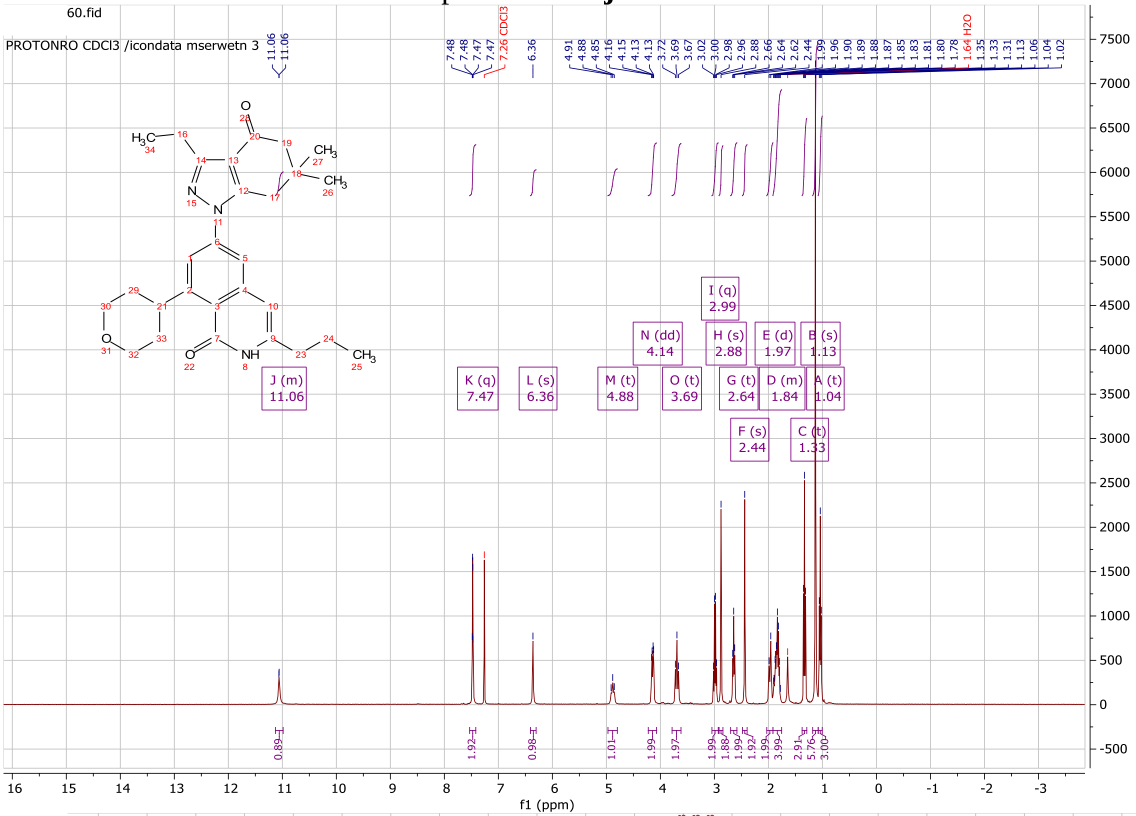

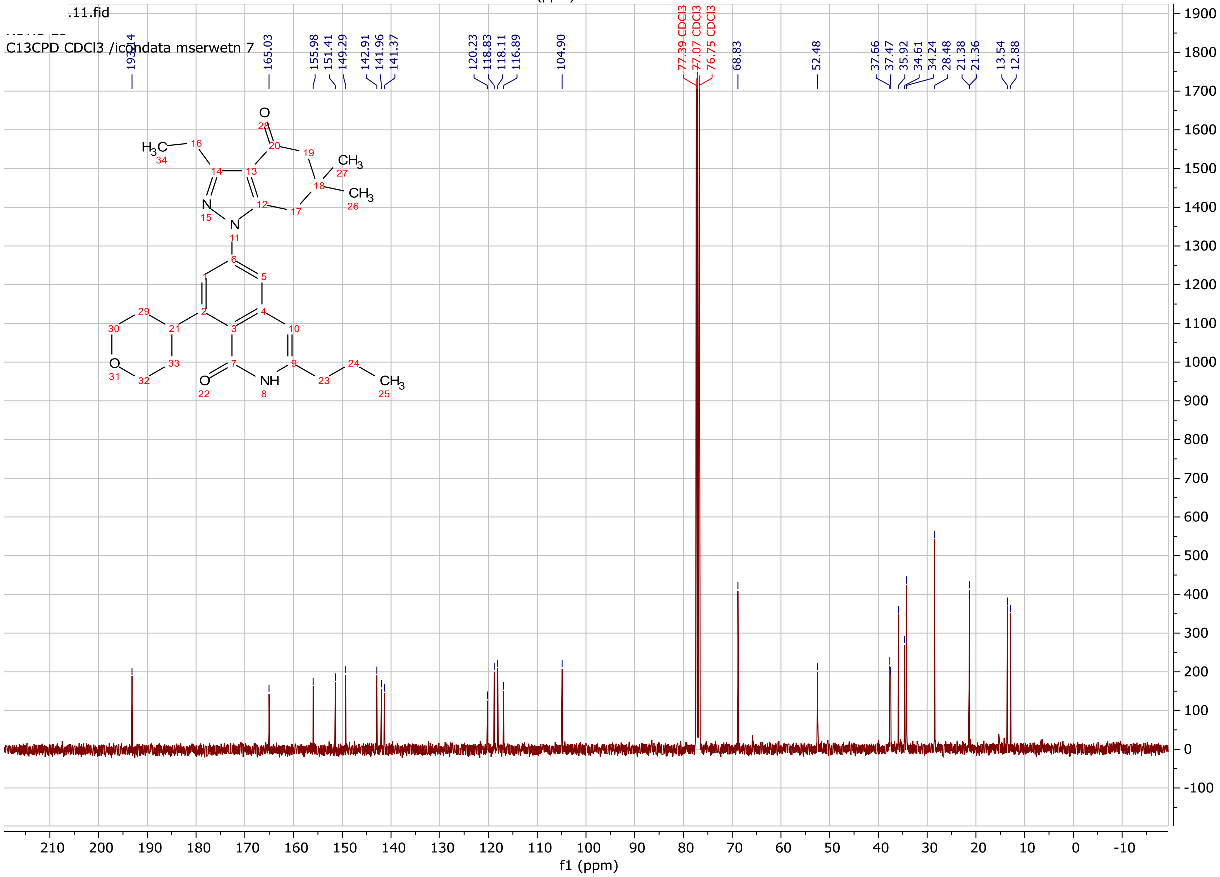

2. Characterization and Evaluation of Select 2*H*-Indazolone Addition Products

2.1. Characterization of Select 2*H*-Indazolone Addition Products

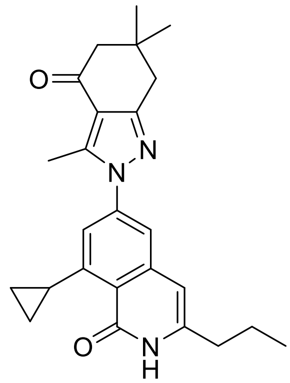

8-cyclopropyl-3-propyl-6-(3,6,6-trimethyl-4-oxo-4,5,6,7-tetrahydro-2H-indazol-2-yl)isoquinolin-1(2H)-one. Yield 5%; 6 mg. ^{1}H NMR (400 MHz, Chloroform-*d*) δ 10.70 (s, 1H), 7.32 (d, *J* = 2.1 Hz, 1H), 7.03 (d, *J* = 2.1 Hz, 1H), 6.27 (d, *J* = 1.7 Hz, 1H), 3.67 (tt, *J* = 8.4, 5.6 Hz, 1H), 2.75 (s, 2H), 2.64 (s, 2H), 2.57 (t, *J* = 7.8 Hz, 2H), 2.40 (s, 2H), 1.78 (hept, *J* = 7.6 Hz, 2H), 1.14 (s, 8H), 1.00 (t, *J* = 7.3 Hz, 3H), 0.85 – 0.77 (m, 2H). ^{13}C NMR (101 MHz, $CDCl_3$) δ 195.08, 164.69, 156.44, 149.36, 142.78, 141.84, 141.41, 141.14, 122.60, 118.98, 118.85, 116.40, 104.38, 53.42, 37.00, 35.10, 28.54 (2), 21.52, 14.89, 13.57, 12.48, 9.87 (2). HRMS (ESI) m/z [M + H] calc'd for $C_{25}H_{30}N_3O_2$, 404.2333, found 404.2336. R_f = 0.36 (50% ethyl acetate in hexanes).

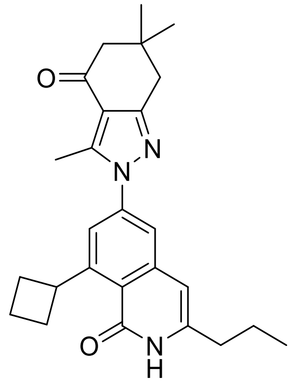

8-cyclobutyl-3-propyl-6-(3,6,6-trimethyl-4-oxo-4,5,6,7-tetrahydro-2H-indazol-2-yl)isoquinolin-1(2H)-one. Yield 3%; 4 mg. ^{1}H NMR (400 MHz, Chloroform-*d*) δ 11.27 (s, 1H), 7.45 (d, *J* = 2.2 Hz, 1H), 7.37 (d, *J* = 2.2 Hz, 1H), 6.29 (s, 1H), 4.89 (p, *J* = 8.6 Hz, 1H), 2.77 (s, 2H), 2.69 (s, 3H), 2.62 (t, *J* = 7.6 Hz, 2H), 2.51 (qt, *J* = 7.4, 2.2 Hz, 2H), 2.41 (s, 2H), 2.16 (pd, *J* = 8.7, 8.2, 2.2 Hz, 2H), 2.10–1.98 (m, 1H), 1.84 (h, *J* = 7.7 Hz, 3H), 1.15 (s, 6H), 1.06 (t, *J* = 7.4 Hz, 3H). ^{13}C NMR (101 MHz, $CDCl_3$) δ 195.11, 164.37, 156.47, 150.35, 143.03, 141.91, 141.61, 141.09, 121.06, 120.47, 119.36, 116.42, 104.41, 53.44, 39.77, 37.03, 35.12 (2), 29.47 (2), 28.56 (2), 21.61, 18.09, 13.68, 12.53. HRMS (ESI) m/z [M + H] calc'd for $C_{26}H_{32}N_3O_2$, 418.2489, found 418.2486. R_f = 0.50 (50% ethyl acetate in hexanes).

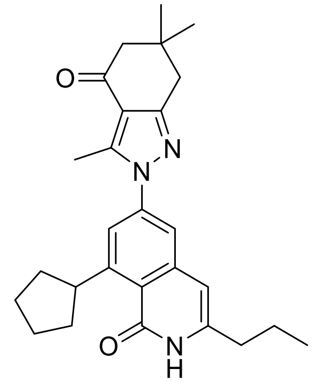

8-cyclopentyl-3-propyl-6-(3,6,6-trimethyl-4-oxo-4,5,6,7-tetrahydro-2H-indazol-2-yl)isoquinolin-1(2H)-one. Yield 4%; 7 mg. ^{1}H NMR (400 MHz, Chloroform-*d*) δ 11.24 (s, 1H), 7.45 (d, *J* = 2.1 Hz, 1H), 7.38 (d, *J* = 2.2 Hz, 1H), 6.30 (s, 1H), 4.94 (p, *J* = 8.8 Hz, 1H), 2.76 (s, 2H), 2.67 (s, 3H), 2.60 (t, *J* = 7.5 Hz, 2H), 2.41 (s, 2H), 2.23 (h, *J* = 5.7, 5.3 Hz, 2H), 1.81 (dq, *J* = 15.2, 7.7, 7.1 Hz, 6H), 1.65 (q, *J* = 7.5 Hz, 2H), 1.15 (s, 6H), 1.02 (t, *J* = 7.3 Hz, 3H). ^{13}C NMR (101 MHz, $CDCl_3$) δ 195.10, 162.09, 156.45, 152.25, 142.66, 141.85, 141.57, 141.05, 121.42, 120.02, 119.34, 116.44, 104.75, 53.44, 42.04, 37.04, 35.11, 34.93, 34.48 (2), 28.55 (2), 25.52 (2), 21.48, 13.59, 12.51. HRMS (ESI) m/z [M + H] calc'd for $C_{27}H_{34}N_3O_2$, 432.2646, found 432.2650. R_f = 0.60 (50% ethyl acetate in hexanes).

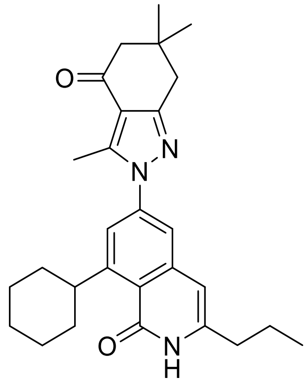

8-cyclohexyl-3-propyl-6-(3,6,6-trimethyl-4-oxo-4,5,6,7-tetrahydro-2H-indazol-2-yl)isoquinolin-1(2H)-one. Yield 4%; 6 mg. ^{1}H NMR (400 MHz, Chloroform-*d*) δ 10.51 (s, 1H), 7.43 (d, *J* = 2.1 Hz, 1H), 7.39 (d, *J* = 2.2 Hz, 1H), 6.31 (s, 1H), 4.55 (t, *J* = 11.8 Hz, 1H), 2.77 (s, 2H), 2.68 (s, 3H), 2.60 (q, *J* = 6.7, 5.9 Hz, 2H), 2.41 (s, 2H), 2.02 (d, *J* = 11.9 Hz, 2H), 1.88 (d, *J* = 12.9 Hz, 2H), 1.79 (h, *J* = 7.2 Hz, 3H), 1.57 (q, *J* = 12.9 Hz, 2H), 1.49–1.36 (m, 2H), 1.34–1.27 (m, 1H), 1.15 (s, 6H), 1.02 (t, *J* = 7.4 Hz, 3H). ^{13}C NMR (101 MHz, $CDCl_3$) δ 195.09, 164.98, 156.47, 153.91, 149.30, 142.25, 141.88, 141.20, 120.26, 119.30, 116.46, 104.85, 53.44, 39.96, 37.04, 35.11, 34.77, 34.75 (2), 28.55 (2), 27.19 (2), 26.42, 21.29, 13.49, 12.53. HRMS (ESI) m/z [M + H] calc'd for $C_{28}H_{36}N_3O_2$, 446.2802, found 446.2808. R_f = 0.63 (50% ethyl acetate in hexanes).

2.2. NMR Spectra of Select 2*H*-Indazolone Addition Products

2.2.1. ^{1}H and ^{13}C NMR Spectra for Cyclopropyl Derivative

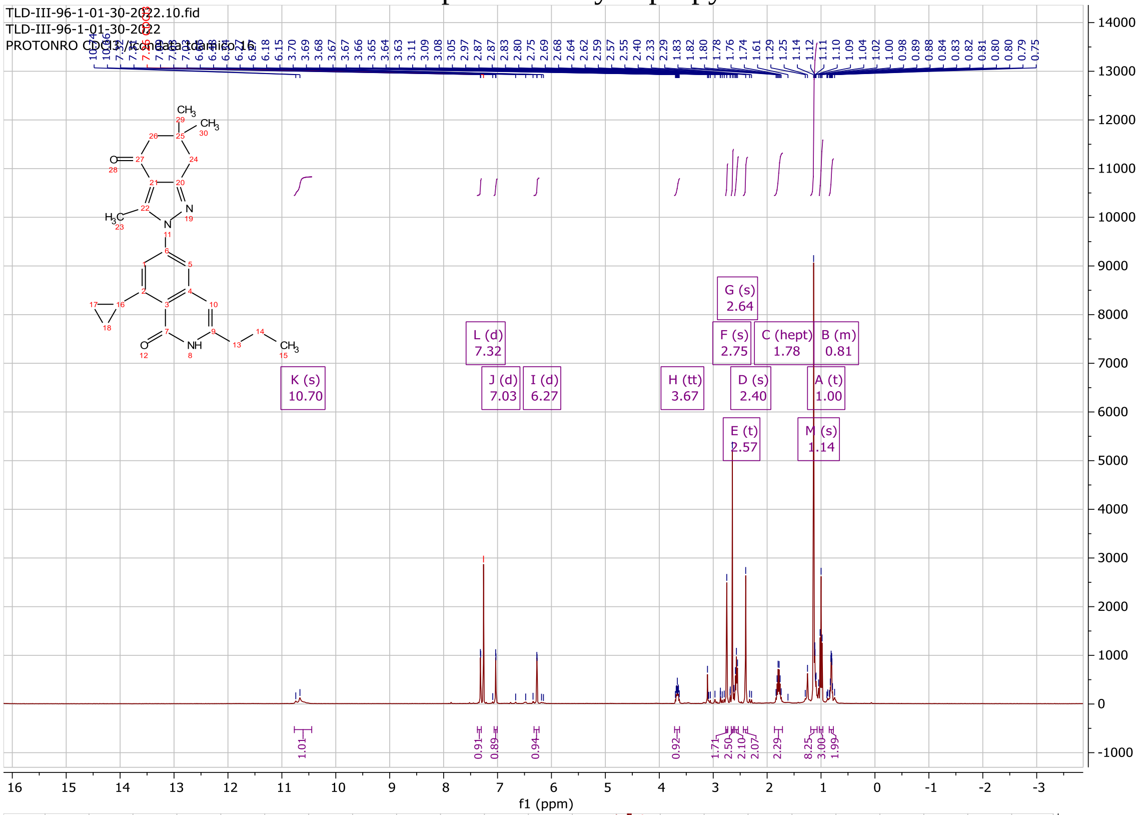

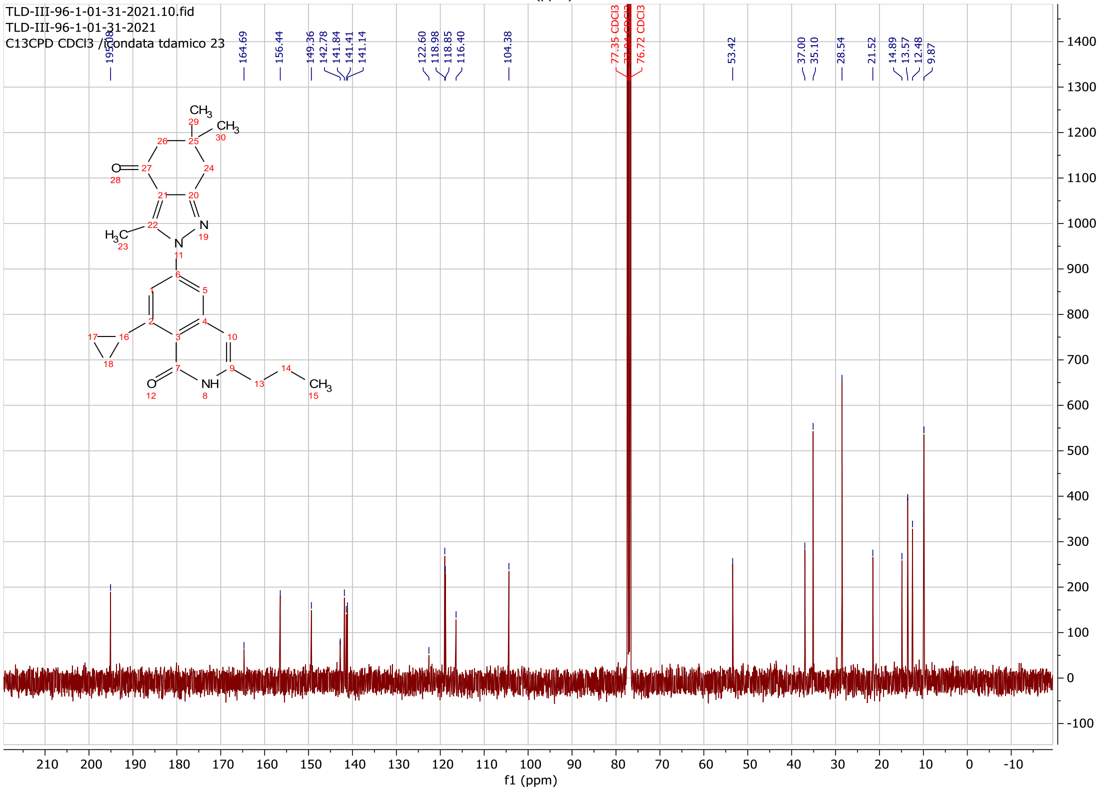

2.2.2. ^{1}H and ^{13}C NMR Spectra for Cyclobutyl Derivative

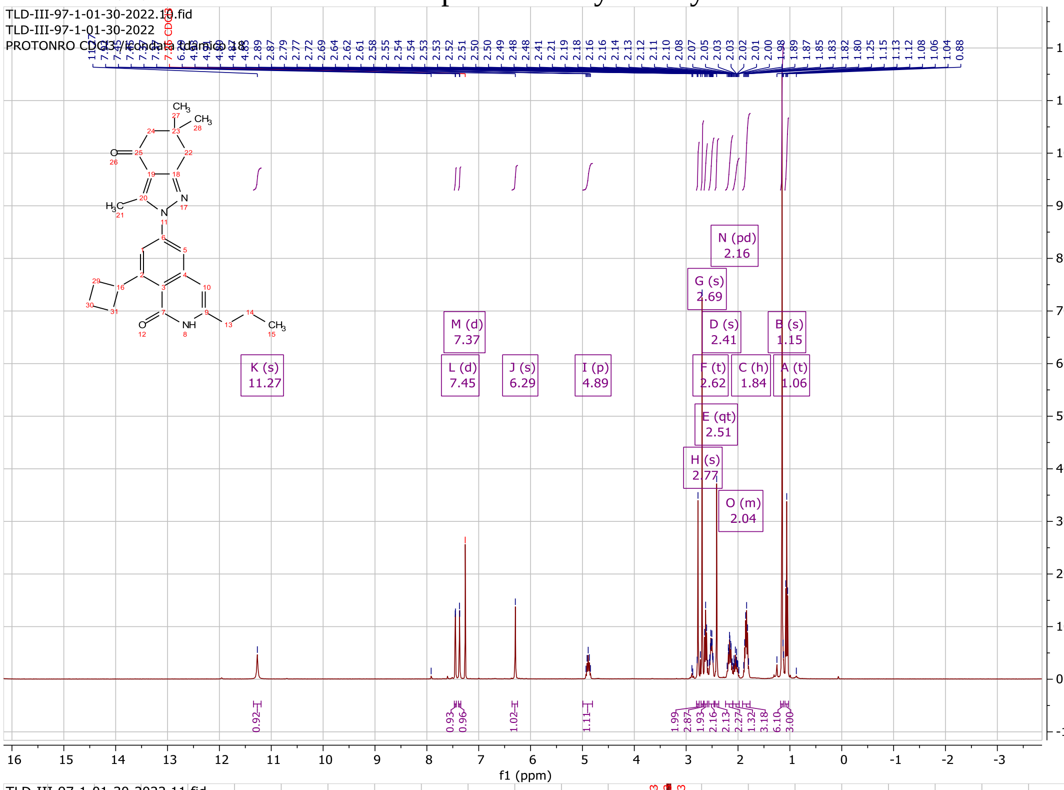

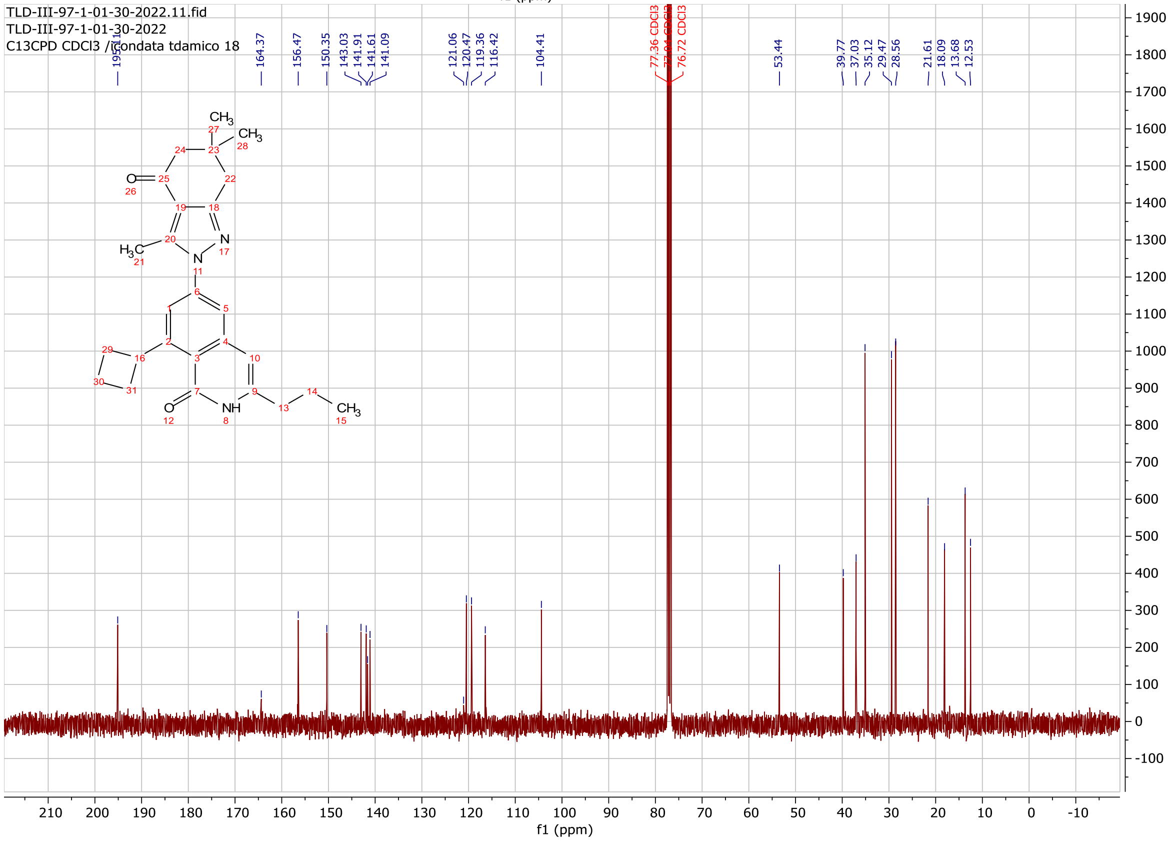

2.2.3. ^{1}H and ^{13}C NMR Spectra for Cyclopentyl Derivative

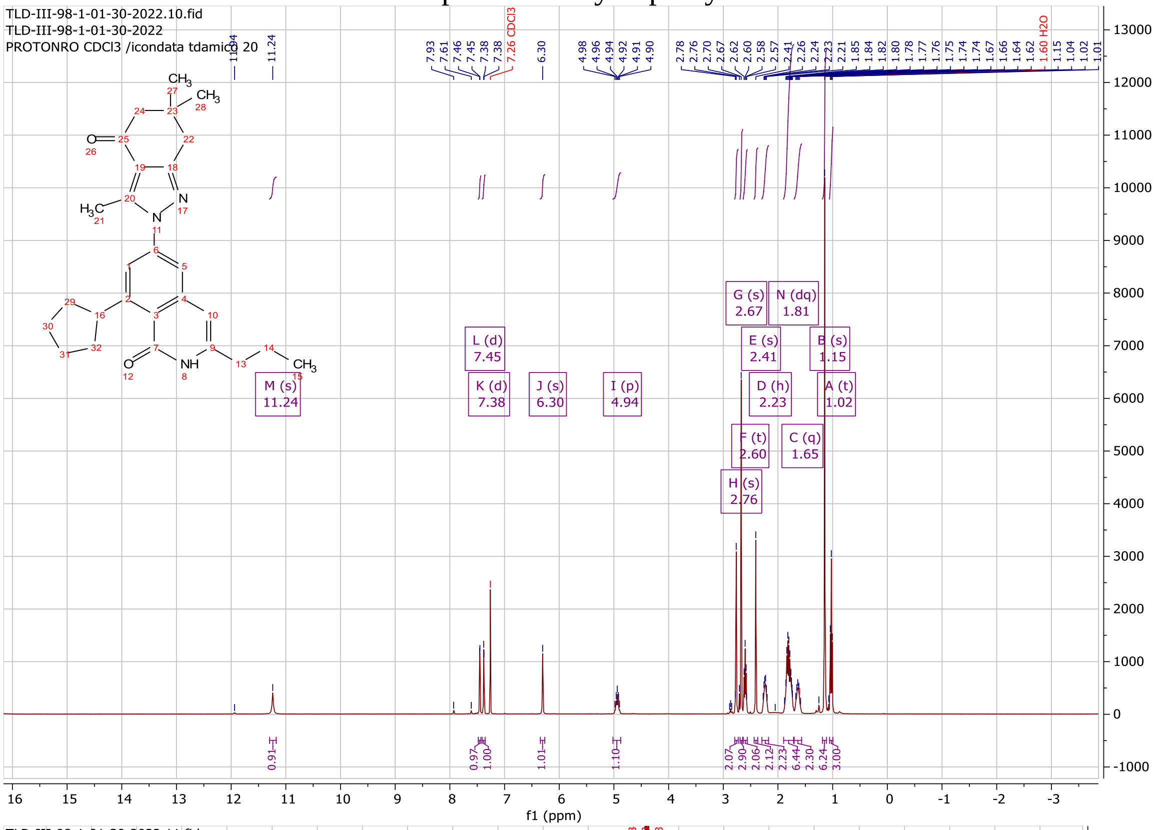

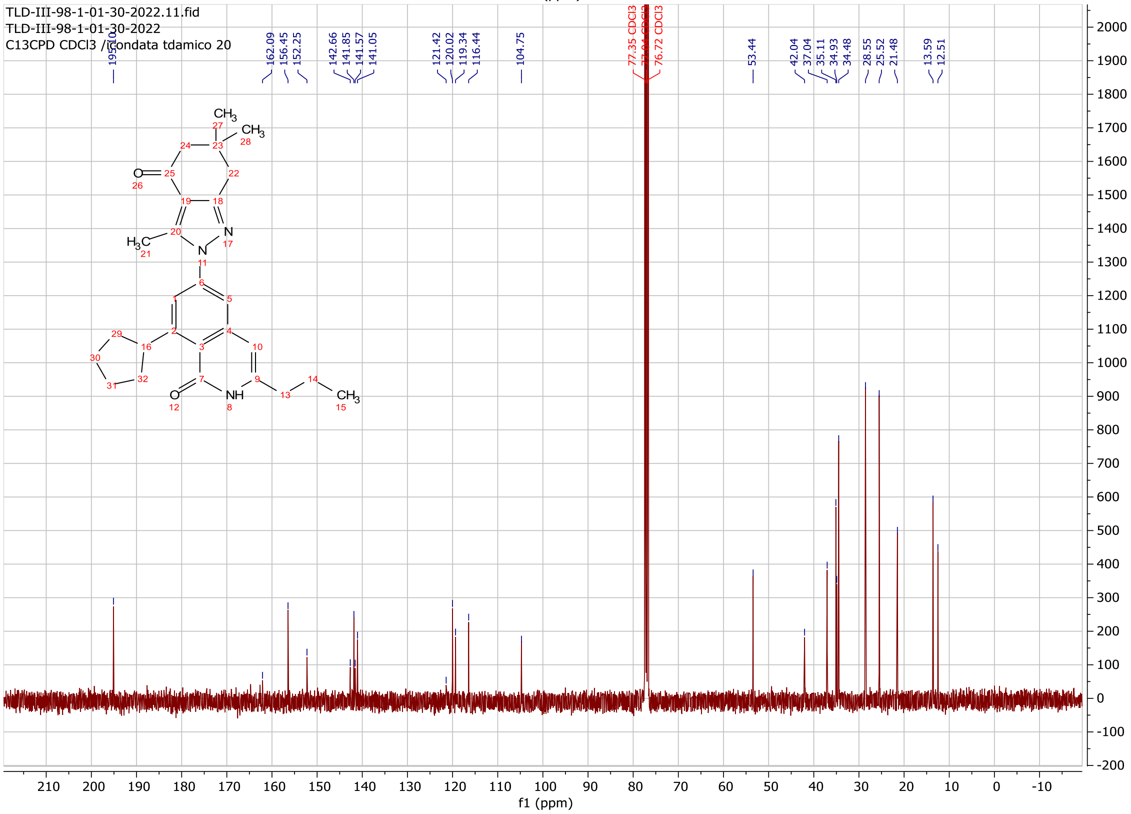

2.2.4. ^{1}H and ^{13}C NMR Spectra for Cyclohexyl Derivative

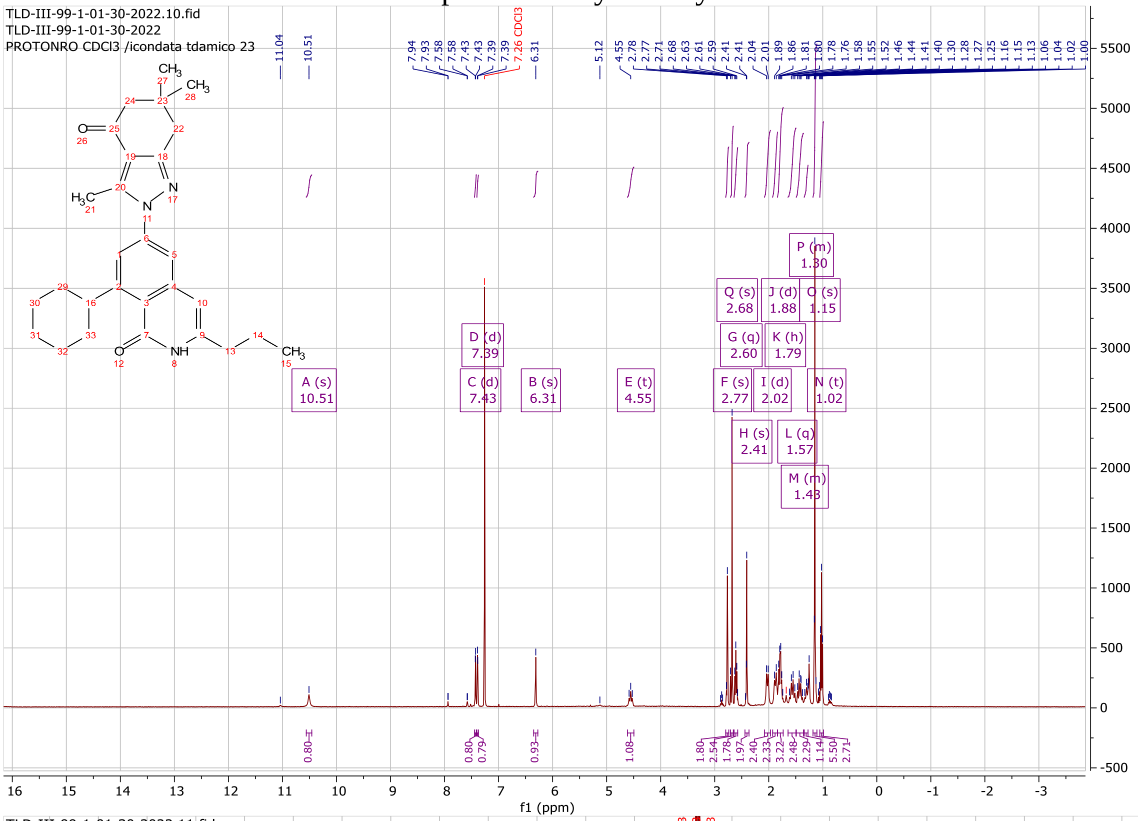

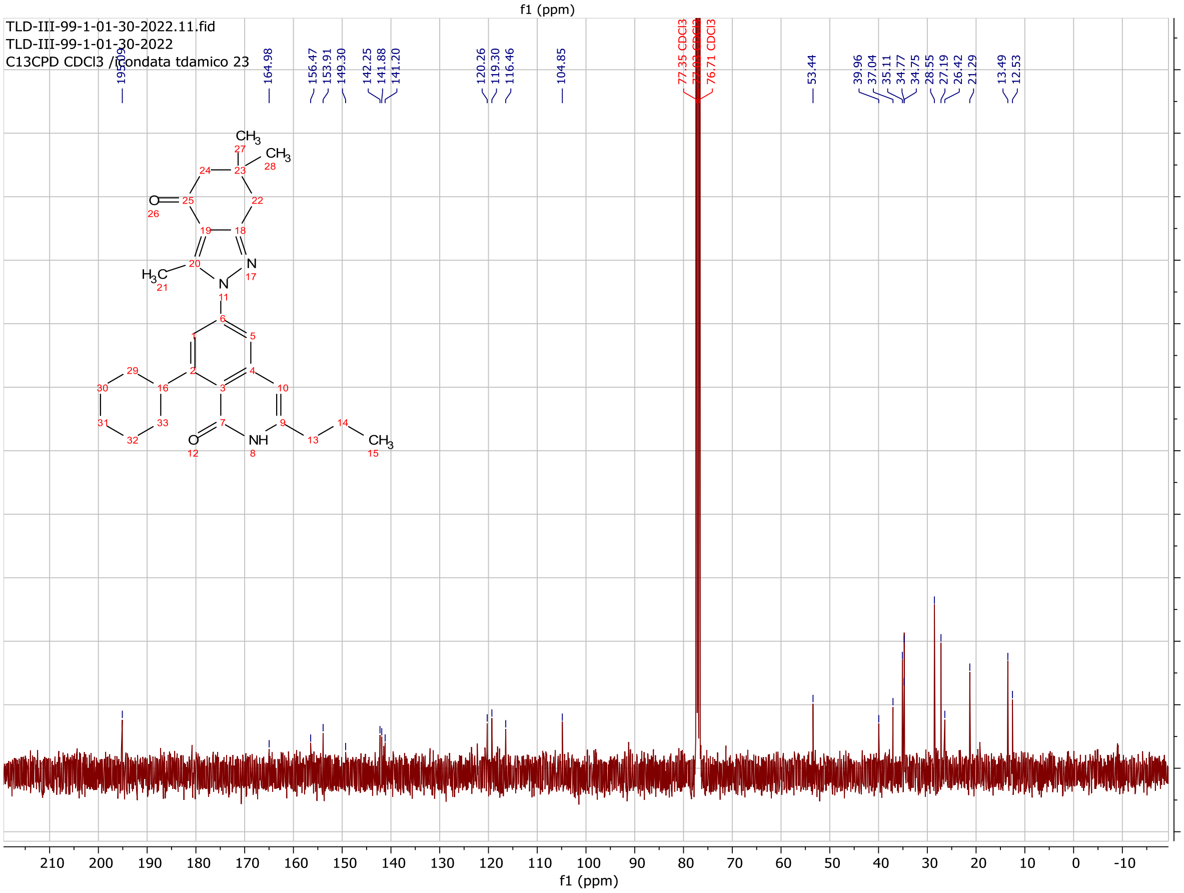

2.3. Evaluation of Select 2*H*-Indazolone Addition Products *via* the Fluorescence Polarization (FP) Assay

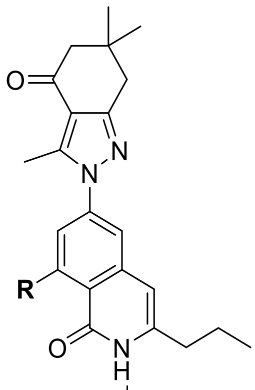

R	Hsp90*α* K_D (*μ*M)	Hsp90*β* K_D (*μ*M)	Hsp90*β* Selectivity
Cyclopropyl	>100	>100	Not Significant
Cyclobutyl	>100	>100	Not Significant
Cyclopentyl	>100	>100	Not Significant
Cyclohexyl	>100	>100	Not Significant

3. References

1. Serwetnyk, M. A.; Strunden, T.; Mersich, I.; Barlow, D.; D'Amico, T.; Mishra, S. J.; Houseknecht, K. L.; Streicher, J. M.; and Blagg, B. S. J. Optimization of an Hsp90*β*-selective inhibitor *via* exploration of the N-terminal ATP-binding pocket. *Eur. J. Med. Chem.* **2025**, *297*, 117925. https://doi.org/10.1016/j.ejmech.2025.117925
2. Mishra, S. J.; Liu, W.; Beebe, K.; Banerjee, M.; Kent, C. N.; Munthali, V.; Koren III, J. Taylor III, J. A.; Neckers, L. M.; Holzbeierlein, J.; Blagg, B. S. J. The development of Hsp90β-selective inhibitors to overcome detriments associated with *pan*-Hsp90 inhibition. *J. Med. Chem.* **2021**, *64* (3), 1545–1557. https://doi.org/10.1021/acs.jmedchem.0c01700
3. Lee, J. C.; Hong, K. H.; Becker, A.; Tash, J. S.; Schönbrunn, E.; Georg, G. I. Tetrahydroindazole inhibitors of CDK2/cyclin complexes. *Eur. J. Med. Chem.* **2021**, *214*, 113232. https://doi.org/10.1016/j.ejmech.2021.113232
